# From Charge‐Transfer Adducts to (Tri)Halide‐Templated Supramolecular Cages: Covalent vs Ionic Products in the Reactions of Pyridin‐4‐ylthio‐Substituted Tetrathiafulvalene With Dihalogens and Interhalogens

**DOI:** 10.1002/chem.71483

**Published:** 2026-08-05

**Authors:** Anna Caria, Stéphane A. Baudron, M. Carla Aragoni, Vito Lippolis, Anna Pintus, Enrico Podda, Massimiliano Arca

**Affiliations:** ^1^ Dipartimento Di Scienze Chimiche e Geologiche Università Degli Studi di Cagliari Cagliari Italy; ^2^ Université De Strasbourg, CNRS, CMC UMR 7140 Strasbourg France; ^3^ Centro Servizi di Ateneo per La Ricerca (CeSAR) Università Degli Studi di Cagliari Cagliari Italy

**Keywords:** charge transfer, density functional calculations, halogens, supramolecular chemistry, TTF

## Abstract

The reactivity of tetra(pyridin‐4‐ylthio)tetrathiafulvalene (**L_1_**) toward dihalogens (Br_2_, I_2_)/interhalogens (ICl, IBr) was investigated by crystallographic, microanalytical, spectroscopic, and hybrid‐DFT approaches. A divergence in reactivity was observed depending on the halogen reagent. The reactions of **L_1_** with I_2_ yielded a 1:4 adduct **L_1_** ·4I_2_ (**1**), featuring four independent N···I─I interactions within a single polytopic scaffold. Reactions of **L_1_** with Br_2_, ICl, and IBr afforded ionic species (**2–6**), containing the protonated donor counterbalanced by discrete tri(inter)halide anions such as [Br_3_]^−^, [ICl_2_]^−^, and [IBr_2_]^−^. These compounds showed diverse supramolecular cage‐like architectures, often involving hydrogen‐bonded networks. [H_4_ **L_1_**](**L_1_**)[IBr_2_]_4_·2CH_3_CN·2.4CHCl_3_ (**4**) contains a tetracationic capsule [(H_4_ **L_1_**)(**L_1_**)]^4+^ stabilized by N–H···N HBs and templated at a [IBr_2_]^−^ guest. Both the N···I─I fragment in compound **1** and the tri(inter)halides in compounds **2–6** follow a common structural correlation between the normalized bond elongations typical of three‐body systems. Hybrid‐DFT QTAIM, NBO, Hirshfeld, and Bader charges, along with NCI index calculations allowed to quantify the CT interactions and the extent of ionic/covalent contributions to the HaBs in compound **1**, analysis of the anion–cation interactions in the inclusion complex [(H_4_ **L_1_**)(**L_1_**)(IBr_2_)]^3+^, and reproduction of the observed structural trends, highlighting the continuous nature of covalent‐to‐electrostatic interactions in apparently different three‐body systems.

## Graphical abstract

The reactions between tetra(pyridin‐4‐ylthio)tetrathiafulvalene and Br_2_/I_2_/ICl/IBr led to a variety of products, including a rare example of neutral 1:4 CT adduct and ionic products showing supramolecular cage motifs at (tri)halide templates. The nature of the reaction products was elucidated based on XRD analysis, Raman spectroscopy and QTAIM, NCI, and NBO hybrid‐DFT calculations.

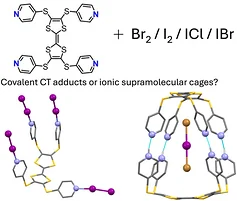

## 1 Introduction

The cooperativity and competition between directional noncovalent interactions (NCI) such as hydrogen bonds (HBs) and halogen bonds (HaBs) have long been investigated to establish interaction hierarchies among multifunctional synthons, with the aim of predicting and controlling crystal growth, self‐assembly processes, and the properties of the resulting crystalline solids and materials. In this context, unsaturated N‐heterocycles represent a broad class of ligands for the rationale molecular design, owing to their Lewis basicity toward a variety of acids, including transition metal ions, dihalogens, and interhalogens. As regards dihalogens X_2_ and interhalogens XY (X, Y = Cl, Br, and I), a variety of reaction products have been isolated and characterized over the past decades.

The direct interaction of N‐heterocycles, such as pyridine (Py), with dihalogens/interhalogens leads to η^1^‐adducts featuring a linear N···X─Y “spoke” moiety (X = Br and I), similar to related adducts formed by chalcogen donors [1]. These adducts, the first example of which dates back to 1863 [2], have attracted a growing interest due to the variety of their applications in fields as diverse as organic synthesis and catalysis [3], liquid crystals [2], as well as crystal engineering and supramolecular chemistry [4, 5, 6, 7, 8, 9]. The formation of stoichiometric adducts has also found application in the design of semiconductor materials [10], since adduct formation lowers the band‐gap and enhances the solid‐state conductivity of the resulting material as compared to the starting donor [11]. A common structural feature of this class of compounds is the N···X distance remarkably shorter than the sum of the relevant van der Waals (vdW) radii and an elongation of the bonded dihalogen/interhalogen relative to the corresponding X─Y bond length in the free species.

A second class of products obtained from the reaction of pyridine donors with dihalogens is represented by halogen(I) compounds displaying a N─X^+─^N linear fragment (X = Br and I), whose formation has been related to the instability of CT adducts with respect to ionic dissociation. Iodonium N‐complexes are mild oxidants, as exemplified by Barluenga's reagent, (Py)_2_I(BF_4_) [12, 13], which is obtained by reacting pyridine and diiodine in the presence of stoichiometric amounts of AgBF_4_ on silica [14, 15].

A third common type of reaction product features the protonated N‐donor counterbalanced by the halides X^–^. Although the protonation mechanism of such species has not been clearly elucidated, it has been proposed that their formation may occur either from CT adducts [16] or via oxidation of the aromatic heterocycle to a radical cation, followed by solvolysis or reaction with incipient moisture [17]. Worthy of note, in the presence of an excess of dihalogens, the bulky N‐protonated cation can template extended and infinite anionic networks [4, 17, 18, 19, 20].

Finally, dihalogens/interhalogens can oxidize the (poly)pyridine donor, leading to fascinating products that cannot be obtained by other synthetic routes, as exemplified by the reaction of bis(pyridin‐2‐yl)ditellane (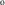
Py_2_Te_2_) with Br_2_ in acetonitrile [21].

In this context, the wide variety of possible reaction products renders polypyridine derivatives of particular interest, as they can either form adducts with variable donor/X_2_ molar ratios or undergo protonation. In the latter case, the large size of the resulting cations can stabilize unusual polyhalides. For example, bis(pyridin‐2‐yl)dichalcogenides 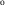
Py_2_Ch_2_ (Ch = S and Se) made to react with I_2_ formed compounds with stoichiometry [H(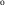
Py_2_Ch_2_)(I)·5/2I_2_]_∞_ [22, 23], in which the N‐monoprotonated cation [H(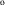
Py_2_Ch_2_)]^+^ is counterbalanced by iodide ions connected by diiodine molecules, with all I···I_2_ distances shorter than 3.6 Å [24, 25, 26], thereby forming a self‐assembled infinite three‐dimensional polyiodide network. Notably, the diprotonated cation [(H^p^Py)_2_S_2_]^2+^ (^p^Py = pyridin‐4‐yl) was isolated in the crystal structure of [(H^p^Py)_2_S_2_](Cl)(I_3_) [27]. Analogously, the reaction of 2,4,6‐tris(2‐pyridyl)‐1,3,5‐triazine (tptz) with IBr yielded (H_3_tptz)(I_3_Br_4_)(IBr_2_)_2_, featuring an extended [I_5_Br_8_]^2−^ anion resulting from the interaction between [I_3_Br_4_]^−^ and [IBr_2_]^−^ anions [7].

In this context, polypyridyl systems represent particularly attractive platforms, as they can simultaneously act as multidentate Lewis bases, undergo protonation, and stabilize extended polyhalide networks. Among these compounds, tetrathiafulvalene (TTF)‐based derivatives are of additional interest due to their redox activity and potential applications in molecular electronics. Some of the authors have recently reported a TTF derivative featuring four pyridin‐4‐ylthio substituents (**L_1_**, Scheme 1), that combines a TTF core with four pyridyl donor sites capable of adapting different conformations and coordination modes [28, 29].

**SCHEME 1:**
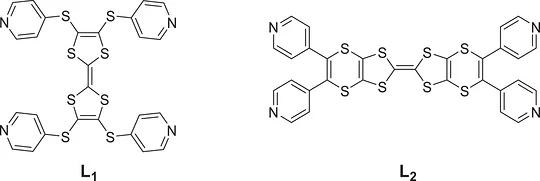
Chemical structures of the polypyridyl donors **L_1_** and **L_2_**.

Compound **L_1_** structurally recalls tetra(pyridin‐4‐yl)bis(vinylenedithio)tetrathiafulvalene (**L_2_**, Scheme 1) [30], which also features four pyridine substituents and is capable of forming coordination polymers (CPs). The Cd^II^‐based CP derived from compound **L_2_** was shown to react with I_2_ to form oxidized radical cations [**L_2_**]^·^^+^ within the metal‐organic framework (MOF) CdCl_2_·[**L_2_**][I_3_], displaying a conductivity one order of magnitude larger than that of **L_2_** [31].

In this study, we report an extensive investigation on the reactivity of **L_1_** toward a series of dihalogens and interhalogens (Br_2_, I_2_, ICl, and IBr), with the aim of understanding how the nature of the dihalogens/interhalogen controls the outcome of the reactions, and the formation of neutral charge‐transfer (CT) adduct and/or ionic products featuring protonated ligands counterbalanced by (inter)halide anions. By combining crystallographic, spectroscopic, and theoretical approaches, we show that these apparently distinct classes of compounds can be rationalized within a unified framework, in which halogen bonding interactions span a continuum from weak CT adducts to predominantly electrostatic ionic assemblies.

## 2 Results and Discussion

The polytopic ligand tetra(pyridin‐4‐ylthio)tetrathiafulvalene (**L_1_**) was made to react in CHCl_3_/CH_2_Cl_2_ or acetonitrile solution with solutions of dihalogens/interhalogens, namely Br_2_, I_2_, ICl, and IBr, which feature different absolute hardness *η* [32] (increasing in the order I_2_ > IBr > ICl > Br_2_) and oxidizing properties. Reactions were carried out in various molar ratios ranging from 0.5:1 to a large excess of the dihalogen, either by slow evaporation of the reaction mixtures or by diffusion of the dihalogen vapors into a solution of the N‐donor. When crystals suitable for X‐ray diffraction analysis could be collected [33], these were also analyzed by microanalytical and spectroscopic techniques. The reactivity of **L_1_** toward the investigated halogens/interhalogens follows two clearly distinct pathways (Scheme S1). The reactions of **L_1_** with diiodine selectively afforded the neutral “spoke” adduct **1**, whereas with Br_2_, ICl, and IBr, the ionic products **2–6** containing protonated ligands and tri(inter)halide anions were exclusively isolated and characterized. In the following, the relevant reactions will be discussed in relation to the oxidizing ability of the dihalogen.

### 2.1 Reactions of **L_1_** With I_2_

By reacting compound **L_1_** with I_2_ in different molar ratios (from 1:0.5 to 1:5) in acetonitrile or chloroform, single crystals of **L_1_** ·4I_2_ (**1**) suitable for single crystal X‐ray diffraction analysis were isolated (Tables S1–S3). Structural characterization revealed the formation of a CT “spoke” adduct, in which the four pyridyl rings of **L_1_** engage with four crystallographically independent I_2_ molecules (Figure 1 and Table 1). The identity of the bulk material were ascertained by powder x‐ray diffraction; the experimental pattern is in good agreement with that simulated from SC‐XRD data, indicating the presence of a single crystalline phase (Figure S1).

**FIGURE 1:**
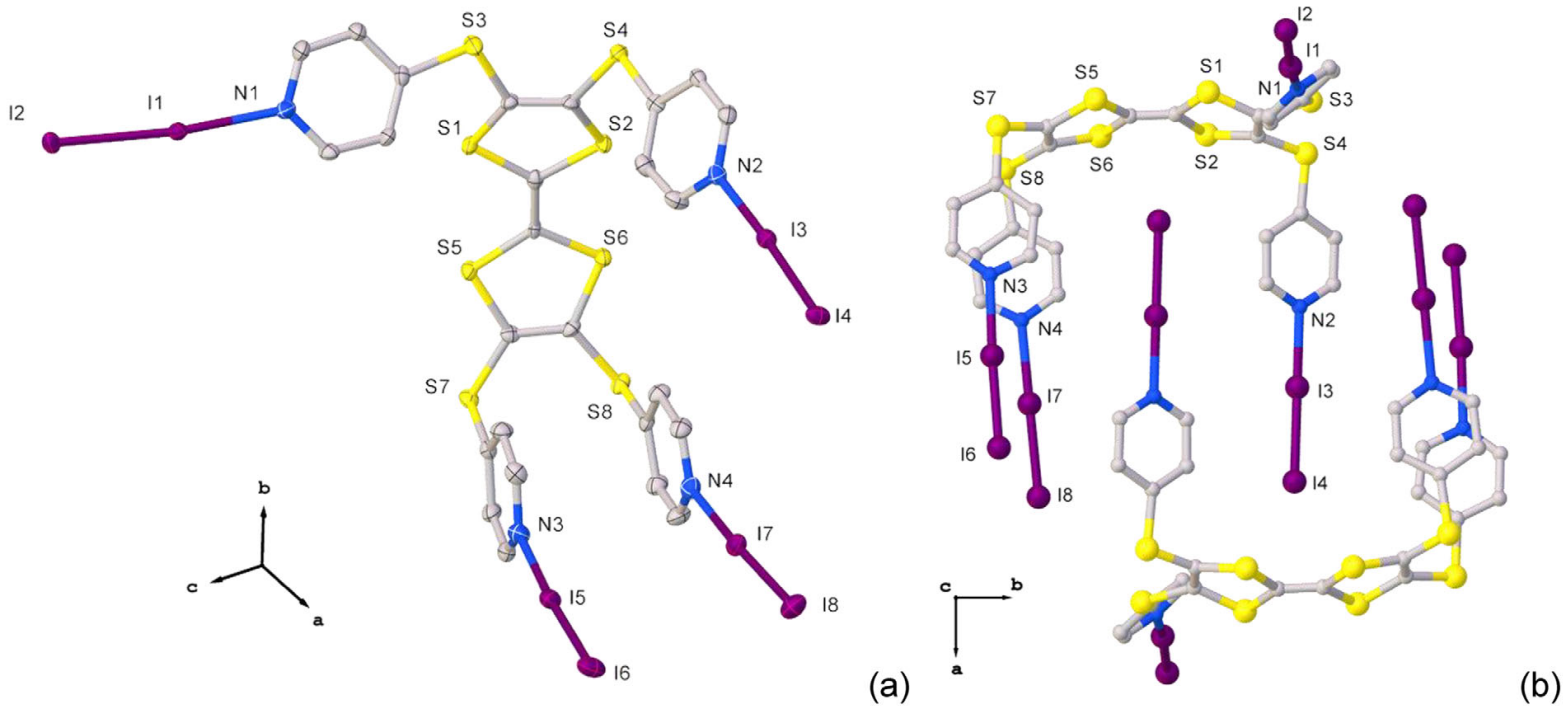
X‐ray crystal structure of compound **1** seen along the [111] direction (a); interdigitated dimers of symmetry related **L_1_** ·4I_2_ units (b). Only heteroatoms were labelled. Displacement ellipsoids were drawn at 50% probability level. Hydrogen atoms were omitted for clarity.

**TABLE 1:** Bond distances *d* (Å), angles *α* (°), and relative elongations *δ* (see Equation 1) for N···I─I moieties in compound **1**.

	*d*_N···I_	*d*_I─I_	*α*_N─I─I_	*δ*_N─I_	*δ*_I─I_
N1*···*I1─I2	2.441(5)	2.7879(6)	172.64(13)	0.197	0.0481
N2*···*I3─I4	2.379(4)	2.8013(6)	177.06(10)	0.166	0.0530
N3*···*I5─I6	2.454(5)	2.7663(6)	176.34(12)	0.203	0.0398
N4···I7─I8	2.488(6)	2.7674(6)	176.83(12)	0.220	0.0402

Worthy of note, the **L_1_** ' isomer donor, featuring four pyridin‐2‐ylthio substituents, reportedly reacts with I_2_ to give the oxidized [**L_1_** ']^2+^ cation counterbalanced by triiodides in the compound [**L_1_** ′](I_3_)_2_ [34]. A search in the Cambridge Structural Database (CSD) [35] indicated that authentic Py···I─I η^1^‐adducts are relatively uncommon, with only 29 examples reported to date (Scheme S2). The geometrical parameters confirm the linearity of the N─I─I angle *α* [mean value 176(2)°], with N···I distances ranging from 2.322 to 2.659 Å [mean value 2.48(9) Å] and I─I distances between 2.732 and 2.849 Å [mean value 2.78(3) Å]. Interestingly, compound **1** represents a rare example of a CT adduct with a 1:4 donor/I_2_ ratio, the highest so far reported, whereas most previously reported structures show 1:1 or 1:2 stoichiometries (see Scheme S2 for the pyridyl donors whose I_2_ CT adducts have been structurally characterized). A related case was reported by Pennington for 2,3,5,6‐tetra(2′‐pyridyl)pyrazine, which formed a 1:6 adduct with I_2_, featuring four Py···I_2_ interactions and two additional I_2_ molecules interacting with the bonded iodine moieties [36]. As expected, the I─I distances in compound **1** are slightly elongated with respect to solid‐state I_2_ [1.9%–3.2%; I─I = 2.715(6) Å in solid‐state I_2_] [37]; such elongations classify these as weak I_2_ adducts. Among the four independent adducts, the N2···I3─I4 moiety exhibits the strongest interaction, as evidenced by the largest I─I elongation with respect to the solid‐state I─I distance [I3─I4 = 2.8013(6) Å] and the shortest N···I distance. Worthy of note, the I3─I4 distance is very close to those featured in the Py·I_2_ adduct [38].

As regards the conformation assumed by **L_1_** in compound **1**, in a previous study some of the authors explored the conformational properties of **L_1_** in the coordination chemistry landscape [28]. Ideally, six conformations can be envisioned, labelled α_2_α_2_, βαα_2_, αβα_2_, β_2_α_2,_ αβαβ, and βααβ depending on the relative orientation of the pyridylthio arms with respect to the tetrathiafulvalene plane (Scheme S3) [28]. Reactions with Cd^II^ salts afforded CPs [28, 39, 40] in which **L_1_** demonstrated its flexibility, adopting four out of the six possible limit conformations, namely α_2_α_2_, β_2_α_2_, αβαβ, and βααβ [28, 29]. In the crystal structure of compound **1** (Figure 1) **L_1_** adopts the αβα_2_ conformation, in which three pyridyl groups are approximately aligned along the *a*‐axis and the remaining pyridyl group along the *c*‐axis. Adjacent **L_1_** ·4I_2_ units, related by inversion‐symmetry, form interdigitated dimers through their α‐oriented pyridyl rings (Figures 1b and S2). The crystal packing features adjacent dimers in which β‐oriented pyridyl rings engage in weak linear chalcogen bond (ChB) [41] with neighboring TTF cores of the type C─S···I [S7···I1^ii^ = 3.7239(15) Å; C17─S7···I1^ii^ = 178.33(18)°; ^ii^ = −*x*, −1/2+*y*, 1/2−*z*; sum of S and I vdW radii = 3.78 Å] [42] (Figure S2).

### 2.2 Reactions of **L_1_** With Br_2_

By reacting **L_1_** with Br_2_ (1:4 molar ratio) in CHCl_3_/CH_3_CN 1:1 solution, single crystals showing two different morphologies were isolated. Crystallographic analysis established the two compounds as [H_4_ **L_1_**](Br)_2_[Br_3_]_2_·0.7CHCl_3_ (**2**) and [H_3_ **L_1_**](Br)_2_[Br_3_]·(Br_2_)_0.64_·2CHCl_3_ (**3**; Tables S1, S4–S7). Compound **2** crystallized as red plate‐like crystals in the triclinic space group $P\bar{1}$ (Table S1, Figure 2a). The asymmetric unit contains a tetraprotonated ligand [H_4_ **L_1_**]^4+^, two bromide anions (Br3 and Br4), one fully occupied tribromide (Br5─Br6─Br7), and two half‐occupied tribromides (Br1─Br2─Br1^i^), and another disordered over two alternative configurations involving Br9A or Br9B terminal atoms (each with 50% occupancy) around the central Br8 atom (Table 2 and Figure 2).

**FIGURE 2:**
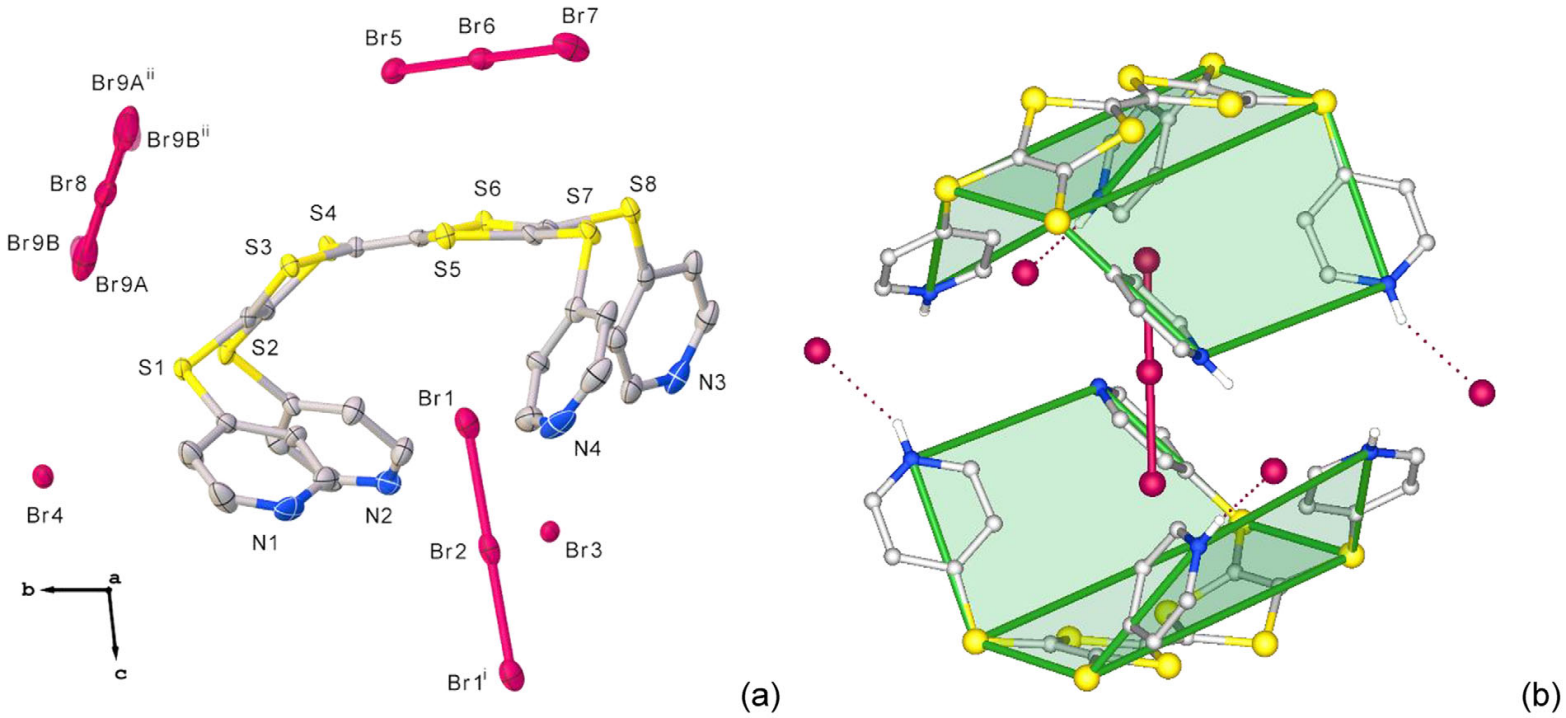
Ellipsoid drawing of compound **2** seen along the [100] direction with the atom labeling scheme for heteroatoms (a); ball and stick representation seen along the *b*‐axis of the supramolecular cage formed by hydrogen‐bonded [H_4_ **L_1_**]^4+^ cations and Br^−^ anions at the [Br_3_]^−^ template (b). Displacement ellipsoids were drawn at 50% probability level. Chloroform molecules and hydrogen atoms not involved in HBs were omitted for clarity. Dotted lines represent N─H···Br HBs. Symmetry codes: ^i^ = 1−*x*, 1−*y*, 1−*z*; ^ii^ = 1−*x*, 2−*y*, −*z*.

**TABLE 2:** Bond distances *d* (Å), angles *α* (°), and relative elongations *δ* for X─Y─X (inter)trihalides in compounds **2–6** [X = Br (**2–4**) and Cl (**5**, **6**); Y = Br (**2**, **4**) and I (**4–6**)].

Compounds	[X─Y─Z]^–^	*d*_X─Y_	*d*_Y─Z_	*α*_X─Y─Z_	*δ*_X─Y_	*δ*_Y─Z_
**2**	Br1─Br2─Br1^i^ ^a^	2.5445(13)	2.5445(13)	180	0.1160	0.1160
	Br5─Br6─Br7	2.5372(15)	2.5451(15)	178.70(3)	0.1128	0.1163
	Br9A─Br8─Br9A^ii^ ^a^	2.518(2)	2.518(2)	180	0.1044	0.1044
	Br9B─Br8─Br9B^ii^ ^a^	2.539(4)	2.539(4)	180	0.1136	0.1136
**3**	Br3─Br4─Br5	2.5572(17)	2.5104(16)	173.13(7)	0.1216	0.1011
**4**	Br1─I1─Br2	2.7241(15)	2.6765(15)	180	0.0940	0.0749
	Br3─I2─Br4	2.7487(11)	2.7095(11)	177.26(4)	0.1040	0.0882
	Br5─I3─Br5^i^ ^b^	2.6823(8)	2.6823(8)	180	0.0921	0.0921
**5**	Cl3─I1─Cl4	2.6162(12)	2.4810(12)	179.27(4)	0.1277	0.0694
	Cl5─I2─Cl5^i^ ^c^	2.5336(15)	2.5336(15)	180	0.0921	0.0921
	Cl6─I3─Cl6^ii^ ^c^	2.5515(11)	2.5515(11)	180	0.0998	0.0998
**6**	Cl1─I1─Cl2	2.5595(19)	2.5291(17)	179.65(5)	0.1032	0.0901
	Cl3─I2─Cl4	2.5282(11)	2.5458(11)	173.55(4)	0.0973	0.0897
	Cl5─I3─Cl6	2.5888(14)	2.5642(16)	177.97(5)	0.1159	0.1053

^a^ ^i^ = 1−*x*, 1−*y*, 1−*z*; ^ii^ = 1−*x*, 2−*y*, −*z*.

^b^ ^i^ = 1–*x*, –*y*, 1–*z*.

^c^ ^i^ = −*x*, 1−*y*, −*z*; ^ii^ = 1 −*x*, −*y*, −*z*.

A chloroform molecule disordered over two positions with equal occupancies is also present. Additional residual electron density, corresponding to approximately 0.7 CHCl_3_ molecules co‐crystallized in the lattice, could not be explicitly modeled and was accounted for using a solvent mask [43].

The tetraprotonated ligand [H_4_ **L_1_**]^4+^ adopts a α_2_α_2_ conformation, with all pyridinium moieties on the same side of the TTF moiety engaging in HBs N─H···Br with neighboring bromides and N···Br distances ranging between 3.169(6) and 3.394(5) Å (Figure S3). Pairs of symmetry related [H_4_ **L_1_**]^4+^ cations include the almost symmetric tribromide Br5─Br6─Br7 (Br5─Br6 and Br6─Br7 distances 2.5372(15) and 2.5451(15) Å, respectively; Br5─Br6─Br7 = 178.70(3)°; Table 2 and Figure 2). The crystal packing features an infinite arrangement of hydrogen bonded boxes, pillared along the *a*‐axis, which are surrounded by half‐occupied tribromides and co‐crystallized solvent molecules (Figure S3).

Compound **3**, [H_3_ **L_1_**](Br)_2_[Br_3_]·(Br_2_)_0.64_·2CHCl_3_, was obtained as orange block‐shaped crystals that crystallized in the monoclinic space group *C*2/*m* (Figure 3a and Tables S1, S6–S7). The asymmetric unit of **3** comprises half of the [H_3_ **L_1_**]^3+^ cation, one tribromide anion (Br3─Br4─Br5), and two bromide anions (Br1/Br1A and Br2) located on mirror planes (Table 2 and Figure 3a). One of the bromides is disordered over two sites (Br1/Br1A) with refined occupancies of 0.60 and 0.40, respectively. The lattice also contains neutral Br_2_ molecules (Br6─Br6^iii^ and Br7─Br7^ii^), which are co‐crystallized and distributed over two partially occupied sites (Figure 3a). Finally, one chloroform molecule is disordered across a mirror plane, while a second chloroform molecule exhibited severe disorder and could not be modeled reliably; its contribution was instead accounted for by applying a solvent mask [43]. The protonated polytopic donor [H_3_ **L_1_**]^3+^ exhibits the same α_2_α_2_ conformation observed for [H_4_ **L_1_**]^4+^ in compound **2**. In compound **3**, supramolecular cages are formed by N─H···Br HBs involving pairs of [H_3_ **L_1_**]^3+^ cations and two bromine molecules. Each cage hosts bromide anions and solvent molecules (Figures 3b and S4).

**FIGURE 3:**
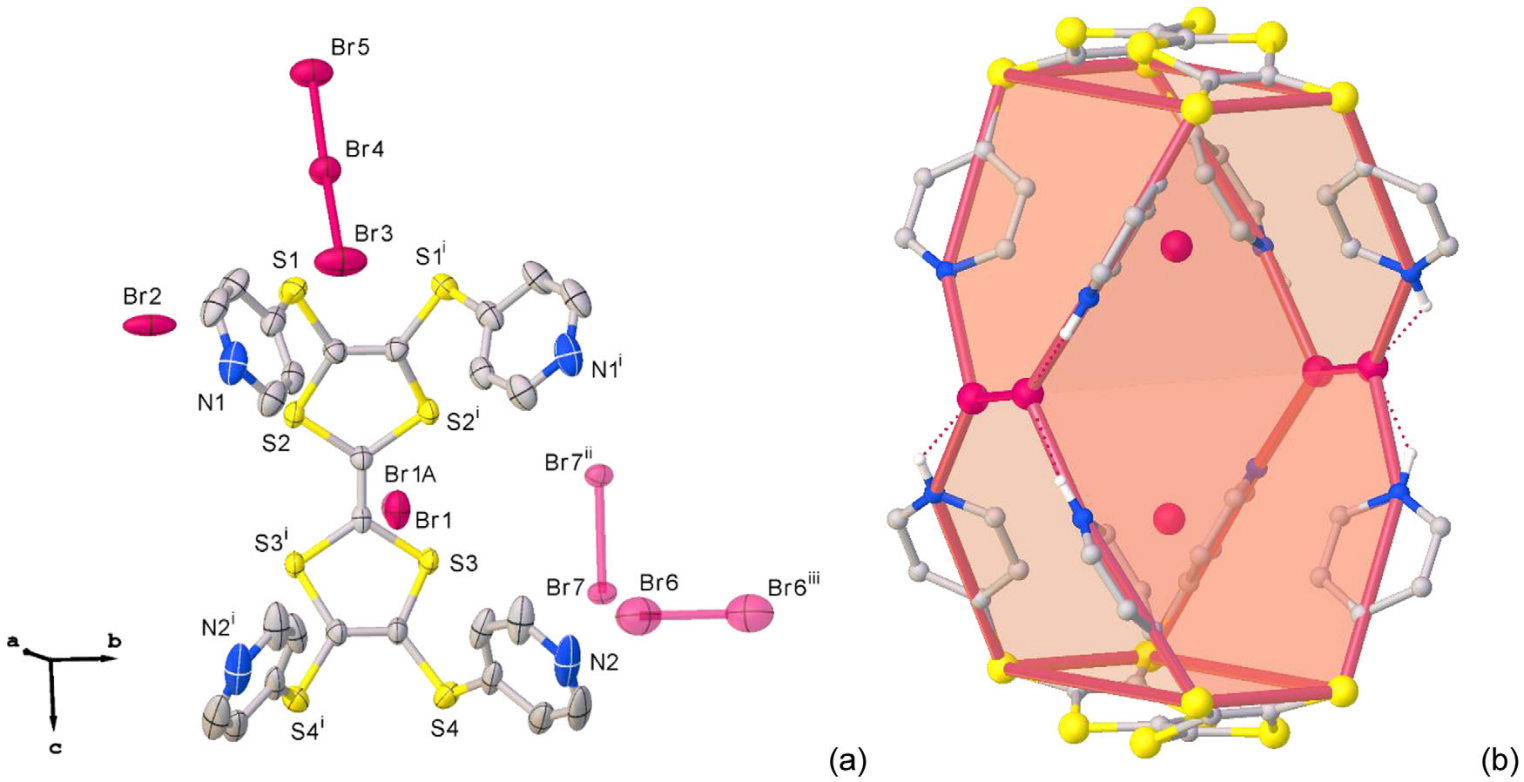
Ellipsoid drawing of compound **3** with the atomic numbering scheme for heteroatoms (a); ball and stick representation of the supramolecular cage {[(H_3_ **L_1_**)_2_(Br_2_)_2_](Br)_2_}^4+^ formed by hydrogen‐bonded [H_3_ **L_1_**]^3+^ cations and bromine molecules (considering the Br7‐containing orientation) and embedded bromides (Br1) (b). Displacement ellipsoids were drawn at 50% probability level. Dotted lines represent N─H···Br HBs. Chloroform molecules and hydrogen atoms not involved in HBs were omitted for clarity. Symmetry codes: ^i^ = +*x*, 1−*y*, +*z*; ^ii^ = 1−*x*, +*y*, 1−*z*; ^iii^ = +*x*, 2−*y*, +*z*.

### 2.3 Reactions of L_1_ With IBr and ICl

Compound **4** was obtained by reacting compound **L_1_** and IBr (1:6 molar ratio) in a CHCl_3_/CH_3_CN mixture. Single‐crystal X‐ray diffraction established compound **4** as [H_4_ **L_1_**](**L_1_**)(IBr_2_)_4_·2CH_3_CN·2.4CHCl_3_ (Tables S1, S8–S9 and Figure 4). In the crystal structure, a fully protonated [H_4_ **L_1_**]^4+^ cation interacts with a neutral molecule of **L_1_** via N─H···N hydrogen bonding interactions [distance N1···N2 = 2.6918(8) Å], giving rise to an inclusion complex consisting of a twisted prismatic cationic cage [(H_4_ **L_1_**)(**L_1_**)]^4+^, in which both components adopt an α_2_α_2_ conformation. This assembly encapsulates a classic [IBr_2_]^−^ anion (Br1─I1─Br2) lying on a twofold screw axis (Figure 4). Since the [(H_4_ **L_1_**)(**L_1_**)]^4+^ cage has not been observed in other structures, this represents, to the best of our knowledge, the first example of a tri(inter)halide templating this structural motif. A related example is provided by a naphthalenediimide‐containing hexacationic cage, which was proved to include [I_3_]^−^ anions [44].

**FIGURE 4:**
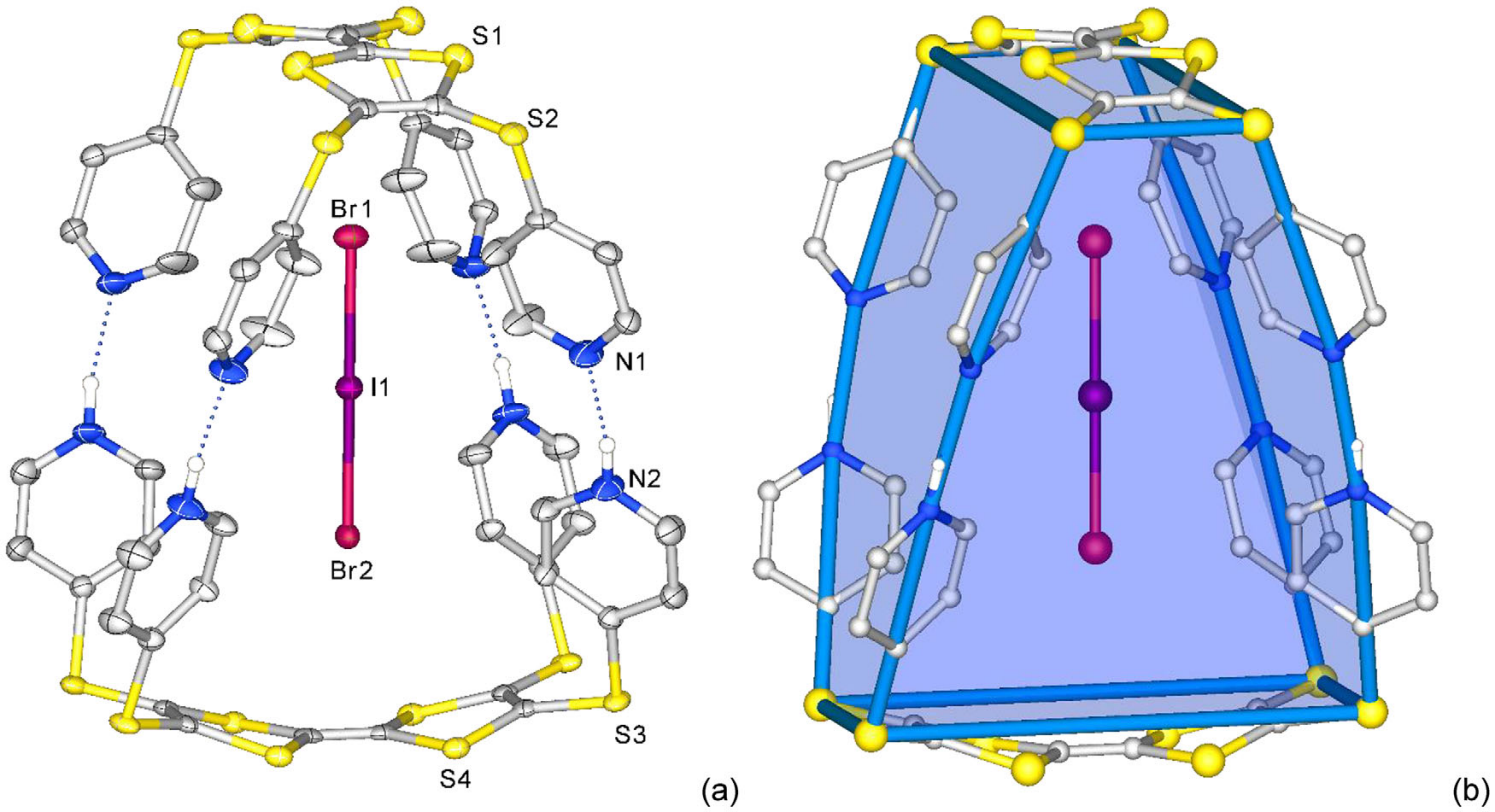
Prismatic motif [(H_4_ **L_1_**)(**L_1_**)]^4+^ embedding a [IBr_2_]^−^ anion in compound **4** with the atomic numbering scheme adopted for heteroatoms only. (a) Displacement ellipsoids were drawn at 50% probability level; only hydrogen atoms of the pyridinium moieties were shown; dotted lines represent N–H···N HBs. (b) Ball and stick representation highlighting the twisted prismatic arrangement.

Two different crystallographically independent slightly asymmetric [IBr_2_]^−^ anions (Br3─I2─Br4 and Br5─I3─Br5^i^, Table 2) are located outside the cationic assembly (Figure S5). The geometrical parameters of these anions are consistent with the average I─Br bond lengths (2.717 Å) reported for linear Br─I─Br moieties in the CSD (Table 2). The lattice additionally includes two acetonitrile molecules and a partially occupied, disordered region accounting to 2.4 co‐crystallized chloroform molecules.

Under conditions analogous to those leading to compound **4**, the reaction of **L_1_** with ICl afforded two distinct ionic products: [H_4_ **L_1_**][ICl_2_]_2_(Cl)_2_·CH_3_CN·2CHCl_3_ (**5**) and [H_3_ **L_1_**][H_4_ **L_1_**][ICl_2_]_3_(Cl)_4_·CHCl_3_·1.5I_2_ (**6**; Tables S1, S10–S13).

The asymmetric unit of compound **5** comprises a tetracation [H_4_ **L_1_**]^4+^, two chlorides (Cl1 and Cl2), a fully occupied [ICl_2_]^−^ (Cl3─I1─Cl4) and two halves of crystallographically independent [ICl_2_]^−^ anions (Cl5─I2─Cl5^i^ and Cl6─I3─Cl6^ii^; Figure 5, Table  2). The protonated N‐donor adopts an α_2_α_2_ conformation. N─H···Cl HBs between the pyridinium cations and chloride anions [N···Cl distances ranging between 2.995(4) and 3.050(4) Å] generate one‐dimensional (1D) ribbons propagating along the *a*‐axis. Notably, the cations within these ribbons are slightly offset relative to each other (Figure S6). Pairs of symmetry‐related [H_4_ **L_1_**]^4+^ cations engage in hydrogen bonding with six chloride anions to define a distorted prismatic cage, which hosts two classical [ICl_2_]^−^ anions (Figure 5b).

**FIGURE 5:**
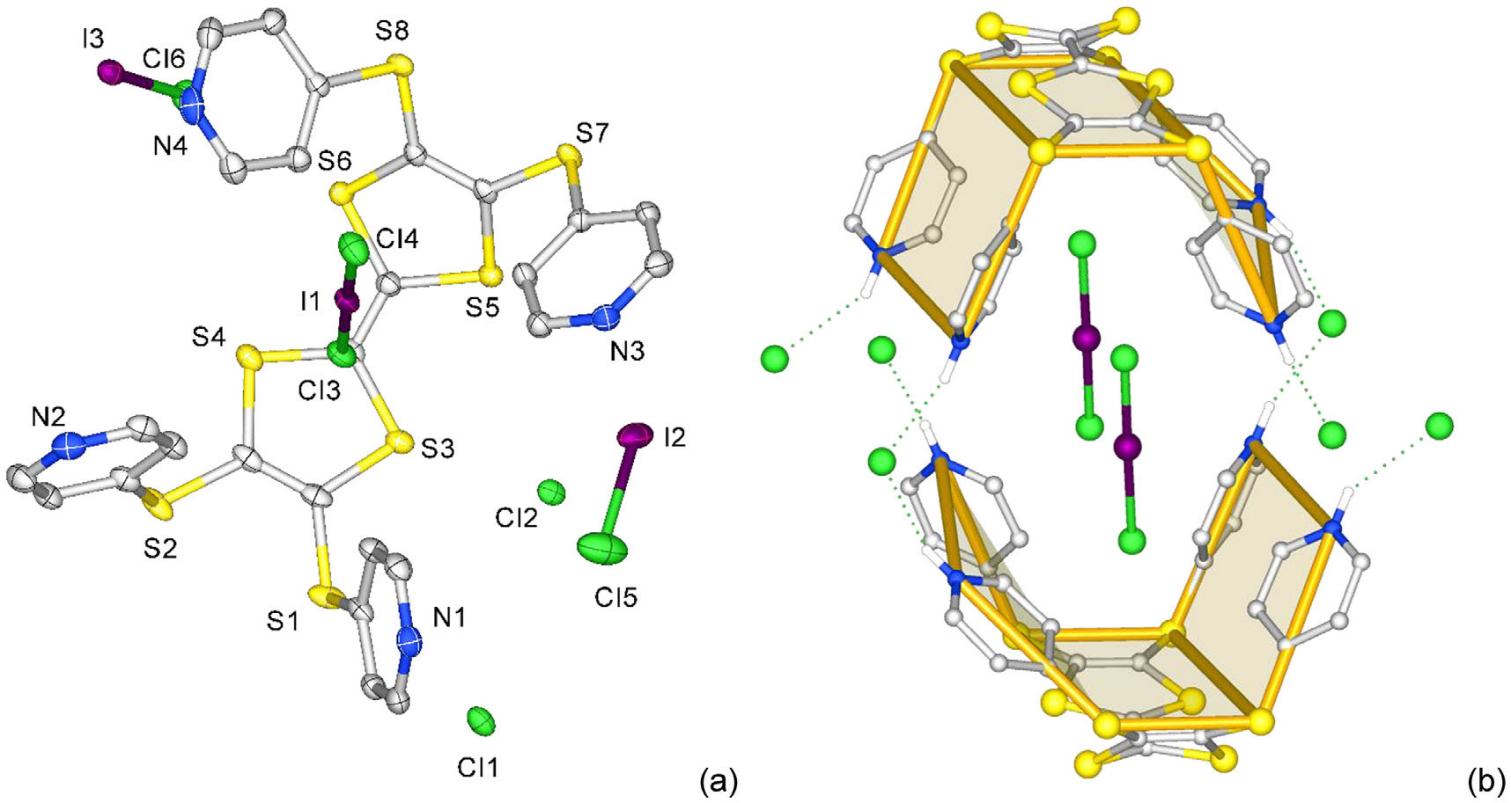
Ellipsoid drawing of the asymmetric unit of compound **5**, viewed along the *c*‐axis, with the atomic numbering scheme adopted for heteroatoms only (a); ball and stick representation of the prismatic cage formed by pairs of hydrogen‐bonded [H_4_ **L_1_**]^4+^ cations and Cl^−^ anions at the [ICl_2_]^−^ template (b). Dotted lines represent N─H···Cl HBs. Thermal ellipsoids were drawn at 50% probability level. Solvent molecules and hydrogen atoms not involved in HBs were omitted for clarity.

The crystal structure of the ionic compound **6** contains two differently protonated donors, [H_4_ **L_1_**]^4+^ and [H_3_ **L_1_**]^3+^, a linear [ICl_2_]^−^ anion (Cl1─I1─Cl2), along with two additional [ICl_2_]^−^ anions (Cl3─I2─Cl4 and Cl5─I3─Cl6) and four chloride anions (Cl7A/Cl7B, Cl8, Cl9, and Cl10) (Figure 6). Both protonated N‐donors adopt a α_2_α_2_ conformation. Similar to compound **5**, several N─H···Cl HBs generate 1D ribbons, in which the cations are aligned (in contrast to the slight offset observed in **5**), propagating along the *a*‐axis (Figure S7). A chloroform molecule could not be modeled, and a region of severely disordered I_2_ molecules occupies the space between the ribbons. Analogously to compound **2–5**, in compound **6** the [ICl_2_]^−^ anion (Cl1─I1─Cl2) templates a prismatic cage formed by pairs of [H_4_ **L_1_**]^4+^/[H_3_ **L_1_**]^3+^ cations (Figure 6b).

**FIGURE 6:**
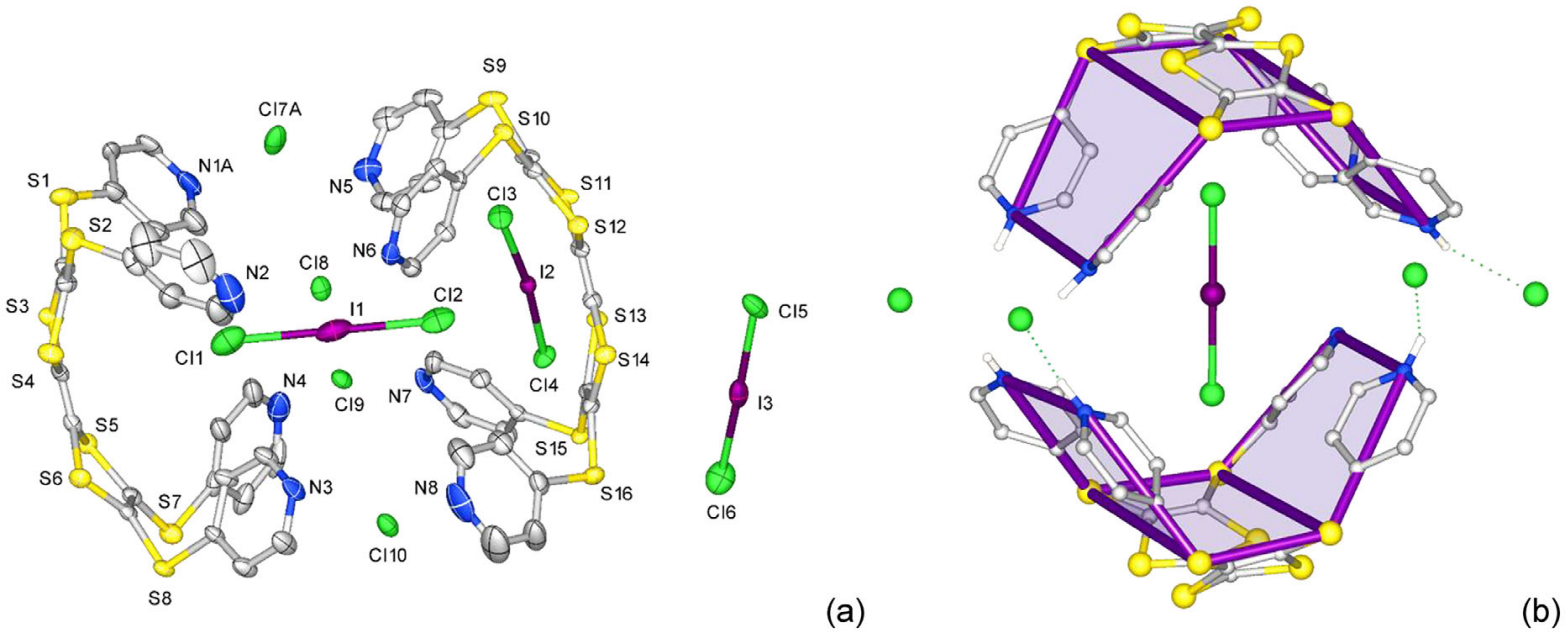
The crystal structure of compound **6** seen along the *a*‐axis with the atomic labeling scheme for heteroatoms (a) and prismatic cage formed by [H_4_ **L_1_**]^4+^/[H_3_ **L_1_**]^3+^ cations including a [ICl_2_]^−^ anion (b). Dotted lines represent N─H···Cl HBs. Thermal ellipsoids were drawn at 50% probability level. Solvent molecules and hydrogen atoms were omitted for clarity. Disorder at one pyridyl ring (Py_N1A_/Py_N1B_) and one chloride (Cl7A/Cl7B) was not shown.

### 2.4 Structural Correlations

All compounds **1–6** contain one or two units of the ligand [H*_x_* **L_1_**]*^x^* ^+^ variously protonated (4 ≥ *x* ≥ 0). Since protonation in all cases involves the pyridine pendants, no significant variations in the metric parameter of the TTF core were observed (Table S14). Regardless of the degree of protonation of **L_1_**, the formation of a supramolecular dimeric, cage‐like assembly emerges as a recurring motif in the ionic products. In this arrangement, pairs of interacting α_2_α_2_ conformers of protonated **L_1_** accommodate a variety of templating anionic species, including tribromides and bromides (compounds **2** and **3**), [IBr_2_]^−^ (compound **4**), and chloride and [ICl_2_]^−^ anions (compounds **5** and **6**).

Some of the authors reported an extended investigation on structural data retrieved from the CSD, revealing that apparently uncorrelated linear three‐body systems containing either halogen atoms, X─X─X (X = Br and I), or combinations of halogen(s) and chalcogen(s) atoms, Ch─X─Y, X─Ch─Y, and Ch─X─Ch (Ch = S, Se; X = Y = Cl, Br, I; X = I, Y = Cl, Br), as well as purely chalcogen‐based species [45] such as Se─Se─Se, share a common structural correlation [46]. In fact, the normalized elongations (*δ*_A─B_ and *δ*_B─C_) of the two bonds, relative to the sum of the relevant atomic radii can be calculated as:

(1) $$\;\delta_{A-B}=\frac{d_{A-B}-\left(r_{A}+r_{B}\right)}{r_{A}+r_{B}}\;;\;\delta_{B-C}=\frac{d_{B-C}-\left(r_{B}+r_{C}\right)}{r_{B}+r_{C}}\;$$

where *d*_A–B_ and *d*_B–C_ are the structural distances within the A─B─C system, and *r*_A_, *r*_B_, and *r*_C_ the covalent radii of A, B, and C atoms. The correlation between *δ*_A–B_ and *δ*_B–C_ can be fitted by a nonlinear equation derived from the bond‐valence model [47]:

(2) $$\;\delta_{A-B}=-k\cdot ln\left[1-e^{-\frac{\delta_{B-C}}{k}}\right]$$

where *k* ≈ 0.160 is an empirical fitting constant. In the resulting plots, the central part corresponds to strongly correlated *d*_A─B_ and *d*_B─C_ distances, typical of largely covalent three‐body systems, such as symmetric or slightly asymmetric trihalides [X_3_]^−^ and tri(inter)halides [XY_2_]^−^. The correlated bond order in these species can be described according to the 3c–4e molecular orbital (MO) model [46]. In contrast, data located in the peripheral region of the plots, aligned roughly parallel to the Cartesian axes, are poorly correlated. This indicates that in these systems the interactions cannot be interpreted in terms of the MO CT or 3c–4e models (i.e. orbital mixing). Instead, noncovalent electrostatic and/or dispersion interactions, typical of HaB and ChB [41, 48, 49], prevail. It is worth noting that since all *δ*_A─B_/*δ*_B─C_ pairs can be fitted with a single equation, no net separation exists between covalently bonded three‐body systems and systems interacting by electrostatic/dispersion NCIs.

This correlation can be successfully extended to the structural data containing the N─X─Y fragment (XY = Br_2_, I_2_, ICl, and IBr; Figure S8) [9]. Also in this case, the central region of the plot comprises strongly covalently bonded systems (i.e. authentic CT adducts), while data points aligned approximately parallel to the Cartesian axes correspond to systems with limited CT, and thus featuring predominantly electrostatic N···X─Y interactions. The largest *δ*_X─Y_ values, indicating a greater relative perturbation of the X─Y fragment, are observed for N─I─Cl and N─I─Br fragments, while N─I─I and the very few examples of N─Br─Br fragments show the smallest variations in the interhalogen distance.

The four distinct N─I─I moieties in compound **1** nicely fit the *δ*_N─X_ versus *δ*_X─Y_ correlation when compared with all compounds featuring a N─X─Y group (Figure S8), and feature large *δ*_I─I_ values (Table 1) among the “spoke” adducts between nitrogen donors and diiodine deposited at the CSD (Figure S8). The corresponding data fall in the central region of the plot, corresponding to the highest degree of correlation between *δ*_N─I_ and *δ*_I─I_ relative elongations. This suggest that the N···I─I moieties in compound **1** display a significant orbital mixing contribution to the interaction, supporting a description in terms of at least partial CT. The same correlation can be applied to the tri(inter)halides in compounds **2–6** (Table 2), which closely follow the correlation previously described for both neutral and charged trihalogen systems, including Cl─Cl─Cl, Br─Br─Br, I─I─I, I─Cl─I, and I─Br─I fragments (Figure S9). The fitting constant for the N─I─I and X─Y─X correlations (*k* = 0.153 and 0.152, respectively), are extremely close. Indeed, N─I─I systems can be described within a unified correlation that also encompasses [X_3_] and [X_2_Y] trihalogen systems (X = Cl, Br, and I; Y = I; Figure 7). Hence, the observed similarity in structural features across a wide range of systems, encompassing symmetric and asymmetric trihalogen, tri(inter)halogen, hypercoordinate chalcogen adducts, Ch‐ and N‐bonded CT adducts, and three‐chalcogen sequences [46], strongly supports a common nature of the chemical bonding in all these species.

**FIGURE 7:**
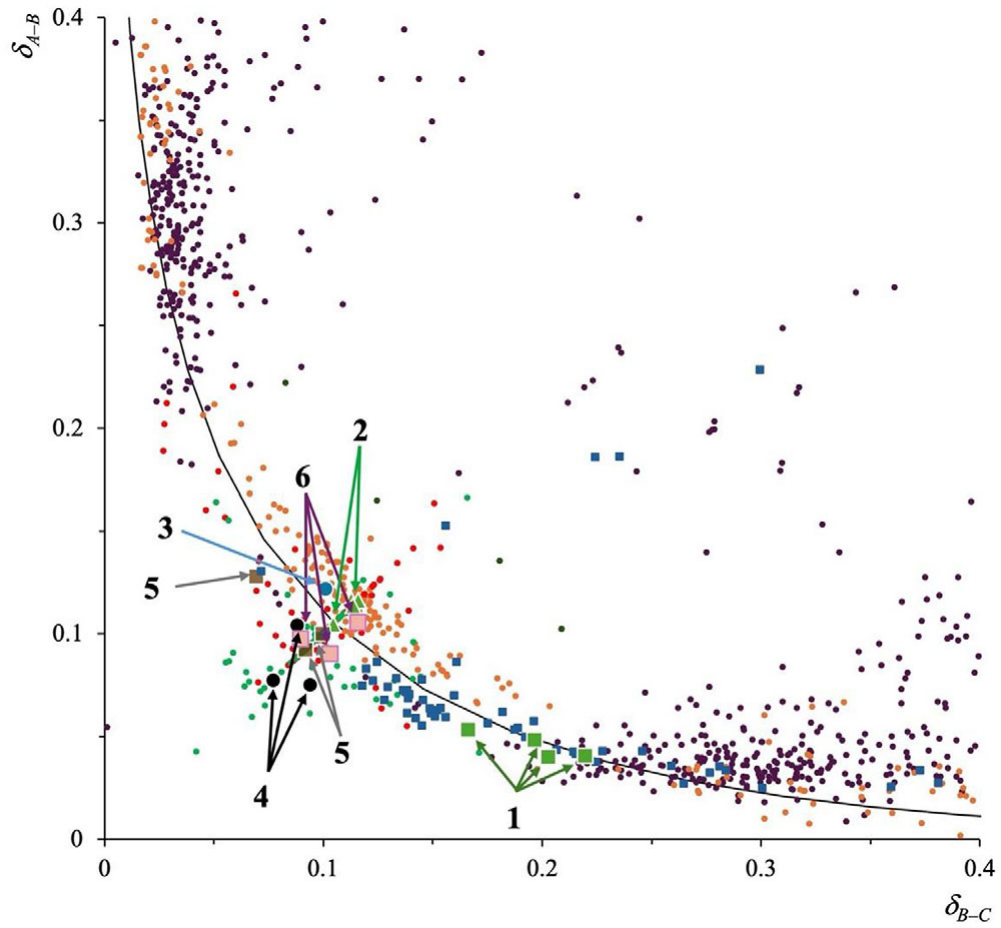
Structural data of the N─I─I fragments in compound **1**, three‐halogen sequences X─Y─X in compounds **2–6** and N─X─Y deposited at the CSD and X─Y─X sequences considered previously (see ref. 46) as scatter plot of *δ*_X─Y_ versus *δ*_Y─X_ (Equation 1). The solid curve represents the least‐squares fit of the data adopting Equation 2 as a model. CSD data: Purple dots, I─I─I; orange dots, Br─Br─Br; dark green dots, Cl─Cl─Cl; light green dots, Cl─I─Cl; red dots, Br─I─Br; cyan squares: N···X─Y (X = I, Br; Y = Cl, Br, I). Structural data discussed in this work: green squares, N─I─I in compound **1**; green triangles, [Br_3_]^−^ in compound **2**; cyan circles, [Br_3_]^−^ in compound **3**; black circles, [IBr_2_]^−^ in compound **4**; brown squares, [ICl_2_]^−^ in compound **5**; pink squares, [ICl_2_]^−^ in compound **6**.

### 2.5 Vibrational Spectroscopy

All compounds were characterized by FT–IR and Raman spectroscopy (Figures 8 and S10–S16). In particular, Raman spectroscopy in the low‐energy region provides structural information on dihalogens, interhalogens, and poly(inter)halides [5, 50, 51]. In “spoke” I_2_‐adducts, the elongation of perturbed diiodine is reflected in the shift of the Raman peak due to the I─I σ_g_ stretching vibration [11]. Molecular iodine (bond order *n*_I─I_ = 1) exhibits in the gas phase a characteristic Raman peak at 214.5 cm^−1^, corresponding to the I─I stretching vibrational mode [52, 53, 54, 55]. The same vibration is observed in the range 203–211 cm^−1^ in low‐polar organic solvents [55]. Accordingly, nearly monomeric I_2_ trapped in solid Kr at 32 K displays a distinct Raman peak at 210.8 cm^−1^ [54]. The unit cell of crystalline I_2_ contains two molecular units, and two peaks can be observed, assigned to the in‐phase (A_g_, 180 cm^−1^) and out‐of‐phase (B_3g_, 189 cm^−1^) stretching modes [56]. At room temperature, the Raman spectra of solid diiodine displays only the former peak [55, 57]. In systems containing perturbed diiodine (*n*_I─I_ < 1), such as CT adducts, the weakening of the I─I bond is reflected in a roughly linear shift of the Raman A_g_ peak toward lower wavenumbers [58]. In linear symmetric triiodides, showing I─I distances in the range 2.90–3.00 Å (*n* = 0.5), due to the presence of an inversion centre, the σ_g_ symmetric stretching vibration can be observed in the Raman spectrum at about 110 cm^−1^ (ν_symm_), while the antisymmetric σ_u_ stretching mode (ν_asymm_) and the π_u_ bending mode (ν_b_) are only IR‐active. In asymmetric triiodides (I─I distances in the range 2.80–3.10 Å; *n* = 0.4–0.6), lacking of an inversion center, the antisymmetric stretching vibration σ_u_ and the bending modes are also Raman‐active, and they are typically observed at about ν_asymm_ = 140 and ν_b_ = 70 cm^−1^ [26]. Strongly asymmetric triiodides [I···I–I]^−^ can be regarded as weak adducts: a single peak in the range 140–180 cm^−1^ is clearly visible, while the peak corresponding to ν_symm_ is very weak or absent. The Raman spectra of trichlorides and tribromides can be interpreted analogously. In particular, the Raman spectra of [Cl_3_]^−^ and [Br_3_]^−^ were reported to feature ν_symm_ at about 270 [59] and 160 cm^−1^ [7, 50, 60, 61], respectively. Classic tri(inter)halides, such as [IBr_2_]^−^ and [ICl_2_]^−^, show the typical symmetric stretching vibration ν_symm_ at wavenumbers close to those of [Br_3_]^−^ and [Cl_3_]^−^, that is, at about 160 and 260 cm^−1^, respectively [7, 50].

**FIGURE 8:**
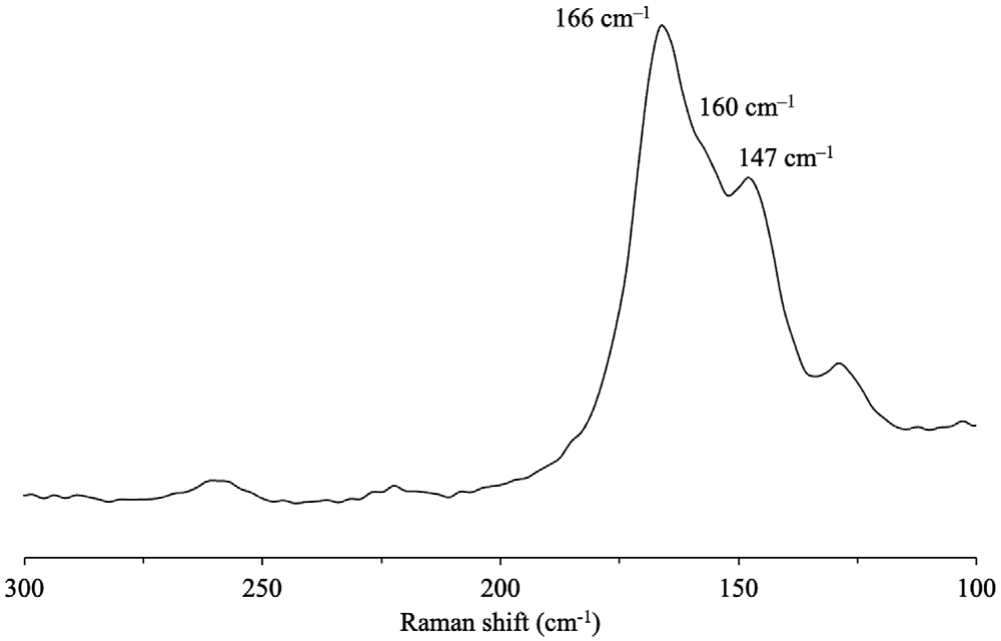
Solid state Raman spectrum (100–300 cm^−1^) recorded at r.t. for compound **4**.

Raman spectroscopy measurements were carried out on crystals of compounds **1–6**. Only in the case of compounds **1**, **4**, and **5** the spectra allowed identification of the peaks of Raman‐active species, whereas the spectra of compounds **2**, **3,** and **6** were overwhelmed by the fluorescence emission from the analyzed samples. The Raman spectrum of compound **1** shows a broad band centered at 155 cm^−1^ (Figure S10, top). This structured band can be attributed to the overlap of peaks at slightly different wavenumbers due to the stretching vibration of the four diiodine moieties, perturbed as a result of the CT interaction (Figure 1 and Table 1). The shift of this peak (about 25 cm^−1^) with respect to that observed for I_2_ in the solid state is consistent with the moderate elongation of the I─I bond (see above) and classifies compound **1** as a weak CT adduct (bond order *n*_I─I_ > 0.6) [50]. Accordingly, the Raman shift recorded for compound **1** falls within the linear correlation between the measured Raman wavenumber and the I─I bond distances reported for weak I_2_ CT adducts [63].

The Raman spectrum of compound **4** shows a complex overlap of peaks in the range 130–180 cm^−1^ (Figure 8). Three maxima can be distinguished, namely a well‐defined peak at 166 cm^−1^, a shoulder at 160 cm^−1^, and a less intense peak at 147 cm^−1^, which can be tentatively attributed to the stretching modes of the differently elongated Br5─I3─Br5^i^, Br1─I1─Br2, and Br3─I2─Br4 anions. Finally, the Raman spectra of compounds **5** (Figure S10, bottom) displays a broad band centered at 263 cm^−1^, assigned to the overlap of the symmetric vibrations of the slightly differently elongated [ICl_2_]^−^ anions (Table 2).

### 2.6 Theoretical Calculations

The formation of a Lewis adducts between an N‐donor (D) and an XY acceptor (X = Y = I, Br; X = I, Y = Br, Cl) can be described by means of a simplified MO scheme [62, 63]. The interaction leads to a partial CT from the highest occupied molecular orbital (HOMO) of the donor and the σ* antibonding lowest unoccupied molecular orbital (LUMO) of the XY molecule, thereby decreasing the X─Y bond order. The extent of the donor–acceptor interaction is directly correlated with the CT. Stronger interactions result in a greater population of the σ* orbital, a more pronounced reduction in X─Y bond order, and a corresponding increase in the X─Y bond length. The elongation of the X─Y bond can be systematically modulated by varying the donor strength through changes in the chemical environment of the N atom, thus affecting the energy of the HOMO and, consequently, its overlap with the LUMO of the XY molecule. Analogously, the nature of the XY molecule influences the eigenvalues of the frontier orbitals, reflected in the different oxidation ability of the considered dihalogens/interhalogens.

To analyze the donor‐acceptor interactions in compound **1**, the donor **L_1_**, diiodine, and the relevant adduct **L_1_** ·4I_2_ were investigated at the DFT level of theory, with the hybrid mPW1PW [64] functional paralleled by triple‐ζ split‐valence basis sets including polarization functions in the formulation of Weigend [65] for all atoms except halogens, for which the LANL08(d) [66] basis set including polarization and diffuse function with relativistic effective core potentials (RECPs) was selected (see Experimental Section).

The possible conformers of **L_1_** are extremely close in energy, differing by about 1.4 kcal·mol^−1^ in their total electronic energy. The frontier Kohn–Sham MOs (KS‐MOs; HOMO #164 and LUMO #165 for the α_2_α_2_ conformer) calculated for **L_1_** at the optimized geometry are π–in–nature and localized on the TTF core. The nonbonding KS‐MOs #156–159 are mainly contributed by the four lone‐pairs (LPs) of electrons localized on the negatively charged nitrogen atoms (natural charge *Q*_N_ = −0.407 |e|). These MOs interact with the virtual LUMOs of the four I_2_ units, to give four bonding KS‐MOs (#175–178) and four unoccupied virtual KS‐MOs in **L_1_** ·4I_2_ (#194–197; Figure 9 for the α_2_α_2_ conformer), in agreement with the MO interaction model previously described [63].

**FIGURE 9:**
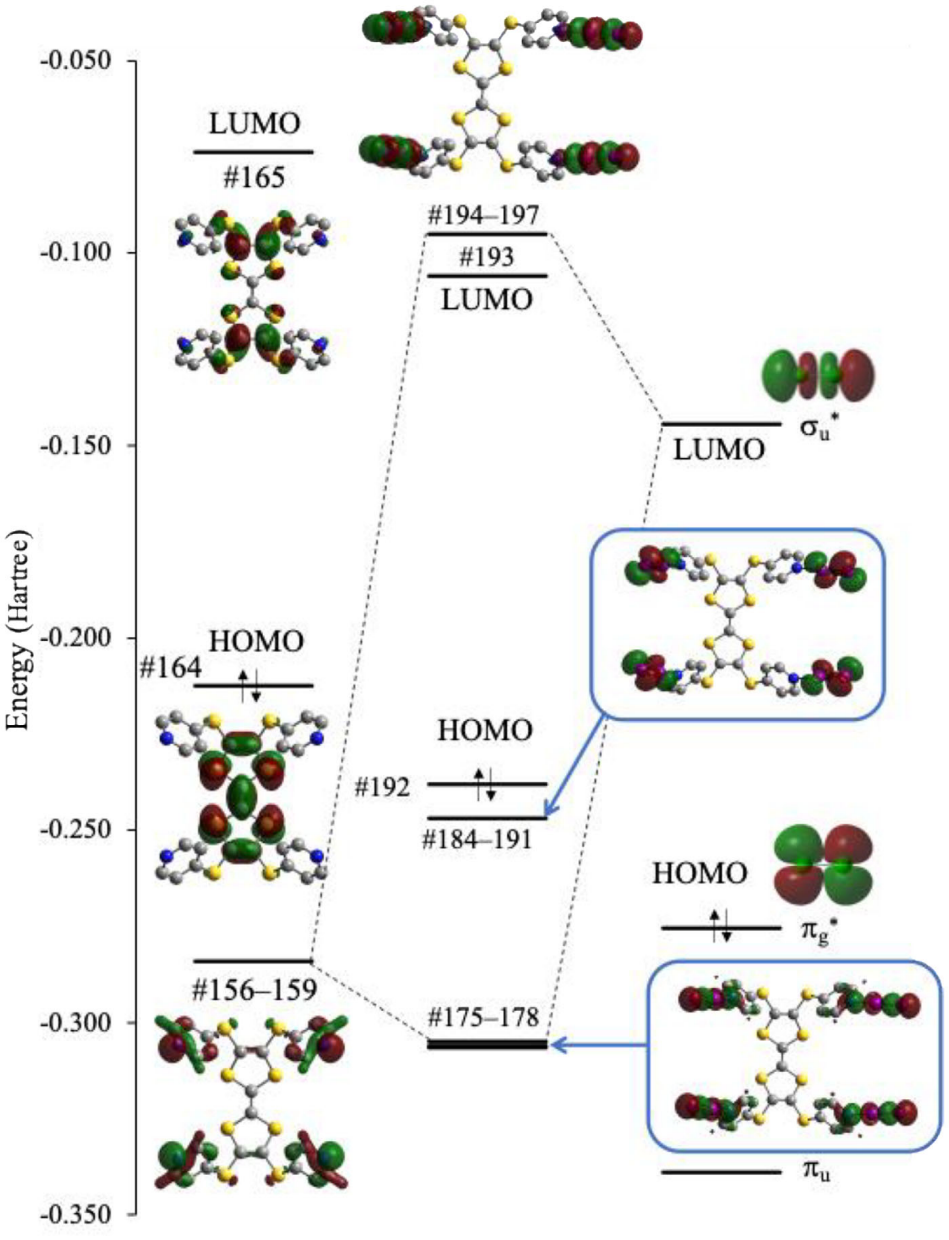
Simplified MO diagram showing the interaction between the donor **L_1_** (α_2_α_2_ conformation) and I_2_ calculated at hybrid DFT level (functional mPW1PW/Def2TZVP (C, H, N, S)/LANL08(d) (I)). Only electron pairs on the HOMOs have been depicted for clarity. Cutoff value for KS‐MO isosurfaces = 0.03 |e|.

The optimization of the adduct **L_1_** ·4I_2_, carried out starting from structural metric parameters, converged to the α_2_α_2_ conformer. The metric parameters of the donor in the optimized adduct **L_1_** ·4I_2_ are in very good agreement with the corresponding structural ones in compound **1** (Table S14). As expected, the adduct formation, which is calculated to be slightly exergonic (Table S15), induces an elongation in the I─I bond distance of 2.3% in **L_1_** ·4I_2_ with respect to that optimized for the corresponding free I_2_ species in the gas phase, indicating a weak interaction between **L_1_** and the four I_2_ units, in agreement with the structural data discussed above. Accordingly, the optimized N─I distance of **L_1_** ·4I_2_ in the gas phase, longer than the corresponding structural one in compound **L_1_** ·4I_2_ by about 0.19 Å (Table S15), suggests that the gas‐phase calculation slightly underestimated the N···I─I interaction compared to the solid state. The CT between the pyridine donor groups and the I_2_ units in **L_1_** ·4I_2_ was calculated by Hirshfeld population analysis [67] to amount to 0.231 |e| for each N···I_2_ moiety, in agreement with the values obtained with NBO 3.1 (0.189 |e|; Table S15) [68, 69], whose reliability has been very recently questioned [70] as compared to the recent NBO 7 implementation. A net CT from **L_1_** to I_2_ units is confirmed by the charges calculated according to Bader partitioning scheme, with a CT estimated in 0.100 |e| (Table S15). All the considered partitioning schemes show that the interaction induces a net polarization in the diiodine units, so that the terminal halogen atoms of the N···I─I fragments show a negative charge, in agreement with a N···I^δ+^─I^δ−^ description (Δ*Q*_I_ = 0.149, 0.189, and 0.258 |e| according to Hirshfeld, NBO, and Bader partitioning schemes, respectively; Table S15). This is reflected in the bond orders of the N···I and I─I bonds. In fact, a modest Mayer bond order [71] MBO_N···I_ = 0.19 was calculated for the N···I interaction while the I─I bond was calculated to be weakened to MBO_I─I_ = 0.84. The decrease in the I─I bond order is correlated to the N···I bond order (MBO_N···I_ + MBO_I─I_ ≈ 1), supporting the description of the N···I─I moiety as an unbalanced three‐body system, in good agreement with the structural correlation discussed above.

Alternative to the MO mixing interpretation, the formation of “spoke” adducts has been considered as a noncovalent halogen bond (HaB) interaction. HaB is defined as a net attractive interaction RX···A between an HaB donor (R = heteroatom, metal ion, organic group, or a second X species) and an acceptor A [41, 48, 72]. HaB, along with ChB [49, 73], pnictogen bond (PnB) [74, 75], and tetrel bond [76], belongs to the σ‐hole family of intermolecular interactions [77, 78, 79], which are governed by the anisotropy of the electrostatic potential at the interacting atoms, the depletion of which (σ‐hole), indicating an electrophilic region of positive potential on the X atom, is typically located opposite to the covalent R─X bond of the donor group. The noncovalent definition of σ‐hole interactions has been criticized [80, 81], although cases of purely electrostatic HaBs have been reported [82]. Indeed, energy decomposition analyses (EDAs) have shown that the energy of σ‐hole interactions can be decomposed into electrostatic term and MO‐mixing terms [81, 83], and that dispersion effects [84] also play an often fundamental role [73, 85, 86]. The relative contribution of each term largely depends on the nature of the involved interaction groups, so that the nature of σ‐hole interactions ranges from almost purely ionic to largely covalent.

The electrostatic potential surface on the atoms of diiodine clearly shows charge depletion regions (σ‐holes), located opposite to the I─I bond (Figure 10, right), that orient the dihalogen units toward the more nucleophilic sites in **L_1_**, that is, those displaying a more negative electrostatic potential, corresponding to the N‐atoms (Figure 10, left). In this context, the “spoke” adduct **1** can be considered as the result of a HaB interaction between the I_2_ HaB donors and the **L_1_** acceptor.

**FIGURE 10:**
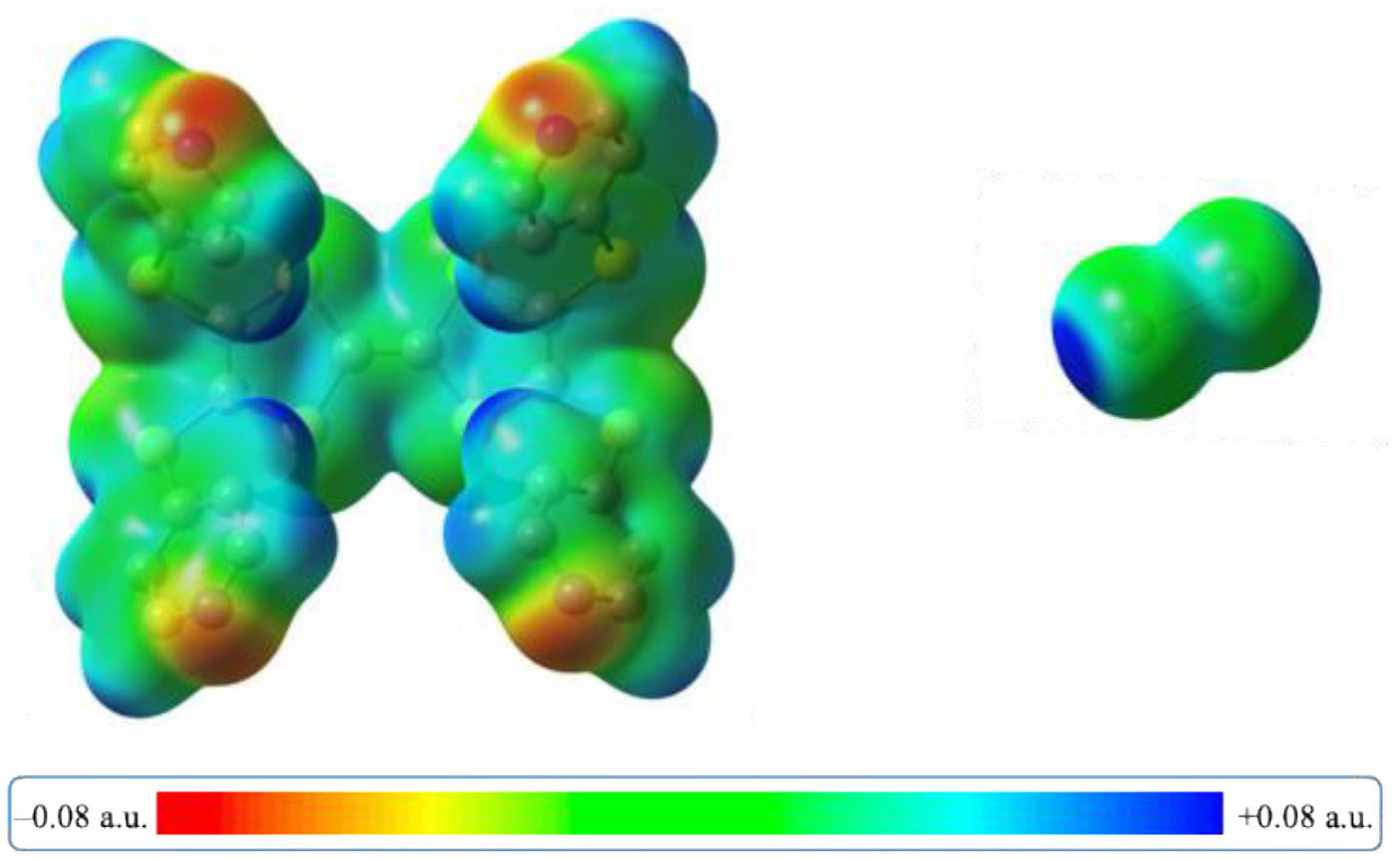
Molecular electrostatic potential (MEP) mapped on the electron density surface (5·10^−3^ |e|·Bohr^–^^3^) in **L_1_** (left) and I_2_ (right). MEP range –0.08 (red)−+0.08 (blue) a.u. calculated at DFT level (functional mPW1PW/Def2TZVP (C, H, N, S)/LANL08(d) (I)). Hydrogen atoms were omitted for clarity.

Noncovalent interactions can be analyzed using the NCI method in the reduced density gradient (RDG) approach [87, 88]. These interactions are identified as low‐gradient isosurfaces in regions of low electron density. The nature of the interaction is determined by the sign of the second largest Hessian eigenvalue of the electron density (*λ*_2_), while its strength is related to the electron density value on the NCI surface. The color‐filled RDG isosurface clearly reveals the presence of an interaction between the nitrogen atoms of the **L_1_** and the diiodine units, which can be classified as a weak halogen bond (*ρ* > 0, sign(*λ*_2_) < 0; Figure 11).

**FIGURE 11:**
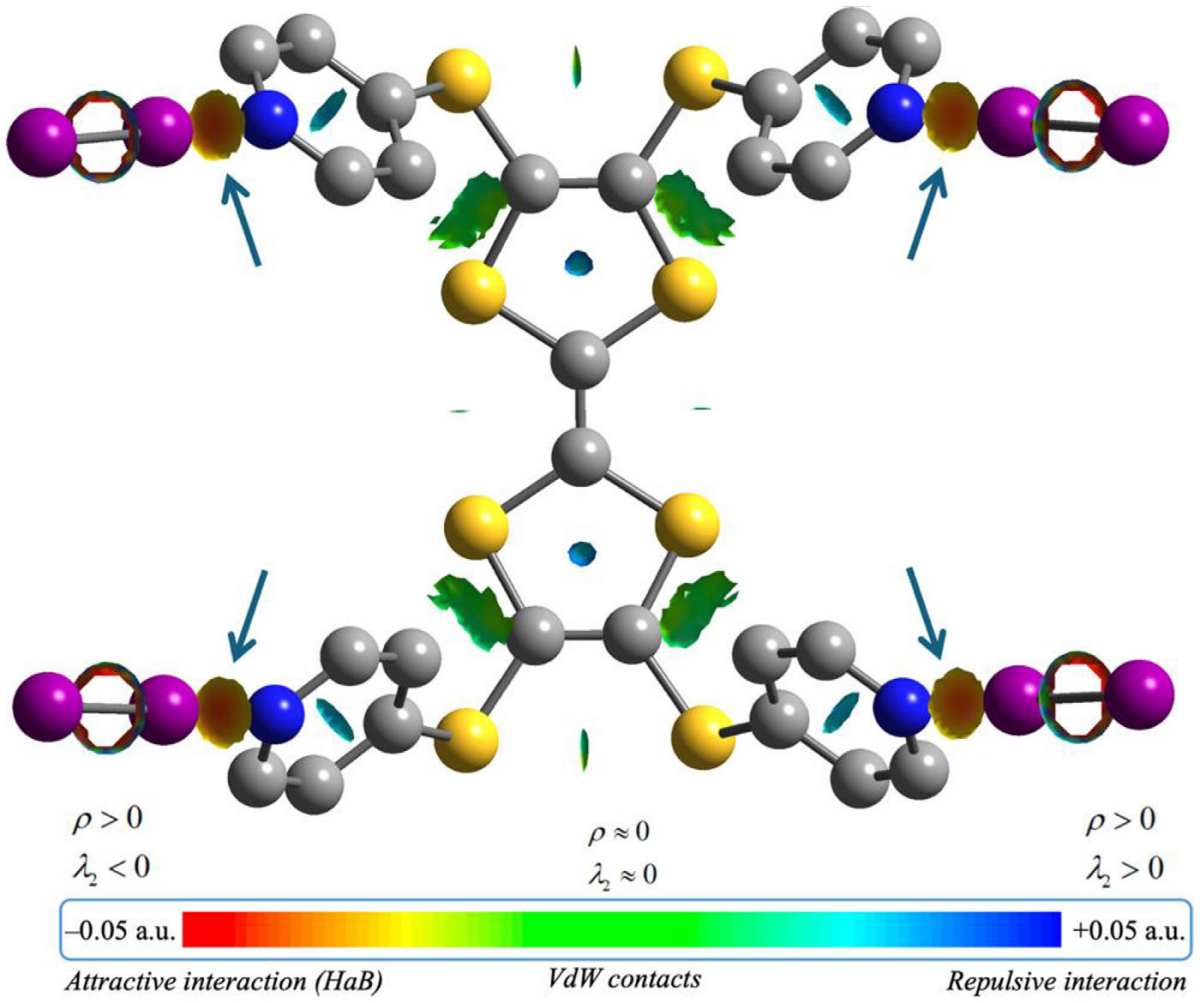
Color‐filled RDG isosurface calculated for compound **1** at DFT level (functional mPW1PW/Def2TZVP (C, H, N, S)/LANL08(d) (I)). Hydrogen atoms were omitted for clarity. Arrows point to HaB interactions (*ρ*·sign(*λ*_2_) < 0). Nonbonded vdW interaction regions are evidenced as green surfaces.

To evaluate the relative weight of the orbital‐mixing and electrostatic terms, an energy decomposition analysis (EDA) based on dispersion‐corrected DFT was carried out according to the sobEDAw [89] energy decomposition method for weak interactions. By considering five interacting fragments (**L_1_** and four I_2_ units), the total interaction energy (Δ*E*_int_) was partitioned into electrostatic (Δ*E*_els_), exchange‐repulsion (Δ*E*_xrep_), orbital interaction (Δ*E*_orb_), and dispersion (Δ*E*_disp_) terms. The total interaction energy Δ*E*_int_, corresponding to four pyridine–I_2_ interactions, amounts to −41.15 kcal·mol^−1^ (−10.28 kcal·mol^−1^ for each weak N···I─I interaction). The balance between Δ*E*_els_ and Δ*E*_orb_ (−102.17 and −74.37 kcal·mol^−1^, respectively), accounting for 48.8% and 35.5% of the total attractive contribution to Δ*E*_int_, clearly shows that both type of interactions contributes to the adduct formation and that no single model of interpretation is sufficient alone.

A further insight into the nature of the interaction comes from QTAIM calculations [90], which revealed two (3, –1) bond critical points (BCPs) for each N···I─I moiety of compound **L_1_** ·4I_2_, corresponding to the N···I interaction and the I─I bond, respectively (Table 3). The nature of BCPs can be evaluated by comparing the sign of the total energy density *H*_BCP_ and of the Laplacian of electron density ∇^2^ *r*_BCP_ at each BCP [91], since weak closed‐shell interactions show both *H*_BCP_ and ∇^2^ *ρ*_BCP_ with a positive sign, whereas covalent interactions show negative *H*_BCP_ and positive ∇^2^ *ρ*_BCP_ values [92]. The BCP located on the bond path between the donor N‐atom and the closest halogen atom shows a negative *H*_BCP_ value and a positive Laplacian ∇^2^ *ρ*_BCP_ (Table 3), testifying a partially covalent interaction of weak strength, confirmed by the ratio |*V*_BCP_|/*G*_BCP_, larger than unity [93]. As expected, the BCP located in between the two iodine atoms features a decrease in the electron density (Δ*ρ*_BCP_) compared to that calculated for the isolated I_2_ molecule (*ρ*_BCP_ = 7.13·10^−2^ a.u. at the same theoretical level). The electron localization function [94] calculated at the BCPs between the coordinated diiodine unit (ELF_BCP_) shows a value larger than 0.5, in agreement with a polarized covalent nature of the I─I bond (ELF_BCP_ for free I_2_ = 0.734), and of 0.217 for the BCP located between the nitrogen donor atom and the closest iodine atom, compatible with a weak HaB interaction.

**TABLE 3:** Summary of (3, –1) BCP calculated for compound **1** in the gas phase at DFT/QTAIM level: Potential electron energy density *V*_BCP_ (x 10^2^), Lagrangian kinetic energy density *G*_BCP_ (x 10^2^), total electron energy density *H*_BCP_ (x 10^2^) density of all electrons *ρ*_BCP_ (x 10^2^), difference in electron density Δ*ρ*_BCP_ (x 10^2^) with respect to the free I_2_ unit, Laplacian of the electron density ∇^2^ *ρ*_BCP_ (x 10^2^), and electron localization functions (ELF_BCP_). All QTAIM parameters are given in atomic units.

BCP	*V*_BCP_	*G*_BCP_	|*V*_BCP_|/*G*_BCP_	*H*_BCP_	*ρ*_BCP_	Δ*ρ*_BCP_	∇^2^ *ρ*_BCP_	ELF_BCP_
N···•···I	−2.821	2.385	1.18	−0.4352	3.897	—	8.825	0.217
I···•···I	−3.993	1.972	2.02	−2.021	6.555	−0.575	1.759	0.695

DFT calculations were extended to compound **4**, aimed at investigating the stability of the [(H_4_ **L_1_**)(**L_1_**)(IBr_2_)]^3+^ twisted prismatic fragment (Figure 4) and eventual anion–π interactions [95, 96] between the [(H_4_ **L_1_**)(**L_1_**)]^4+^ cage and the included [IBr_2_]^−^ anion. The structure of the cationic fragment [(H_4_ **L_1_**)(**L_1_**)(IBr_2_)]^3+^ was successfully optimized and preserved the prismatic motif evidenced in the solid state. The metric parameters for the embedded [IBr_2_]^−^ ion (average I─Br bond distance 2.743 Å) are close to those optimized for the same isolated anion in the gas phase (2.776 Å). Worthy of note, the [(H_4_ **L_1_**)(**L_1_**)]^4+^ cage could be also optimized without the embedded anion. An NCI analysis evidenced only vdW contacts between the anion and the cation (Figure S17). This was supported by a QTAIM analysis (Figure S18), revealing that the terminal atoms of the [IBr_2_]^−^ anion point to the centers of the double bonds of the TTF cores displaying BCPs with parameters typical of very weak closed‐shell interactions (*H*_BCP_ = 8.67·10^−4^, ∇^2^ *ρ*_BCP_ = 0.0209, *ρ* = 7.155·10^–^^3^ a.u.; |*V*_BCP_|/*G*_BCP_ = 0.801). Accordingly, the EDA showed that 92.4% of the interaction energy is due to the electrostatic term of the anion–cation attraction (Δ*E*_int_ = −244.0, Δ*E*_els_ = −225.5, Δ*E*_xrep_ = 71.5, Δ*E*_orb_ = −22.0, Δ*E*_disp_ = −48.6 kcal·mol^−1^). Natural population analysis further evidenced no significative CT between the interhalide and the cationic cage (Σ*Q*_IBr2–_ = 0.930 |e|). Hence, the QM analysis of [(H_4_ **L_1_**)(**L_1_**)(IBr_2_)]^3+^ clearly shows its stability as an independent fragment, the [IBr_2_]^–^ and the [(H_4_ **L_1_**)(**L_1_**)]^4+^ cage interacting mostly due to mutual Coulomb attraction.

## 3 Conclusion

The investigation on the reactivity of the polytopic ligand **L_1_** toward dihalogens and interhalogens (Br_2_, I_2_, ICl, and IBr) reveals a clear dependence on the nature of the halogen reagent. While the softer I_2_ affords the neutral “spoke” CT adduct **1**, the more oxidizing species Br_2_, ICl, and IBr promote the formation of ionic compounds featuring partially or fully protonated ligands [H*_*xx*_* **L_1_**]*^x^* ^+^ (*x* = 3, 4) counterbalanced by tri(inter)halides, such as [Br_3_]^−^ (**2** and **3**), [IBr_2_]^−^ (**4**), and [ICl_2_]^−^ (**5** and **6**). Even in a large molar excess of the most oxidizing dihalogens, the oxidation of the central TTF core was not observed in the reaction of **L_1_** with dihalogens/interhalogens.

These results highlight a marked divergence between neutral CT adduct formation and ionic polyhalide assembly, which can be rationalized in terms of the oxidizing ability and electrophilicity of the halogen species.

The experimental results described in this work, paralleled by DFT QTAIM, NBO, Hirshfeld, and Bader population analyses, and NCI calculations allow an in‐depth understanding of the nature of the bonds and interactions involved in the reaction products. In particular, a detailed analysis of compound **1**, a rare example of a 1:4 donor/diiodine adduct, allowed to evaluate the strength and nature of the donor‐acceptor interaction. This clearly falls in the realm of HaB interactions, for which two concurrent interpretations have been proposed, that is an MO‐mixing, consisting of a partial CT from the nucleophile (Lewis base) to the electrophile (Lewis acid), or a noncovalent σ‐hole interpretation based on the topology of electrostatic potential. Many authors, by means of energy decomposition schemes, have shown that both aspects contribute to σ‐hole interactions in different proportion. Accordingly, in the adduct **L_1_** ·4I_2_ the concomitant contributions of the orbital‐mixing and electrostatic terms were examined, the latter being only partially prevalent according to the EDA, remarking the appropriateness of CT adducts for this class of HaB‐ed compounds. In fact, as observed for different cases of HaB and ChB, the orbital‐mixing and the electrostatic models converge to the same conclusions, the depletion areas of electrostatic potential (σ‐holes) being topologically related to the direction of antibonding natural orbitals of diiodine. The weak strength of the interaction was confirmed by the structural correlations with different three‐body systems and Raman spectroscopy peaks in the region 150–170 cm^−1^.

This study also points out the versatility of **L_1_** and related systems as building blocks for the assembly of supramolecular architectures via NCIs. In fact, in its α_2_α_2_ conformation it is spatially predisposed to assembly around anionic templates in cationic cages of different dimensions upon protonation at the N‐donor atoms. Compound **4** offers a clear demonstration of this ability featuring an uncommon dimeric templated cage [(H_4_ **L_1_**)(**L_1_**)]^4+^ embedding a poorly perturbed [IBr_2_]^−^ anion, which interacts with the host cationic cage predominantly through Coulomb interactions, as demonstrated by QTAIM/NCI/EDA analyses.

Finally, the theoretical calculations on the “spoke” adduct **L_1_** ·4I_2_ show that despite the central TTF core not being directly involved in the interaction with diiodine, its electronic structure is perturbed, with a lowering of the HOMO–LUMO energy gap. The treatment with stoichiometry amounts of diiodine represents a novel approach in tuning the electronic properties of TTF functionalized with N‐donor groups, with potential applications in the field of organic electronics, conductivity, and optoelectronics.

## 4 Experimental Section

Reagents and solvents were obtained from commercial suppliers and used as received. Compound **L_1_** was prepared as previously described [28]. Melting point measurements were carried out using a FALC melting point apparatus mod. C (up to 300°C). FT–IR spectra were collected on KBr pellets in the range 400–4000 cm^−1^ using a Jasco FT/IR‐4X spectrometer. Raman spectroscopy experiments were performed in backscattered geometry with a nonconfocal micro‐Raman OEM system. The emission at 785 nm from a fiber‐coupled laser diode (BWTEK BRM‐785) was focused onto the samples by means of a 40× microscope objective. Raman signals were recorded by a fiber‐coupled grating spectrometer coupled with a Peltier‐cooled CCD (BWTEK BTC667N‐785S) with a spectral resolution of ≤ 5 cm^−1^. The Rayleigh scattering was rejected by means of an edge‐filter cutting at approximately 60 cm^−1^. Depending on the sample under investigation, the laser power was kept below 3 mW to avoid sample decomposition. The Raman spectra were recorded on the same crystals used for the X‐ray diffraction studies. Single‐crystal X‐ray diffraction data [33] were collected at 100 K on a Bruker D8 Venture diffractometer equipped with a PHOTON II detector. Absorption corrections were applied using SADABS‐2016/2 [97]. The structures were solved with ShelXT [98] using dual‐space methods and refined by full‐matrix least‐squares on *F* ^2^ with ShelXL‐2018/3 [99]. The software Olex2 v1.5 [100] was employed for structure manipulation and figure preparation. Hydrogen atoms were positioned geometrically and refined in the riding model with isotropic displacement parameters set at U_iso_(H) = 1.2U_eq_(C/N). For compound **3**, all examined crystals showed twinning; the best‐indexed crystal was selected for data collection. Absorption correction and scaling were carried out with TWINABS 2012/1 [101], and subsequent refinement was performed considering only the major twin domain. In compounds **2–4** and **6**, the presence of highly disordered solvent molecules that could not be satisfactorily modeled was treated with a solvent mask generated by SQUEEZE [43]. Powder X‐ray diffraction (PXRD) analysis of compound **1** was performed on a Bruker D8 Advance diffractometer equipped with a LYNXEYE XE‐T detector, using CuKα radiation (*λ* = 1.5418 Å) operated at 40 kV and 40 mA. Data were collected over the 2θ range of 3–60° with a step size of 0.03° and a counting time of 2 s per step.

### 4.1 Computational Studies

The QM investigation was carried out at the DFT level on **L_1_**, [H_4_ **L_1_**]^+^, [(H_4_ **L_1_**)(**L_1_**)(IBr_2_)]^3+^, [(H_4_ **L_1_**)(**L_1_**)]^4+^, **L_1_** ·4I_2_, I_2_, and [IBr_2_]^−^ (Tables S16–S25). All calculations were performed with the Gaussian 16 computational suite [102] on the Galileo100 supercomputer at the CINECA High Performance Computing facilities. The choice of the computational level was carried out by comparing the average the N···I and I─I structural distances with the corresponding values optimized in the **L_1_** ·4I_2_ model adduct. The functionals M06–2X [103], mPW1PW [64], and *ω*B97X–D [104] (Def2SVP(C, H, N, S) [65] / LANL08(d) [66] Basis Sets) yielded similar results, the latter providing a slightly larger root mean square deviation ([1/2·(Δ*d*_N···I_^2^ + Δ*d*_I─I_^2^)]^1/2^ = 0.099, 0.104, and 0.148 Å, respectively). The use of a mixed‐BS approach, previously adopted by several authors for different inorganic and organic molecular systems [105, 106] slightly improved the agreement between structural and optimized metric parameters ([1/2·(Δ*d*_N···I_^2^ + Δ*d*_I─I_^2^)]^1/2^ = 0.131 and 0.108 Å for Def2TZVP(all atoms) [65] and Def2TZVP(C, H, N, S)/LANL08(d)(I), respectively; Tables S22, S23, and S26). Based on these results, previously published benchmarks [107], and comparative theoretical investigations reported previously for related compounds [7, 8, 9, 11, 108], calculations were carried out by adopting the mPW1PW functional [64] and the Def2TZVP [65] basis sets for all atomic species with the exception of halogens, for which the LANL08(d) [66] BS was preferred. Basis sets were obtained from the Basis Set Exchange database [109]. The memory required for each calculation was evaluated by the GaussMem cross‐platform (Linux, macOS, Windows) program as a function of the number of shared processors, the total number of basis set functions, and a memory threshold depending on the highest angular momentum basis function [23, 110]. The nature of the minima of each optimized geometry was verified by harmonic frequency calculations. Thermochemical calculations (*T* = 298.15 K) were carried out at the optimized geometries as a part of harmonic frequency calculations. Charge distributions were evaluated according to the Hirshfeld [67], Bader [90], and NBO level [68, 69, 70, 111] partitioning schemes at the optimized geometries. Mayer bond orders [71] were calculated with Chemissian 4.67 [112]. GaussView 6.0.16 [113] was used to investigate the optimized structures and the Laplacian of the total electron density and to generate MEP energy maps. Hirshfeld and Bader analyses were carried out with the cross‐platform (Windows, Linux) program Multiwfn [114, 115]. In the search for BCPs, the Poincaré–Hopf relationship for molecules was satisfied (*n* = number of BCPs) [90]: *n*(3,−3)−*n*(3,−1) + *n*(3, + 1)—*n*(3,+ 3) = 1. The NCI [87, 88] and sobEDAw [89] analyses were carried out with Multiwfn [114, 115]. The partitioning of the interaction energy was carried out at B3LYP‐D3/Def2TZVP‐w, CB level [116, 117, 118, 119] at the geometry optimized at mPW1PW//Def2TZVP/LANL08(d) level.

### 4.2 Synthesis

**Compound L_1_·4I_2_ (1)**. A chloroform solution of diiodine (2.0·10^−2^ mol·L^−1^; 2.0 mL) was added to a solution of **L_1_** (5.0·10^−3^ mol·L^−1^; 2.0 mL) in the same solvent. The resulting mixture was stirred at room temperature for 5 min and then allowed to undergo slow solvent evaporation. After 4 days, orange crystals of **1** suitable for single‐crystal X‐ray diffraction were collected and structurally characterized. The identity of the compound could only be verified by X‐ray diffraction analysis because of the very low isolable amount (it was not possible to determine the respective yield). M. p. = 155°C. Raman (100–300 cm^−1^; relative intensity in parenthesis): 166 (10) cm^−1^. FT–IR (400–4000 cm^−1^): 3435 (m), 3068 (w), 2918 (w), 1619 (s), 1577 (s), 1569 (s), 1542 (w), 1478 (vs), 1403 (m), 1385 (m), 1260 (w), 1203 (w), 1096 (m), 1059 (m), 1013 (w), 901 (w), 804 (vs), 768 (w), 713 (w), 610 (w), 556 (v), and 485 (m) cm^−1^.

**Compounds [H_4_L_1_](Br)_2_[Br_3_]_2_·0.7CHCl_3_ (2) and [H_3_L_1_](Br)_2_[Br_3_]·(Br_2_)_0.64_·2CHCl_3_ (3)**. A sixfold molar excess of dibromine (1.5·10^−2^ mol·L^−1^; 4.0 mL) in chloroform was carefully placed at the bottom of a 10 mm diameter test tube, followed by a layer of 2 mL of a 1:1 (*v/v*) CHCl_3_/CH_3_CN solution. Finally, a solution of **L_1_** in CH_3_CN (2.5·10^−3^ mol·L^−1^; 4.0 mL) was gently layered on top. The tube was left undisturbed, and crystals formed at the solvent interface over 72 h. Inspection under optical microscope revealed the presence of crystals with two different habits, red platelets and orange blocks, which were isolated and structurally characterized as compounds **2** and **3**, respectively. The identity of the compound could only be verified by X‐ray diffraction analysis because of the very low isolable amount (it was not possible to determine the respective yield). Compound **2**: M. p. = 83°C dec. FT–IR (400–4000 cm^−1^): 3401 (s), 3199 (s), 3117 (s), 3082 (s), 3035 (s), 2972 (s), 2918 (s), 2085 (s), 2610 (m), 2363 (w), 1620 (vs), 1477 (s), 1401(m), 1385 (m), 1296 (w), 1194 (m), 1099 (m), 1047 (w), 992 (w), 906 (w), 793 (m), 706 (w), 600 (w), and 477 (w) cm^−1^. Compound **3**: M. p. = 97°C dec. FT–IR (400–4000 cm^−1^): 3411 (s), 3199 (m), 3117 (m), 3083 (m), 3042 (m), 2976 (m), 2916 (m), 2811 (m), 2611 (w), 1620 (vs), 1477 (s), 1400 (w), 1385 (w), 1366 (w), 1240 (w), 1195 (w), 1099 (m), 1046 (w), 990 (w), 906 (w), 791 (m), 760 (w), 705 (w), 669 (w), 644 (w), 600 (w), 534 (w), 501 (w), 477 (w), 447 (w), and 421 (w) cm^−1^.

**Compound [H_4_L_1_](L_1_)(IBr_2_)_4_·2CH_3_CN·2.4CHCl_3_ (4)**. A sixfold molar excess of iodine monobromide (1.5·10^−2^ mol·L^−1^; 4 mL) in chloroform was carefully placed at the bottom of a 10 mm diameter test tube, then overlayed with 2 mL of a 1:1 (*v/v*) CHCl_3_/CH_3_CN mixture. A solution of **L_1_** in CH_3_CN (2.5·10^−3^ mol·L^−1^; 4.0 mL) was gently layered on top. The tube was left undisturbed, and red prismatic crystals of compound **4** formed at the solvent interface over a period of two weeks. The identity of the compound could only be verified by X‐ray diffraction analysis because of the very low isolable amount (it was not possible to determine the respective yield). M. p. = 142°C. Raman (100–300 cm^−1^; relative intensity in parenthesis): 166 (10), 160sh (9), 147 (8), 128 (4) cm^−1^. FT–IR (400–4000 cm^−1^): 3430 (s), 3169 (m), 3082 (m), 3054 (m), 2923 (s), 2851 (m), 1710 (m), 1621 (vs), 1580 (m), 1478 (m), 1467 (s), 1384 (m), 1189 (m), 1099 (m), 1051 (w), 996 (w), 903 (w), 792 (m), 758 (w), 709 (w), and 476 (w) cm^−1^.

**Compounds [H_4_L_1_][ICl_2_]_2_(Cl)_2_·CH_3_CN·2CHCl_3_ (5) and [H_3_L_1_][H_4_L_1_][ICl_2_]_3_(Cl)_4_·CHCl_3_·1.5I_2_ (6)**. A sixfold molar excess of iodine monochloride (2.0·10^−2^ mol·L^−1^; 4.0 mL) in chloroform was carefully placed at the bottom of a 10 mm diameter test tube, followed by a layer of 2 mL of a 1:1 (*v*/*v*) CHCl_3_/CH_3_CN solution. Finally, a solution of **L_1_** in CH_3_CN (3.3·10^−3^ mol·L^−1^; 3.0 mL) was gently layered on top. The tube was left undisturbed, and crystals formed at the solvent interface over two weeks. Inspection under optical microscope revealed the presence of plank‐shaped yellow and red crystals that were isolated and structurally characterized as compounds **5** and **6**, respectively. The identity of the compound could only be verified by X‐ray diffraction analysis because of the very low isolable amount (it was not possible to determine the respective yield). Raman (100–300 cm^−1^; relative intensity in parenthesis): 263 (10) cm^−1^. Compound **5**: M. p. = 110°C dec. FT–IR (400–4000 cm^−1^): 3391 (vs), 3118 (vs), 3042 (vs), 1623 (s), 1478 (m), 1402 (s), 1196 (vs), 1101 (s), 870 (w), 827 (w), 803 (w), 761 (w), 746 (w), 720 (w), 598 (s), 478 (w), and 424 (w) cm^−1^. Compound **6**: M. p. = 135°C dec. FT–IR (400–4000 cm^−1^): 3436 (m), 2925 (vs), 2853 (s), 1735 (s), 1621 (s), 1469 (s), 1402 (m), 1383 (m), 1373 (m), 1300 (w), 1244 (m), 1196 (s), 1098 (s), 1038 (m), 997 (w), 948 (w), 925 (w), 904 (w), 796 (s), 743 (m), 710 (w), 644 (w), 620 (w), 599 (w), 577 (w), 503 (w), and 473 (w) cm^−1^.

## Conflicts of Interest

The authors declare no conflicts of interest.

## Supporting information




**Supporting File 1**: chem71483‐sup‐0001‐SuppMat.pdf.

## Data Availability

Structural data have been deposited at the joint Cambridge Crystallographic Data Centre and Fachinformationszentrum Karlsruhe Access Structures service (2549239–2549244). The data that supports the findings of this investigation are available in the Supporting Information of this article.
