# Supplementary material for: From Charge‐Transfer Adducts to (Tri)Halide‐Templated Supramolecular Cages: Covalent vs Ionic Products in the Reactions of Pyridin‐4‐ylthio‐Substituted Tetrathiafulvalene With Dihalogens and Interhalogens
Source: Chemistry. 2026 Aug 5;32(41):e71483. doi: 10.1002/chem.71483 (PMC13730010; doi:10.1002/chem.71483)
Supplement: Supplementary file 1 [file CHEM-32-e71483-s001.pdf]

Supporting Information

From Charge-Transfer Adducts to (Tri)halide-Templated Supramolecular Cages: Covalent vs Ionic Products in the Reactions of Pyridin-4-ylthio-Substituted Tetrathiafulvalene with Dihalogens and Interhalogens

Anna Caria,[1] Stephane A. Baudron,[2] M. Carla Aragoni,[1] Vito Lippolis,[1,3] Anna Pintus,[1] Enrico Podda[*1,3] and Massimiliano Arca[*1]

[1] Dipartimento di Scienze Chimiche e Geologiche, Università degli Studi di Cagliari, S. S. 554 Bivio per Sestu, 09042 Monserrato (Cagliari), Italy.
E-mail: marca@unica.it

[2] Université de Strasbourg, CNRS, CMC UMR 7140, 4 rue Blaise Pascal, F-67000, Strasbourg, France.

[3] Centro Servizi di Ateneo per la Ricerca (CeSAR), Università degli Studi di Cagliari, S. S. 554 Bivio Sestu, 09042 Monserrato (Cagliari), Italy
E-mail: enrico.podda@unica.it

Table S1. Selected crystal data and structure refinement parameters for compounds **1**–**6**.

Compound	**1**	**2**	**3**	**4**	**5**	**6**
Empirical formula	$C_{26}H_{16}I_8N_4S_8$	$C_{27}H_{21}Br_8Cl_3N_4S_8$	$C_{28}H_{21}Br_{6.28}Cl_6N_4S_8$	$C_{56}H_{42}Br_8I_4N_{10}S_{16}$	$C_{30}H_{25}Cl_{12}I_2N_5S_8$	$C_{53}H_{40}Cl_{13}I_6N_8S_{16}$
Formula weight	1656.11	1403.59	1384.50	2514.83	1391.23	2524.14
Temperature / K	100(2)	100(2)	100(2)	100(2)	100(2)	100(2)
Crystal system	monoclinic	triclinic	monoclinic	orthorhombic	triclinic	monoclinic
Space group	$P2_1/c$	$P\bar{1}$	$C2/m$	*Cmcm*	$P\bar{1}$	$P2_1/c$
a /Å	18.7249(15)	12.261(6)	31.180(3)	20.507(2)	12.2745(9)	12.1752(12)
b / Å	17.1615(14)	14.952(9)	11.9028(10)	30.250(3)	14.3171(11)	29.086(3)
c / Å	12.8427(9)	15.337(7)	12.9595(12)	14.7867(17)	16.2246(13)	24.978(3)
α / °	90	86.901(19)	90	90	103.047(3)	90
β / °	96.191(3)	68.125(14)	99.879(4)	90	90.188(3)	98.709(4)
γ / °	90	66.87(2)	90	90	113.012(3)	90
Volume / Å^3	4102.9(6)	2386(2)	4738.4(7)	9172.8(18)	2543.2(3)	8743.4(17)
Z	4	2	4	4	2	4
ρ_{calc} / g·cm^{-3}	2.681	1.954	1.941	1.821	1.817	1.918
μ / mm^{-1}	6.482	7.268	6.039	5.246	2.226	2.951
Crystal size / mm^3	0.19 × 0.03 × 0.02	0.30 × 0.16 × 0.04	0.42 × 0.14 × 0.12	0.25 × 0.24 × 0.20	0.30 × 0.08 × 0.03	0.26 × 0.12 × 0.03
Reflections collected	49706	101978	107677	74967	52724	156969
Ind. refls. (R_{int})	9401 (0.0604)	10841 (0.0988)	4567 (0.0881)	5605 (0.0891)	11650 (0.0721)	16000 (0.0791)
GooF on F^2	1.042	1.014	1.054	1.055	1.022	1.027
Final R indexes [I ≥ 2σ (I)]	R_1 = 0.0331<br>wR_2 = 0.0592	R_1 = 0.0433<br>wR_2 = 0.0974	R_1 = 0.0692<br>wR_2 = 0.1442	R_1 = 0.0456<br>wR_2 = 0.0994	R_1 = 0.0400<br>wR_2 = 0.1000	R_1 = 0.0385<br>wR_2 = 0.0869
Final R indexes [all data]	R_1 = 0.0585<br>wR_2 = 0.0670	R_1 = 0.0661<br>wR_2 = 0.1066	R_1 = 0.0901<br>wR_2 = 0.1531	R_1 = 0.0641<br>wR_2 = 0.1103	R_1 = 0.0552<br>wR_2 = 0.1102	R_1 = 0.0474<br>wR_2 = 0.0915
Largest diff. peak/hole / e·Å^{-3}	0.92/−1.12	1.69/−1.93	1.89/−1.42	2.03/−1.19	1.62/−2.33	2.50/−1.83

Table S2. Selected bond lengths (Å) for compound **1**.

I1–I2	2.7879(5)	N2–C12	1.337(7)
I1–N1	2.441(5)	N2–C13	1.332(7)
I3–I4	2.8015(6)	N3–C19	1.334(8)
I3–N2	2.378(5)	N3–C20	1.341(7)
I5–I6	2.7662(6)	N4–C24	1.330(8)
I5–N3	2.455(5)	N4–C25	1.328(7)
I7–I8	2.7675(6)	C1–C2	1.339(7)
I7–N4	2.488(5)	C3–C4	1.334(7)
S1–C1	1.751(5)	C5–C6	1.381(8)
S1–C3	1.756(5)	C5–C9	1.398(7)
S2–C1	1.751(5)	C6–C7	1.378(7)
S2–C4	1.746(5)	C8–C9	1.358(7)
S3–C3	1.745(5)	C10–C11	1.373(8)
S3–C5	1.761(5)	C10–C14	1.390(7)
S4–C4	1.759(5)	C11–C12	1.378(8)
S4–C10	1.759(6)	C13–C14	1.379(8)
S5–C2	1.757(5)	C15–C16	1.341(7)
S5–C15	1.754(6)	C17–C18	1.397(8)
S6–C2	1.761(5)	C17–C21	1.389(8)
S6–C16	1.746(5)	C18–C19	1.366(8)
S7–C15	1.762(5)	C20–C21	1.372(8)
S7–C17	1.761(6)	C22–C23	1.390(8)
S8–C16	1.756(5)	C22–C26	1.390(7)
S8–C22	1.777(6)	C23–C24	1.381(8)
N1–C7	1.334(7)	C25–C26	1.370(8)
N1–C8	1.338(7)		

Table S3. Selected bond angles (°) for compound **1**.

N1–I1–I2	172.66(11)	C3–C4–S4	124.2(4)
N2–I3–I4	177.06(11)	C6–C5–S3	127.3(4)
N3–I5–I6	176.34(11)	C6–C5–C9	118.6(5)
N4–I7–I8	176.82(11)	C9–C5–S3	114.1(4)
C1–S1–C3	94.6(2)	C7–C6–C5	118.6(5)
C4–S2–C1	94.2(3)	N1–C7–C6	122.8(5)
C3–S3–C5	106.6(3)	N1–C8–C9	123.1(5)
C4–S4–C10	102.3(3)	C8–C9–C5	118.7(5)
C15–S5–C2	94.2(2)	C11–C10–S4	125.4(4)
C16–S6–C2	94.3(2)	C11–C10–C14	118.3(5)
C17–S7–C15	99.7(3)	C14–C10–S4	116.3(4)
C16–S8–C22	102.3(2)	C10–C11–C12	119.5(5)
C7–N1–I1	124.9(4)	N2–C12–C11	121.9(5)
C7–N1–C8	118.1(5)	N2–C13–C14	121.5(5)
C8–N1–I1	116.3(4)	C13–C14–C10	119.4(5)
C12–N2–I3	118.7(4)	S5–C15–S7	117.9(3)
C13–N2–I3	121.9(4)	C16–C15–S5	117.5(4)
C13–N2–C12	119.4(5)	C16–C15–S7	124.5(4)
C19–N3–I5	126.6(4)	S6–C16–S8	117.6(3)
C19–N3–C20	117.9(5)	C15–C16–S6	117.2(4)
C20–N3–I5	115.5(4)	C15–C16–S8	124.9(4)
C24–N4–I7	121.0(4)	C18–C17–S7	119.1(4)
C25–N4–I7	120.2(4)	C21–C17–S7	122.7(4)
C25–N4–C24	118.7(5)	C21–C17–C18	118.1(5)
S1–C1–S2	114.2(3)	C19–C18–C17	118.5(6)
C2–C1–S1	124.3(4)	N3–C19–C18	123.6(5)
C2–C1–S2	121.5(4)	N3–C20–C21	122.6(6)
S5–C2–S6	114.0(3)	C20–C21–C17	119.3(5)
C1–C2–S5	125.0(4)	C23–C22–S8	117.1(4)
C1–C3–S6	121.0(4)	C23–C22–C26	118.5(5)
S3–C3–S1	121.0(3)	C26–C22–S8	124.3(4)
C4–C3–S1	116.7(4)	C24–C23–C22	118.5(5)
C4–C3–S3	120.0(4)	N4–C24–C23	122.5(6)
S2–C4–S4	116.8(3)	N4–C25–C26	123.1(5)
C3–C4–S2	117.9(4)	C25–C26–C22	118.6(5)

Table S4. Selected bond lengths (Å) for compound **2**.

Br6–Br5	2.5372(15)	N1–C1	1.339(8)
Br6–Br7	2.5451(15)	N1–C5	1.335(8)
Br2–Br1	2.5445(13)	N3–C25	1.347(8)
Br2–Br1^{i}	2.5445(13)	N3–C24	1.337(8)
Br8–Br9A^{ii}	2.518(2)	C21–C17	1.388(7)
Br8–Br9A	2.518(2)	C21–C20	1.374(7)
Br8–Br9B	2.539(4)	C13–C14	1.329(7)
Br8–Br9B^{ii}	2.539(4)	C12–C11	1.349(7)
S4–C13	1.766(5)	C16–C15	1.363(7)
S4–C12	1.773(5)	C17–C18	1.401(7)
S3–C13	1.775(5)	C9–C8	1.385(7)
S3–C11	1.764(5)	C9–C10	1.405(7)
S7–C17	1.758(5)	C22–C23	1.397(7)
S7–C15	1.739(5)	C22–C26	1.393(7)
S6–C16	1.742(5)	C7–C6	1.372(8)
S6–C14	1.761(5)	C7–C8	1.397(7)
S1–C11	1.747(5)	C20–N4	1.339(9)
S1–C3	1.758(5)	C23–C24	1.375(9)
S5–C14	1.769(5)	C25–C26	1.373(8)
S5–C15	1.762(5)	C19–N4	1.331(9)
S2–C12	1.758(5)	C19–C18	1.368(8)
S2–C8	1.765(5)	C2–C1	1.360(8)
S8–C16	1.765(5)	C2–C3	1.396(8)
S8–C22	1.757(6)	C4–C3	1.396(7)
N2–C6	1.336(8)	C4–C5	1.381(7)
N2–C10	1.325(7)		

^{i} = 1−*x*, 1−*y*, 1−*z*; ^{ii} = 1−*x*, 2−*y*, −*z*.

Table S5. Selected bond angles (°) for compound **2**.

Br5–Br6–Br7	178.70(3)	C18–C17–S7	115.8(4)
Br1^{i}–Br2–Br1	180.0	C8–C9–C10	118.3(5)
Br9A–Br8–Br9A^{ii}	180.0	C23–C22–S8	117.8(4)
Br9B–Br8–Br9B^{ii}	180.0	C26–C22–S8	123.1(4)
C13–S4–C12	92.0(2)	C26–C22–C23	119.1(5)
C11–S3–C13	92.5(2)	C6–C7–C8	118.8(5)
C15–S7–C17	102.5(2)	N4–C20–C21	120.5(6)
C16–S6–C14	94.8(2)	C24–C23–C22	119.0(6)
C11–S1–C3	100.2(2)	N3–C25–C26	120.3(6)
C15–S5–C14	95.1(2)	N3–C24–C23	120.7(5)
C12–S2–C8	102.1(2)	N4–C19–C18	120.3(6)
C22–S8–C16	102.1(2)	C1–C2–C3	119.8(6)
C10–N2–C6	122.7(5)	N1–C1–C2	120.3(5)
C5–N1–C1	122.2(5)	S7–C15–S5	119.5(3)
C24–N3–C25	121.5(5)	C16–C15–S7	124.2(4)
C20–C21–C17	118.7(5)	C16–C15–S5	115.9(4)
S4–C13–S3	112.1(3)	C25–C26–C22	119.3(5)
C14–C13–S4	123.5(4)	C19–N4–C20	122.1(5)
C14–C13–S3	124.3(4)	C5–C4–C3	119.2(5)
S2–C12–S4	119.0(3)	N2–C6–C7	120.4(5)
C11–C12–S4	116.9(4)	C9–C8–S2	123.5(4)
C11–C12–S2	123.8(4)	C9–C8–C7	119.9(5)
S6–C16–S8	116.6(3)	C7–C8–S2	116.6(4)
C15–C16–S6	118.6(4)	C2–C3–S1	117.7(4)
C15–C16–S8	123.6(4)	C2–C3–C4	118.5(5)
S1–C11–S3	118.9(3)	C4–C3–S1	123.8(4)
C12–C11–S3	116.5(4)	N1–C5–C4	120.0(5)
C12–C11–S1	124.4(4)	C19–C18–C17	119.1(6)
S6–C14–S5	114.4(3)	N2–C10–C9	119.9(5)
C13–C14–S6	123.0(4)	C13–C14–S5	122.5(4)
		C21–C17–S7	125.0(4)
		C21–C17–C18	119.2(5)

^{i} = 1−x, 1−y, 1−z; ^{ii} = 1−x, 2−y, −z.

Table S6. Selected bond lengths (Å) for compound **3**.

Br4–Br5	2.5104(16)	N1–C1	1.340(12)
Br4–Br3	2.5572(17)	N1–C5	1.354(11)
Br7–Br7^{ii}	2.536(3)	C9–C9^{i}	1.344(13)
Br6–Br6^{iii}	2.43(3)	C6–C6^{i}	1.349(14)
S3–C8	1.757(6)	N2–C13	1.349(13)
S3–C9	1.750(7)	N2–C12	1.322(16)
S2–C7	1.765(6)	C3–C2	1.408(11)
S2–C6	1.741(7)	C3–C4	1.373(11)
S1–C6	1.746(7)	C2–C1	1.350(12)
S1–C3	1.745(9)	C4–C5	1.366(11)
S4–C9	1.752(7)	C10–C14	1.381(13)
S4–C10	1.733(9)	C10–C11	1.405(11)
Cl2–C15	1.751(12)	C15–Cl3	1.692(11)
Cl1–C15	1.924(4)	C14–C13	1.373(13)
C8–C7	1.319(14)	C11–C12	1.359(17)

^{i} = +*x*, 1−*y*, +*z*; ^{ii} = 1−*x*, +*y*, 1−*z*; ^{iii} = +*x*, 2−*y*, +*z*.

Table S7. Selected bond angles (°) for compound **3**.

Br5–Br4–Br3	173.13(7)	C6^{i}–C6–S1	124.6(2)
C9–S3–C8	93.4(4)	C12–N2–C13	123.9(10)
C6–S2–C7	94.0(4)	C2–C3–S2	116.4(6)
C3–S1–C6	102.0(4)	C4–C3–S1	124.9(6)
C10–S4–C9	102.5(4)	C4–C3–C2	118.7(8)
S3^{i}–C8–S3	113.3(6)	C1–C2–C3	119.6(8)
C7–C8–S3	123.3(3)	C5–C4–C3	119.8(8)
C7–C8–S3^{i}	123.3(3)	C14–C10–S4	125.4(6)
C1–N1–C5	121.2(8)	C14–C10–C11	117.5(9)
S2–C7–S2^{i}	112.7(5)	C11–C10–S4	117.1(9)
C8–C7–S2^{i}	123.6(3)	N1–C1–C2	120.6(8)
C8–C7–S2	123.6(3)	C13–C14–C10	119.9(8)
S3–C9–S4	118.3(4)	N1–C5–C4	120.1(8)
C9^{i}–C9–S3	117.1(2)	C12–C11–C10	121.5(12)
C9^{i}–C9–S4	124.4(2)	N2–C13–C14	119.0(11)
S2–C6–S1	117.9(4)	N2–C12–C11	118.1(9)
C6^{i}–C6–S2	117.2(2)		

^{i} = +*x*, 1−*y*, +*z*

Table S8. Selected bond lengths (Å) for compound **4**.

I3–Br5[i]	2.6823(8)	N2–C8	1.341(9)
I3–Br5	2.6823(8)	N1–C5	1.331(8)
I1–Br2	2.6765(15)	N1–C6	1.326(9)
I1–Br1	2.7241(15)	C4–C3	1.385(9)
I2–Br4	2.7095(11)	C4–C5	1.373(9)
I2–Br3	2.7487(11)	C2–C2[ii]	1.348(12)
S4–C14	1.755(5)	C11–C10	1.382(8)
S4–C13	1.749(6)	C11–C12	1.370(8)
S3–C13	1.752(6)	C13–C13[iii]	1.344(11)
S3–C10	1.764(6)	C3–C7	1.383(9)
S2–C2	1.746(6)	C10–C9	1.393(8)
S2–C3	1.775(6)	N3–C15	1.080(16)
S1–C2	1.760(6)	C9–C8	1.369(9)
S1–C1	1.761(5)	C1–C1[iv]	1.333(17)
C14–C14[ii]	1.344(17)	C16–C15	1.509(19)
N2–C12	1.331(8)	C7–C6	1.390(10)

[i] = 1−*x*, −*y*, 1−*z*; [ii] = +*x*, +*y*, 3/2−*z*; [iii] = 1−*x*, +*y*, +*z*; [iv] = 1−*x*, +*y*, 3/2−*z*.

Table S9. Selected bond angles (°) for compound **4**.

$Br5^{i}$–I3–Br5	180	$C13^{ii}$–C13–S4	117.28(19)
Br2–I1–Br1	180	$C13^{ii}$–C13–S3	125.1(2)
Br4–I2–Br3	177.26(4)	C4–C3–S2	118.2(5)
C13–S4–C14	94.0(3)	C7–C3–S2	122.8(5)
C13–S3–C10	101.7(3)	C7–C3–C4	119.0(6)
C2–S2–C3	101.9(3)	C11–C10–S3	123.6(4)
C2–S1–C1	94.8(3)	C11–C10–C9	118.1(6)
S4–C14–$S4^{ii}$	114.2(5)	C9–C10–S3	118.3(5)
$C14^{iii}$–C14–$S4^{ii}$	122.9(2)	N1–C5–C4	122.9(6)
$C14^{iii}$–C14–S4	122.9(2)	C8–C9–C10	118.9(6)
C12–N2–C8	119.6(6)	$S1^{iii}$–C1–S1	113.4(5)
C6–N1–C5	118.7(6)	$C1^{iv}$–C1–S1	123.3(2)
C5–C4–C3	118.6(6)	$C1^{iv}$–C1–$S1^{iii}$	123.3(2)
S2–C2–S1	116.9(4)	N2–C12–C11	121.3(6)
$C2^{iii}$–C2–S2	125.9(2)	N2–C8–C9	122.1(6)
$C2^{iii}$–C2–S1	116.9(2)	C3–C7–C6	118.3(6)
C12–C11–C10	120.1(6)	N3–C15–C16	179.5(12)
S4–C13–S3	117.3(3)	N1–C6–C7	122.4(6)

^{i} = 1−*x*, −*y*,1−*z*; ^{ii} = 1−*x*, +*y*, +*z*; ^{iii} = +*x*, +*y*, 3/2−*z*; ^{iv} = 1−*x*, +*y*, 3/2−*z*.

Table S10. Selected bond lengths (Å) for compound **5**.

I1–Cl3	2.6162(12)	N2–C11	1.337(6)
I1–Cl4	2.4810(12)	N3–C19	1.339(6)
I2–Cl5	2.5336(15)	N3–C20	1.330(6)
I2–Cl5^{i}	2.5336(15)	N4–C25	1.328(6)
I3–Cl6^{ii}	2.5515(11)	N4–C24	1.334(6)
I3–Cl6	2.5515(11)	C13–C12	1.367(6)
S1–C3	1.750(4)	C13–C9	1.391(6)
S1–C6	1.755(4)	C1–C2	1.355(6)
S2–C7	1.756(4)	C11–C10	1.368(6)
S2–C9	1.759(4)	C2–C3	1.397(6)
S3–C6	1.747(4)	C3–C4	1.392(5)
S3–C8	1.755(4)	C4–C5	1.366(6)
S4–C7	1.755(4)	C6–C7	1.343(6)
S4–C8	1.760(4)	C8–C14	1.338(5)
S5–C14	1.760(4)	C15–C16	1.352(5)
S5–C15	1.761(4)	C9–C10	1.390(6)
S6–C14	1.755(4)	C17–C18	1.397(6)
S6–C16	1.764(4)	C17–C21	1.393(6)
S7–C15	1.745(4)	C18–C19	1.368(6)
S7–C17	1.755(4)	C20–C21	1.370(6)
S8–C16	1.740(4)	C22–C26	1.393(5)
S8–C22	1.755(4)	C22–C23	1.386(5)
N1–C1	1.339(6)	C26–C25	1.366(6)
N1–C5	1.338(6)	C24–C23	1.378(6)
N2–C12	1.330(6)		

^{i} = −*x*, 1−*y*, −*z*; ^{ii} = 1−*x*, −*y*, −*z*.

Table S11. Selected bond angles (°) for compound **5**.

Cl4–I1–Cl3	179.27(4)	S3–C8–S4	114.8(2)
Cl5^{i}–I2–Cl5	180.0	C14–C8–S3	122.9(3)
Cl6–I3–Cl6^{ii}	180.0	C14–C8–S4	122.3(3)
C3–S1–C6	101.82(19)	S6–C14–S5	112.6(2)
C7–S2–C9	103.4(2)	C8–C14–S5	123.5(3)
C6–S3–C8	94.7(2)	C8–C14–S6	123.4(3)
C7–S4–C8	95.0(2)	S7–C15–S5	118.5(2)
C14–S5–C15	91.49(18)	C16–C15–S5	116.6(3)
C14–S6–C16	91.39(19)	C16–C15–S7	124.5(3)
C15–S7–C17	100.99(18)	C13–C9–S2	123.3(3)
C16–S8–C22	101.66(18)	C13–C9–C10	119.7(4)
C5–N1–C1	121.9(4)	C10–C9–S2	117.0(3)
C12–N2–C11	122.4(4)	S8–C16–S6	119.4(2)
C20–N3–C19	122.3(4)	C15–C16–S6	116.4(3)
C25–N4–C24	122.7(4)	C15–C16–S8	123.8(3)
C12–C13–C9	118.9(4)	C18–C17–S7	116.7(3)
N1–C1–C2	120.5(4)	C21–C17–S7	123.9(3)
N2–C12–C13	120.1(4)	C21–C17–C18	119.3(4)
N2–C11–C10	120.2(4)	C19–C18–C17	118.6(4)
C1–C2–C3	119.3(4)	N3–C19–C18	120.5(4)
C2–C3–S1	116.9(3)	N3–C20–C21	120.2(4)
C4–C3–S1	124.3(3)	C20–C21–C17	119.0(4)
C4–C3–C2	118.8(4)	C11–C10–C9	118.6(4)
C5–C4–C3	119.4(4)	C26–C22–S8	116.6(3)
N1–C5–C4	120.1(4)	C23–C22–S8	122.9(3)
S3–C6–S1	116.7(3)	C23–C22–C26	120.5(4)
C7–C6–S1	124.7(3)	C25–C26–C22	118.0(4)
C7–C6–S3	118.2(3)	N4–C25–C26	120.6(4)
S4–C7–S2	118.0(3)	N4–C24–C23	119.8(4)
C6–C7–S2	124.7(3)	C24–C23–C22	118.3(4)
C6–C7–S4	116.9(3)		

^{i} = −*x*, 1−*y*, −*z*; ^{ii} = 1 −*x*, −*y*, −*z*.

Table S12. Selected bond lengths (Å) for compound **6**.

I1–Cl1	2.5595(19)	C20–C21	1.366(6)
I1–Cl2	2.5291(17)	C22–C23	1.386(7)
I2–Cl3	2.5282(11)	C22–C26	1.393(7)
I2–Cl4	2.5458(11)	C23–C24	1.411(8)
I3–Cl5	2.5888(14)	C25–C26	1.357(7)
I3–Cl6	2.5642(16)	S9–C27	1.759(5)
S1–C1	1.752(6)	S9–C32	1.754(4)
S1–C6	1.744(4)	S10–C33	1.748(4)
S2–C7	1.748(4)	S10–C34	1.755(4)
S2–C8	1.764(5)	S11–C32	1.750(4)
S3–C6	1.754(4)	S11–C39	1.757(4)
S3–C13	1.751(4)	S12–C33	1.763(4)
S4–C7	1.760(4)	S12–C39	1.755(4)
S4–C13	1.764(4)	S13–C40	1.766(4)
S5–C14	1.762(4)	S13–C41	1.754(4)
S5–C16	1.761(4)	S14–C40	1.752(4)
S6–C14	1.760(4)	S14–C42	1.751(4)
S6–C15	1.749(4)	S15–C41	1.751(4)
S7–C16	1.746(4)	S15–C43	1.751(4)
S7–C22	1.762(5)	S16–C42	1.752(4)
S8–C15	1.749(4)	S16–C48	1.745(5)
S8–C17	1.754(4)	N5–C29	1.341(7)
N1A–C3	1.357(11)	N5–C30	1.328(6)
N1A–C4A	1.328(13)	N6–C36	1.332(6)
N1B–C3	1.371(12)	N6–C37	1.332(6)
N1B–C4B	1.330(14)	N7–C45	1.336(6)
N2–C10	1.284(9)	N7–C46	1.329(7)
N2–C11	1.260(10)	N8–C50	1.332(8)
N3–C19	1.334(6)	N8–C51	1.358(10)
N3–C20	1.337(6)	C27–C28	1.402(6)
N4–C24	1.318(9)	C27–C31	1.390(7)
N4–C25	1.309(8)	C28–C29	1.359(7)
C1–C2	1.380(8)	C30–C31	1.372(7)
C1–C5A	1.431(10)	C32–C33	1.344(6)
C1–C5B	1.414(13)	C34–C35	1.392(6)
C2–C3	1.362(8)	C34–C38	1.395(6)

C4A–C5A	1.377(13)	C35–C36	1.366(6)
C4B–C5B	1.377(15)	C37–C38	1.361(6)
C6–C7	1.341(6)	C39–C40	1.335(6)
C8–C9	1.368(8)	C41–C42	1.346(6)
C8–C12	1.374(7)	C43–C44	1.384(6)
C9–C10	1.358(8)	C43–C47	1.400(6)
C11–C12	1.421(10)	C44–C45	1.364(6)
C13–C14	1.333(6)	C46–C47	1.367(7)
C15–C16	1.343(6)	C48–C49	1.384(7)
C17–C18	1.386(6)	C48–C52	1.391(8)
C17–C21	1.392(6)	C49–C50	1.372(7)
C18–C19	1.371(6)	C51–C52	1.349(10)

Table S13. Selected bond angles (°) for compound **6**.

Cl2–I1–Cl1	179.65(5)	C22–C23–C24	117.0(6)
Cl3–I2–Cl4	173.55(4)	N4–C24–C23	120.1(5)
Cl6–I3–Cl5	177.97(5)	N4–C25–C26	120.1(6)
C6–S1–C1	103.6(2)	C25–C26–C22	119.7(6)
C7–S2–C8	102.4(2)	C32–S9–C27	101.2(2)
C13–S3–C6	93.5(2)	C33–S10–C34	101.79(19)
C7–S4–C13	92.9(2)	C32–S11–C39	93.17(19)
C16–S5–C14	92.3(2)	C39–S12–C33	93.25(19)
C15–S6–C14	92.4(2)	C41–S13–C40	93.44(19)
C16–S7–C22	103.9(2)	C42–S14–C40	93.34(19)
C15–S8–C17	101.3(2)	C43–S15–C41	101.61(19)
C4A–N1A–C3	115.5(10)	C48–S16–C42	103.8(2)
C4B–N1B–C3	125.0(11)	C30–N5–C39	122.0(5)
C11–N2–C10	122.0(6)	C36–N6–C37	122.4(4)
C19–N3–C20	122.2(4)	C46–N7–C45	122.3(4)
C25–N4–C24	123.4(5)	C50–N8–C51	121.8(5)
C2–C1–S1	125.9(4)	C28–C27–S9	117.5(4)
C2–C1–C5A	113.4(7)	C31–C27–S9	123.3(4)
C2–C1–C5B	124.2(8)	C31–C27–C28	119.2(5)
C5A–C1–S1	119.9(6)	C29–C28–C27	119.4(5)
C5B–C1–S1	108.5(8)	N5–C29–C28	120.0(5)
C3–C1–C1	119.5(5)	N5–C30–C31	121.0(5)
N1A–C3–C2	125.9(8)	C30–C31–C27	118.3(5)
C2–C3–N1B	114.6(8)	S11–C32–S9	116.5(2)
N1A–C4A–C5A	121.1(9)	C33–C32–S9	125.3(3)
N1B–C4B–C5B	122.6(12)	C33–C32–S11	117.7(3)
C4A–C5A–C1	123.1(9)	S10–C33–S12	119.0(2)
C4B–C5B–C1	112.0(12)	C32–C33–S10	124.5(3)
S1–C6–S3	118.9(2)	C32–C33–S12	116.3(3)
C7–C6–S1	122.9(3)	C35–C34–S10	117.4(3)
C7–C6–S3	117.1(3)	C35–C34–C38	118.6(4)
S2–C6–S4	118.1(2)	C38–C34–S10	124.0(3)
C6–C7–S2	124.3(4)	C36–C35–C34	119.4(4)
C6–C7–S4	117.0(3)	N6–C36–C35	120.0(4)
C9–C8–S2	124.1(4)	N6–C37–C38	120.2(4)
C9–C8–C12	117.5(6)	C37–C38–C34	119.4(4)

C12–C8–S2	118.4(5)	S12–C39–S11	113.7(2)
C10–C9–C8	120.8(6)	C40–C39–S11	121.8(3)
N2–C10–C9	120.7(8)	C40–C39–S12	124.4(3)
N2–C11–C12	122.1(6)	S14–C40–S13	113.9(2)
C8–C12–C11	116.8(7)	C39–C40–S13	121.3(3)
S3–C13–S4	113.5(2)	C39–C40–S14	124.8(3)
C14–C13–S3	123.9(3)	S15–C41–S13	118.2(2)
C14–C13–S4	122.6(3)	C42–C41–S13	116.7(3)
S6–C14–S5	113.3(2)	C42–C41–S15	124.7(3)
C13–C14–S5	124.2(3)	S14–C42–S16	119.2(2)
C13–C14–S6	122.3(3)	C41–C42–S14	117.7(3)
S6–C15–S8	117.9(2)	C41–C42–S16	122.0(3)
C16–C15–S6	117.7(3)	C44–C43–S15	124.2(3)
C16–C15–S8	124.1(3)	C44–C43–C47	119.1(4)
S7–C16–S5	120.7(2)	C47–C43–S15	116.7(3)
C15–C16–S5	116.5(3)	C45–C44–C43	119.2(4)
C15–C16–S7	121.9(3)	N7–C45–C44	120.2(5)
C18–C17–S8	116.2(3)	N7–C46–C47	120.2(4)
C18–C17–C21	119.8(4)	C46–C47–C43	118.9(4)
C21–C17–S8	124.0(3)	C49–C48–S16	125.8(4)
C19–C18–C17	118.8(4)	C49–C48–C52	119.0(5)
N3–C19–C18	120.1(4)	C52–C48–S16	115.2(4)
N3–C20–C21	120.4(4)	C50–C49–C48	119.2(5)
C20–C21–C17	118.6(4)	N8–C50–C49	120.1(6)
C23–C22–S7	116.8(4)	C52–C51–N8	119.8(7)
C23–C22–C26	119.5(5)	C51–C52–C48	119.8(7)
C26–C22–S7	123.6(4)		

Table S14. Structural and DFT-optimized[a] selected average distances (Å) for compounds **1–6**.

	Structural		Calcd.[a]	
	C=C	C–S	C=C	C–S
**1**	1.337	1.756	1.344	1.753
**2**	1.356	1.758	1.346[b]	1.756[b]
**3**	1.346	1.749	–	–
**4**	1.345	1.758	1.346[c]	1.753[c]
**5**	1.347	1.756	1.346[b]	1.356[b]
**6**	1.344	1.755	1.346[b]	1.356[b]

[a] mPW1PW/Def2TZVP (C, H, N, S)/LANL08(d) (halogens). [b] Calcd. for the $[H_4\mathbf{L_1}]^{4+}$ cation. [c] Calcd. for the $[(H_4\mathbf{L_1})(\mathbf{L_1})(IBr_2)]^{3+}$ cage.

Table S15. Selected optimized bond distances *d* (Å), sum of electronic and thermal enthalpies $E_0 + H_{corr}$, sum of electronic and thermal free energy $E_0 + G_{corr}$ (Hartree), unscaled Raman-active harmonic frequencies ν (a_{1g}/σ_g, cm^{-1}), and charges *Q* (|e|)[a–c] calculated on coordinated X_2/XY units and $[XY_2]^-$ anions,[d] calculated for I_2, $[ICl_2]^-$, $[IBr_2]^-$, **L**$_1$, $[H_4\mathbf{L_1}]^{4+}$, **L**$_1$·4I_2, and $[(H_4\mathbf{L_1})(\mathbf{L_1})(IBr_2)]^{3+}$ at hybrid-DFT level at the relevant optimized geometries in the gas phase (functional mPW1PW/Def2TZVP (C, H, N, S)/LANL08(d) (Cl, Br, I).

	$d_{N–X}$	$d_{X–Y}$	$E_0 + H_{corr}$	$E_0 + G_{corr}$	ν	Q_X			Q_Y		
						Q_H[a]	Q_{NBO}[b]	Q_B[c]	Q_H[a]	Q_{NBO}[b]	Q_B[c]
I_2	–	2.686	–22.8154	–22.8450	217.8	0.000	0.000	0.000	0.000	0.000	0.000
$[ICl_2]^{-}$ [d]	–	2.609	–41.47362	–41.5069	244.0	–0.084	+0.141	+0.210	–0.458	–0.570	–0.605
$[IBr_2]^{-}$ [d]	–	2.776	–37.91805	–37.95407	149.9	–0.133	+0.032	+0.091	–0.433	–0.516	–0.546
**L**$_1$	–	–	–4404.86715	–4404.98144	–	–	–	–	–	–	–
$[H_4\mathbf{L_1}]^{4+}$	–	–	–4405.98964	–4406.10294	–	–	–	–	–	–	–
**L**$_1$·4I_2	2.629	2.749	–4496.18161	–4496.35569	173.0	–0.044	+0.034	0.079	–0.187	–0.155	–0.179
$[(H_4\mathbf{L_1})(\mathbf{L_1})(IBr_2)]^{3+}$	–	–	–8849.21772	–8849.41910	–	–	–	–	–	–	–

[a] Hirshfeld charge. [b] Natural charge (calculated with NBO 3.1). [c] Bader charge. [d] $D_{\infty h}$ point group.

Table S16. Optimized geometry calculated for $\mathbf{L_1}$ ($\alpha_2\alpha_2$ conformation; Scheme S3) in orthogonal Cartesian coordinates format at hybrid-DFT level (functional mPW1PW//Def2TZVP). Total electronic energy E_0 = –4405.2684703 Hartree. Most negative vibrational frequency ν = –0.085 cm^{-1} (not an imaginary frequency).

Atom	x	y	z
C	-3.0318	-2.4371	1.2518
C	-4.0361	-2.7181	0.3369
C	-4.7710	-3.8866	0.5015
C	-4.4671	-4.7097	1.5725
N	-3.5030	-4.4594	2.4507
C	-2.8073	-3.3425	2.2764
S	-4.5057	-1.6531	-0.9888
C	-3.0924	-0.6757	-1.2664
S	-1.6382	-1.4603	-1.8525
C	-0.6721	-0.0001	-1.8126
S	-1.6383	1.4601	-1.8525
C	-3.0925	0.6755	-1.2664
C	0.6720	0.0000	-1.8126
S	1.6381	1.4602	-1.8525
C	3.0923	0.6757	-1.2664
C	3.0924	-0.6756	-1.2664
S	1.6382	-1.4602	-1.8525
S	4.5056	1.6530	-0.9888
C	4.0359	2.7181	0.3368
C	3.0315	2.4371	1.2516
C	2.8070	3.3426	2.2761
N	3.5027	4.4594	2.4506
C	4.4669	4.7097	1.5725
C	4.7709	3.8865	0.5015
S	4.5057	-1.6528	-0.9888
C	4.0361	-2.7180	0.3368
C	3.0319	-2.4370	1.2518
C	2.8074	-3.3425	2.2763
N	3.5030	-4.4594	2.4505
C	4.4672	-4.7097	1.5723
C	4.7711	-3.8865	0.5012
S	-4.5058	1.6527	-0.9888
C	-4.0361	2.7180	0.3366
C	-4.7711	3.8864	0.5012
C	-4.4671	4.7097	1.5721
N	-3.5026	4.4596	2.4501
C	-2.8069	3.3428	2.2757
C	-3.0315	2.4372	1.2513
H	2.4369	1.5378	1.1761
H	2.0228	3.1482	3.0009

H	5.0286	5.6258	1.7226
H	5.5586	4.1550	-0.1906
H	2.4374	-1.5376	1.1764
H	2.0234	-3.1481	3.0012
H	5.0288	-5.6259	1.7222
H	5.5585	-4.1550	-0.1909
H	-5.5588	4.1548	-0.1907
H	-5.0288	5.6258	1.7222
H	-2.0226	3.1486	3.0004
H	-2.4368	1.5379	1.1758
H	-5.5585	-4.1552	-0.1907
H	-5.0287	-5.6259	1.7226
H	-2.0233	-3.1481	3.0012
H	-2.4373	-1.5377	1.1764

Table S17. Optimized geometry calculated for **L**$_1$ ($\beta_2\alpha_2$ conformation; Scheme S3) in orthogonal Cartesian coordinates format at hybrid-DFT level (functional mPW1PW//Def2TZVP). Total electronic energy E_0 = –4405.2662972 Hartree. Most negative vibrational frequency ν = –1.343 cm^{-1} (not an imaginary frequency).

Atom	x	y	z
C	4.0190	2.4525	1.1060
C	4.6204	2.7287	-0.1133
C	5.3562	3.9018	-0.2359
C	5.4550	4.7347	0.8657
N	4.8766	4.4896	2.0358
C	4.1741	3.3675	2.1348
S	4.5825	1.6522	-1.5108
C	3.1661	0.6757	-1.2507
S	1.5966	1.4593	-1.2581
C	0.7229	-0.0001	-0.8339
S	1.5966	-1.4595	-1.2582
C	3.1660	-0.6759	-1.2508
C	-0.4910	-0.0001	-0.2586
S	-1.3463	-1.4723	0.1604
C	-2.7232	-0.6746	0.8897
C	-2.7232	0.6744	0.8898
S	-1.3463	1.4721	0.1606
S	-3.9039	-1.6532	1.7199
C	-4.5729	-2.6773	0.4477
C	-4.5716	-2.3567	-0.9022
C	-5.1818	-3.2337	-1.7846
N	-5.7850	-4.3587	-1.4204
C	-5.7871	-4.6466	-0.1240
C	-5.1954	-3.8550	0.8457
S	-3.9039	1.6531	1.7199
C	-4.5721	2.6777	0.4477
C	-4.5713	2.3567	-0.9021
C	-5.1809	3.2341	-1.7846
N	-5.7830	4.3597	-1.4206
C	-5.7846	4.6480	-0.1243
C	-5.1934	3.8560	0.8455
S	4.5825	-1.6525	-1.5109
C	4.6204	-2.7290	-0.1134
C	5.3561	-3.9021	-0.2361
C	5.4549	-4.7350	0.8656
N	4.8766	-4.4900	2.0356
C	4.1741	-3.3679	2.1347
C	4.0189	-2.4528	1.1059
H	-4.1103	-1.4496	-1.2659
H	-5.1871	-3.0078	-2.8460

H	-6.2812	-5.5689	0.1634
H	-5.2145	-4.1546	1.8854
H	-4.1109	1.4491	-1.2656
H	-5.1866	3.0080	-2.8460
H	-6.2779	5.5708	0.1630
H	-5.2121	4.1560	1.8852
H	5.8366	-4.1670	-1.1690
H	6.0256	-5.6551	0.7941
H	3.7073	-3.1771	3.0957
H	3.4439	-1.5507	1.2590
H	5.8367	4.1667	-1.1688
H	6.0257	5.6548	0.7943
H	3.7073	3.1766	3.0958
H	3.4439	1.5503	1.2591

Table S18. Optimized geometry calculated for $\mathbf{L_1}$ ($\beta\alpha\alpha_2$ and $\alpha\beta\alpha_2$ conformations; Scheme S3) in orthogonal Cartesian coordinates format at hybrid-DFT level (functional mPW1PW//Def2TZVP). Total electronic energy E_0 = –4405.2681319 Hartree. Most negative vibrational frequency ν = –1.172 cm^{-1} (not an imaginary frequency).

Atom	x	y	z
C	-3.5733	-2.2867	1.8453
C	-4.3458	-2.9277	0.8876
C	-5.0551	-4.0628	1.2631
C	-4.9580	-4.4954	2.5748
N	-4.2148	-3.8992	3.4999
C	-3.5405	-2.8193	3.1240
S	-4.5564	-2.3585	-0.7691
C	-3.1475	-1.3787	-1.0591
S	-1.5765	-2.1576	-1.0640
C	-0.6900	-0.6635	-1.2854
S	-1.6643	0.6091	-1.9916
C	-3.1884	-0.0984	-1.4895
C	0.6134	-0.5252	-0.9890
S	1.5143	0.9516	-1.2716
C	2.8402	0.5181	-0.2131
C	2.8833	-0.7632	0.2084
S	1.5976	-1.8182	-0.3334
S	4.1267	1.6656	0.0223
C	3.3372	3.0158	0.8397
C	2.2003	2.8926	1.6256
C	1.7178	4.0263	2.2592
N	2.2831	5.2246	2.1765
C	3.3762	5.3237	1.4294
C	3.9394	4.2641	0.7379
S	4.0980	-1.4579	1.2523
C	5.4146	-1.8822	0.1576
C	5.3840	-1.7342	-1.2210
C	6.5009	-2.1207	-1.9458
N	7.6041	-2.6250	-1.4082
C	7.6161	-2.7616	-0.0866
C	6.5623	-2.4118	0.7387
S	-4.6573	0.7720	-1.8262
C	-4.5225	2.2344	-0.8485
C	-5.3093	3.3189	-1.2192
C	-5.2727	4.4577	-0.4325
N	-4.5188	4.5858	0.6531
C	-3.7697	3.5432	0.9903
C	-3.7368	2.3486	0.2892
H	1.7019	1.9414	1.7503
H	0.8268	3.9579	2.8751

H	3.8292	6.3078	1.3672
H	4.8222	4.4126	0.1297
H	4.5176	-1.3359	-1.7301
H	6.4978	-2.0150	-3.0260
H	8.5218	-3.1738	0.3463
H	6.6385	-2.5471	1.8100
H	-5.9323	3.2826	-2.1034
H	-5.8800	5.3159	-0.7008
H	-3.1589	3.6594	1.8799
H	-3.1143	1.5325	0.6278
H	-5.6647	-4.6036	0.5508
H	-5.5042	-5.3778	2.8914
H	-2.9380	-2.3395	3.8885
H	-3.0101	-1.3948	1.6100

Table S19. Optimized geometry calculated for **L_1** (βαβα conformation; Scheme S3) in orthogonal Cartesian coordinates format at hybrid-DFT level (functional mPW1PW//Def2TZVP). Total electronic energy E_0 = –4405.2675238 Hartree. Most negative vibrational frequency ν = –0.004 cm^{-1} (not an imaginary frequency).

Atom	x	y	z
C	3.0903	3.0452	0.7596
C	4.0495	2.7306	-0.1925
C	4.7387	3.7695	-0.8065
C	4.4359	5.0688	-0.4349
N	3.5137	5.3869	0.4657
C	2.8614	4.3828	1.0392
S	4.5181	1.0834	-0.6188
C	3.1038	0.1428	-0.2427
S	1.6367	0.4329	-1.1545
C	0.6718	-0.7113	-0.2416
S	1.6384	-1.8830	0.6321
C	3.1064	-0.9336	0.5713
C	-0.6718	-0.7113	-0.2416
S	-1.6382	-1.8831	0.6321
C	-3.1063	-0.9337	0.5714
C	-3.1038	0.1427	-0.2426
S	-1.6368	0.4328	-1.1544
S	-4.4362	-1.4578	1.5735
C	-5.4593	-2.3894	0.4794
C	-5.1589	-2.6743	-0.8442
C	-6.0657	-3.4277	-1.5738
N	-7.2082	-3.8979	-1.0901
C	-7.4797	-3.6175	0.1800
C	-6.6521	-2.8762	1.0045
S	-4.5182	1.0832	-0.6185
C	-4.0494	2.7305	-0.1926
C	-3.0900	3.0452	0.7592
C	-2.8610	4.3828	1.0386
N	-3.5134	5.3869	0.4651
C	-4.4357	5.0687	-0.4354
C	-4.7386	3.7693	-0.8067
S	4.4363	-1.4576	1.5733
C	5.4592	-2.3896	0.4792
C	6.6519	-2.8767	1.0043
C	7.4793	-3.6183	0.1799
N	7.2077	-3.8986	-1.0902
C	6.0653	-3.4281	-1.5739
C	5.1588	-2.6743	-0.8443
H	-4.2438	-2.3301	-1.3053
H	-5.8499	-3.6634	-2.6109

H	-8.4162	-4.0043	0.5685
H	-6.9352	-2.6813	2.0309
H	-2.5357	2.2750	1.2772
H	-2.1116	4.6559	1.7745
H	-4.9616	5.8968	-0.8992
H	-5.4891	3.5747	-1.5617
H	6.9350	-2.6819	2.0307
H	8.4156	-4.0054	0.5684
H	5.8495	-3.6637	-2.6111
H	4.2437	-2.3299	-1.3055
H	5.4891	3.5750	-1.5617
H	4.9617	5.8969	-0.8987
H	2.1121	4.6558	1.7753
H	2.5360	2.2750	1.2775

Table S20. Optimized geometry calculated for **L_1** (βααβ conformation; Scheme S3) in orthogonal Cartesian coordinates format at hybrid-DFT level (functional mPW1PW//Def2TZVP). Total electronic energy E_0 = –4405.2676785 Hartree. Most negative vibrational frequency ν = –0.316 cm^{-1} (not an imaginary frequency).

Atom	x	y	z
C	-5.1609	-1.3048	-2.2736
C	-5.4313	-1.8891	-1.0453
C	-6.6136	-2.6083	-0.9031
C	-7.4613	-2.7026	-1.9926
N	-7.2188	-2.1473	-3.1750
C	-6.0858	-1.4670	-3.2936
S	-4.3801	-1.8082	0.3691
C	-3.0825	-0.7497	-0.1232
S	-1.6013	-1.5036	-0.6701
C	-0.6716	-0.0184	-0.6555
S	-1.6735	1.4195	-0.6830
C	-3.1134	0.5996	-0.1149
C	0.6714	0.0185	-0.6556
S	1.6011	1.5036	-0.6710
C	3.0823	0.7499	-0.1237
C	3.1132	-0.5993	-0.1146
S	1.6733	-1.4195	-0.6824
S	4.3799	1.8088	0.3681
C	5.4316	1.8882	-1.0460
C	5.1612	1.3034	-2.2741
C	6.0865	1.4646	-3.2939
N	7.2198	2.1443	-3.1754
C	7.4623	2.7000	-1.9931
C	6.6143	2.6068	-0.9039
S	4.5470	-1.5389	0.1800
C	4.0778	-2.6113	1.5010
C	3.0922	-2.3144	2.4316
C	2.8628	-3.2222	3.4528
N	3.5384	-4.3544	3.6100
C	4.4862	-4.6180	2.7183
C	4.7922	-3.7942	1.6479
S	-4.5472	1.5394	0.1792
C	-4.0781	2.6123	1.4998
C	-4.7925	3.7953	1.6461
C	-4.4865	4.6195	2.7162
N	-3.5388	4.3563	3.6081
C	-2.8632	3.2241	3.4514
C	-3.0925	2.3159	2.4305
H	4.2549	0.7401	-2.4473
H	5.8944	1.0162	-4.2634

H	8.3914	3.2534	-1.9031
H	6.8744	3.0811	0.0337
H	2.5173	-1.4009	2.3722
H	2.0928	-3.0165	4.1893
H	5.0312	-5.5462	2.8552
H	5.5641	-4.0739	0.9428
H	-5.5644	4.0747	0.9409
H	-5.0316	5.5478	2.8527
H	-2.0932	3.0187	4.1880
H	-2.5176	1.4023	2.3716
H	-6.8736	-3.0823	0.0346
H	-8.3901	-3.2564	-1.9025
H	-5.8937	-1.0191	-4.2633
H	-4.2549	-0.7410	-2.4469

Table S21. Optimized geometry calculated for $[H_4\mathbf{L_1}]^{4+}$ ($\alpha_2\alpha_2$ conformation) in orthogonal Cartesian coordinates format at hybrid-DFT level (functional mPW1PW//Def2TZVP). Total electronic energy E_0 = –4406.4467994 Hartree. Most negative vibrational frequency ν = –0.930 cm^{-1} (not an imaginary frequency).

Atom	x	y	z
C	1.8930	-0.4713	0.4613
C	0.9586	-0.9866	1.3652
C	1.2782	-2.1582	2.0704
C	2.4864	-2.7639	1.8623
N	3.3557	-2.2385	0.9826
C	3.0870	-1.1232	0.2889
S	-0.5961	-0.3095	1.7579
C	-0.8994	1.0179	0.6506
S	-1.1318	0.6902	-1.0595
C	-2.0629	2.1465	-1.3672
S	-1.8691	3.3672	-0.1203
C	-1.2398	2.2543	1.0843
C	-2.8840	2.2910	-2.4236
S	-3.8689	3.7192	-2.6933
C	-5.0079	2.9176	-3.7636
C	-4.6676	1.6813	-4.1974
S	-3.1319	1.0422	-3.6326
S	-6.6346	3.5534	-3.9365
C	-6.5206	5.1022	-4.7227
C	-5.3754	5.6734	-5.2869
C	-5.4687	6.9002	-5.8922
N	-6.6450	7.5412	-5.9457
C	-7.7683	7.0254	-5.4186
C	-7.7326	5.8073	-4.7984
S	-5.8247	0.6109	-4.9692
C	-5.1405	0.0881	-6.4819
C	-3.9687	0.5624	-7.0803
C	-3.5844	0.0538	-8.2942
N	-4.3262	-0.8836	-8.9011
C	-5.4594	-1.3633	-8.3612
C	-5.8877	-0.8956	-7.1498
S	-1.4062	2.6333	2.7898
C	-0.4197	4.0278	3.1244
C	-0.5642	4.5455	4.4218
C	0.1813	5.6257	4.8054
N	1.0413	6.1870	3.9387
C	1.2064	5.7236	2.6916
C	0.4887	4.6399	2.2549
H	-4.4179	5.1748	-5.2619

H	-4.6234	7.3965	-6.3479
H	-8.6702	7.6152	-5.5090
H	-8.6444	5.4092	-4.3732
H	-3.3573	1.3208	-6.6145
H	-2.6915	0.3771	-8.8108
H	-5.9910	-2.1187	-8.9234
H	-6.7984	-1.2972	-6.7256
H	-1.2571	4.1095	5.1292
H	0.1151	6.0687	5.7896
H	1.9244	6.2417	2.0715
H	0.6438	4.2822	1.2479
H	0.5867	-2.5978	2.7770
H	2.7898	-3.6665	2.3748
H	3.8521	-0.7781	-0.3923
H	1.7027	0.4283	-0.1049
H	1.5839	6.9880	4.2400
H	4.2453	-2.7022	0.8400
H	-6.6902	8.4460	-6.3996
H	-4.0234	-1.2434	-9.7987

Table S22. Optimized geometry calculated for the adduct $\mathbf{L_1}\cdot 4I_2$ (converged to $\alpha_2\alpha_2$ conformation) in orthogonal Cartesian coordinates format at hybrid-DFT level (functional mPW1PW//Def2TZVP (C, H, N, S)/LANL08(d) (I)). Total electronic energy E_0 = –4496.6063418 Hartree. Most negative vibrational frequency ν = –0.549 cm^{-1} (waving mode with TTF deform.).

Atom	x	y	z
C	2.4298	3.3875	1.9212
C	2.6770	4.3135	2.9261
C	3.7869	5.1453	2.8066
C	4.5904	5.0190	1.6910
N	4.3557	4.1295	0.7328
C	3.3016	3.3318	0.8494
S	1.6451	4.5775	4.3263
C	0.6751	3.1342	4.4332
S	1.4636	1.6347	4.8727
C	0.0000	0.6721	4.8045
S	-1.4641	1.6342	4.8728
C	-0.6762	3.1340	4.4333
C	0.0002	-0.6724	4.8046
S	-1.4635	-1.6350	4.8727
C	-0.6750	-3.1345	4.4332
C	0.6763	-3.1342	4.4332
S	1.4642	-1.6344	4.8729
S	-1.6449	-4.5777	4.3263
C	-2.6770	-4.3136	2.9262
C	-2.4299	-3.3873	1.9214
C	-3.3017	-3.3315	0.8497
N	-4.3557	-4.1293	0.7330
C	-4.5903	-5.0190	1.6910
C	-3.7867	-5.1455	2.8065
S	1.6468	-4.5772	4.3266
C	2.6801	-4.3122	2.9277
C	2.4334	-3.3862	1.9227
C	3.3064	-3.3296	0.8519
N	4.3612	-4.1265	0.7364
C	4.5954	-5.0160	1.6947
C	3.7907	-5.1432	2.8093
S	-1.6466	4.5769	4.3267
C	-2.6802	4.3118	2.9279
C	-3.7906	5.1430	2.8093
C	-4.5954	5.0157	1.6948
N	-4.3614	4.1258	0.7367
C	-3.3068	3.3287	0.8524
C	-2.4338	3.3853	1.9232
I	5.9278	4.0164	-1.3708

I	7.5718	3.9209	-3.5725
I	5.9350	-4.0109	-1.3658
I	7.5804	-3.9129	-3.5664
I	-5.9271	-4.0161	-1.3712
I	-7.5694	-3.9218	-3.5742
I	-5.9359	4.0104	-1.3651
I	-7.5823	3.9129	-3.5648
H	-1.5778	-2.7245	1.9616
H	-3.1448	-2.6229	0.0448
H	-5.4610	-5.6504	1.5597
H	-4.0249	-5.8769	3.5673
H	1.5808	-2.7240	1.9619
H	3.1499	-2.6211	0.0469
H	5.4668	-5.6467	1.5643
H	4.0287	-5.8744	3.5703
H	-4.0282	5.8745	3.5701
H	-5.4665	5.6466	1.5643
H	-3.1505	2.6198	0.0477
H	-1.5813	2.7230	1.9626
H	4.0251	5.8766	3.5674
H	5.4611	5.6503	1.5598
H	3.1447	2.6233	0.0444
H	1.5777	2.7247	1.9612

Table S23. Optimized geometry calculated for the adduct **L**$_1$·4I$_2$ (converged to $\alpha_2\alpha_2$ conformation) in orthogonal Cartesian coordinates format at hybrid-DFT level (functional mPW1PW//Def2TZVP). Total electronic energy E_0 = –6788.0183 Hartree. Most negative vibrational frequency ν = –0.712 cm^{-1} (waving).

Atom	x	y	z
C	-0.0100	-0.0392	-0.0327
C	0.0012	-0.0241	1.3557
C	1.2291	-0.0345	2.0112
C	2.3828	-0.0536	1.2524
N	2.3726	-0.0742	-0.0757
C	1.2012	-0.0700	-0.6995
S	-1.4324	0.0833	2.3708
C	-2.7400	-0.4110	1.3315
S	-2.8018	-2.0812	0.8104
C	-4.2616	-1.8839	-0.1397
S	-5.1751	-0.4504	0.2882
C	-3.8359	0.3420	1.0904
C	-4.6875	-2.7909	-1.0361
S	-6.2112	-2.6566	-1.8922
C	-5.8184	-3.8794	-3.0819
C	-4.7225	-4.6324	-2.8407
S	-3.8379	-4.2873	-1.3700
S	-6.9977	-4.2473	-4.3100
C	-7.0661	-2.7961	-5.3033
C	-6.0791	-1.8205	-5.3502
C	-6.2442	-0.7600	-6.2223
N	-7.2948	-0.6369	-7.0238
C	-8.2357	-1.5736	-6.9829
C	-8.1713	-2.6650	-6.1391
S	-4.3277	-6.0819	-3.7225
C	-2.7071	-5.7914	-4.3438
C	-2.1229	-4.5390	-4.4794
C	-0.8598	-4.4597	-5.0371
N	-0.1854	-5.5220	-5.4589
C	-0.7472	-6.7192	-5.3345
C	-1.9981	-6.9068	-4.7803
S	-4.1024	1.9179	1.7833
C	-4.3579	2.9709	0.3967
C	-4.9443	4.2069	0.6530
C	-5.1060	5.0915	-0.3951
N	-4.7371	4.8104	-1.6398
C	-4.1832	3.6294	-1.8841
C	-3.9662	2.6790	-0.9031
I	4.6570	-0.0724	-1.4266
I	7.0034	-0.0553	-2.7942

I	2.2105	-5.2830	-6.5750
I	4.6566	-5.0539	-7.7330
I	-7.5109	1.3970	-8.7150
I	-7.7399	3.4640	-10.4619
I	-5.0650	6.6069	-3.5655
I	-5.3939	8.4621	-5.5218
H	-5.1970	-1.8798	-4.7293
H	-5.4967	0.0223	-6.2872
H	-9.0790	-1.4391	-7.6499
H	-8.9698	-3.3951	-6.1327
H	-2.6343	-3.6407	-4.1653
H	-0.3696	-3.5007	-5.1587
H	-0.1667	-7.5632	-5.6882
H	-2.4081	-7.9038	-4.6884
H	-5.2732	4.4768	1.6478
H	-5.5558	6.0641	-0.2337
H	-3.8927	3.4387	-2.9109
H	-3.4981	1.7382	-1.1537
H	1.2888	-0.0316	3.0915
H	3.3570	-0.0593	1.7271
H	1.2344	-0.0840	-1.7828
H	-0.9354	-0.0226	-0.5898

Table S24. Optimized geometry calculated for the fragment $[(H_4\mathbf{L_1})(\mathbf{L_1})(IBr_2)]^{3+}$ in orthogonal Cartesian coordinates format at hybrid-DFT level (functional mPW1PW//Def2TZVP (C, H, N, S)/LANL08(d) (I,Br)). Total electronic energy E_0 = –8850.081946 Hartree. Most negative vibrational frequency ν = –0.001 cm^{-1} (not an imaginary frequency).

**Atom**	**x**	**y**	**z**
I	10.2526	9.2589	11.0896
Br	10.2520	6.4496	11.0902
Br	10.2533	11.9354	11.0888
S	11.7168	2.9448	9.4499
S	11.9123	3.8390	6.5880
C	10.2538	2.9570	10.4182
N	13.7098	7.9218	7.0980
H	14.0632	8.9545	7.2080
C	12.4091	6.1747	8.0191
H	11.8101	5.8255	8.8479
C	10.9305	3.5405	8.0078
C	12.6132	5.4119	6.8695
C	13.4129	5.9424	5.8481
H	13.6121	5.3858	4.9423
C	12.9775	7.4196	8.0971
H	12.8461	8.0492	8.9660
C	13.9349	7.2036	5.9911
H	14.5368	7.6681	5.2222
S	8.7906	2.9441	12.7317
S	8.5950	3.8383	15.5935
C	10.2535	2.9571	11.7631
N	6.7973	7.9209	15.0840
H	6.4437	8.9529	14.9740
C	8.0984	6.1743	14.1630
H	8.6977	5.8253	13.3343
C	9.5768	3.5403	14.1737
C	7.8940	5.4112	15.3123
C	7.0940	5.9414	16.3336
H	6.8945	5.3845	17.2392
C	7.5299	7.4191	14.0850
H	7.6615	8.0489	13.2163
C	6.5718	7.2025	16.1908
H	5.9696	7.6668	16.9595
S	11.7160	2.9451	12.7322
S	11.9101	3.8392	15.5941
N	13.7074	7.9220	15.0850
H	14.0612	8.9539	14.9751
C	12.4047	6.1762	14.1646
H	11.8039	5.8282	13.3365

C	10.9289	3.5407	14.1739
C	12.6107	5.4122	15.3130
C	13.4122	5.9417	16.3336
H	13.6129	5.3841	17.2384
C	12.9731	7.4211	14.0867
H	12.8404	8.0516	13.2187
C	13.9343	7.2028	16.1909
H	14.5377	7.6665	16.9591
S	8.7915	2.9441	9.4490
S	8.5973	3.8377	6.5869
N	6.7990	7.9203	7.0943
H	6.4454	8.9521	7.2036
C	8.1010	6.1747	8.0160
H	8.7010	5.8265	8.8446
C	9.5784	3.5401	8.0073
C	7.8960	5.4106	6.8674
C	7.0954	5.9400	5.8461
H	6.8956	5.3823	4.9410
C	7.5323	7.4195	8.0933
H	7.6643	8.0501	8.9614
C	6.5731	7.2011	5.9882
H	5.9704	7.6647	5.2195
S	14.7776	14.6204	9.4328
S	11.8964	15.4581	9.6279
N	14.3041	10.4889	7.6117
C	15.5523	12.4945	7.9680
H	16.4694	13.0422	7.7958
C	13.3547	14.8962	10.4143
C	14.5159	13.0357	8.7316
C	15.4054	11.2272	7.4441
H	16.2084	10.7832	6.8684
C	10.9261	15.4249	11.0893
C	13.3640	12.2757	8.8978
H	12.5198	12.6296	9.4701
C	13.3079	11.0291	8.3153
H	12.4082	10.4345	8.4277
S	5.7279	14.6191	12.7459
S	8.6089	15.4577	12.5505
N	6.2021	10.4886	14.5693
C	4.9529	12.4931	14.2103
H	4.0351	13.0402	14.3808
C	7.1507	14.8951	11.7644
C	5.9900	13.0348	13.4479
C	5.1001	11.2262	14.7349

H	4.2966	10.7819	15.3097
C	9.5791	15.4247	11.0890
C	7.1428	12.2756	13.2837
H	7.9874	12.6301	12.7125
C	7.1989	11.0294	13.8668
H	8.0993	10.4354	13.7561
S	14.7769	14.6205	12.7478
S	11.8956	15.4584	12.5512
N	14.3028	10.4898	14.5707
C	15.5521	12.4943	14.2117
H	16.4700	13.0412	14.3820
C	13.3544	14.8963	11.7658
C	14.5149	13.0361	13.4496
C	15.4050	11.2273	14.7362
H	16.2085	10.7828	15.3107
C	13.3620	12.2770	13.2856
H	12.5172	12.6317	12.7147
C	13.3059	11.0307	13.8686
H	12.4055	10.4369	13.7580
S	5.7284	14.6189	9.4310
S	8.6094	15.4576	9.6273
N	6.2037	10.4886	7.6074
C	4.9538	12.4927	7.9669
H	4.0358	13.0394	7.7966
C	7.1509	14.8951	10.4129
C	5.9908	13.0346	8.7291
C	5.1013	11.2258	7.4421
H	4.2979	10.7813	6.8674
C	7.1439	12.2759	8.8930
H	7.9886	12.6306	9.4639
C	7.2004	11.0297	8.3097
H	8.1010	10.4361	8.4201

Table S25. Optimized geometry calculated for the fragment $[(H_4\mathbf{L_1})(\mathbf{L_1})]^{4+}$ in orthogonal Cartesian coordinates format at hybrid-DFT level (functional mPW1PW//Def2TZVP (C, H, N, S)/LANL08(d) (I,Br)). Total electronic energy E_0 = –8811.8151101 Hartree. Most negative vibrational frequency ν = –0.002 cm^{-1}.

Atom	x	y	z
S	11.7157	3.1785	9.4455
S	11.8976	3.9074	6.5262
C	10.2527	3.2300	10.4164
N	13.8720	7.9325	6.8049
H	14.2375	8.9660	6.8861
C	12.6173	6.2397	7.8760
H	12.1110	5.8931	8.7637
C	10.9293	3.7094	7.9751
C	12.6711	5.4592	6.7191
C	13.3864	5.9510	5.6190
H	13.4761	5.3792	4.7048
C	13.2362	7.4614	7.8810
H	13.2410	8.1045	8.7497
C	13.9620	7.1967	5.6903
H	14.4966	7.6324	4.8570
S	8.7896	3.1784	12.7345
S	8.6076	3.9073	15.6538
C	10.2527	3.2300	11.7636
N	6.6328	7.9322	15.3751
H	6.2671	8.9656	15.2939
C	7.8877	6.2396	14.3040
H	8.3941	5.8930	13.4163
C	9.5760	3.7093	14.2049
C	7.8339	5.4590	15.4609
C	7.1185	5.9507	16.5609
H	7.0288	5.3788	17.4751
C	7.2687	7.4612	14.2990
H	7.2638	8.1043	13.4303
C	6.5428	7.1964	16.4897
H	6.0081	7.6319	17.3229
S	11.7157	3.1786	12.7345
S	11.8977	3.9074	15.6538
N	13.8719	7.9326	15.3753
H	14.2375	8.9660	15.2940
C	12.6172	6.2398	14.3040
H	12.1109	5.8932	13.4163
C	10.9293	3.7094	14.2049
C	12.6711	5.4592	15.4609
C	13.3864	5.9510	16.5610

H	13.4762	5.3791	17.4752
C	13.2361	7.4615	14.2991
H	13.2409	8.1047	13.4305
C	13.9620	7.1967	16.4898
H	14.4966	7.6324	17.3231
S	8.7896	3.1784	9.4455
S	8.6076	3.9072	6.5262
N	6.6328	7.9322	6.8049
H	6.2671	8.9655	6.8862
C	7.8877	6.2395	7.8761
H	8.3941	5.8930	8.7637
C	9.5760	3.7093	7.9751
C	7.8339	5.4590	6.7191
C	7.1185	5.9507	5.6191
H	7.0288	5.3788	4.7049
C	7.2687	7.4611	7.8810
H	7.2638	8.1043	8.7497
C	6.5428	7.1964	5.6903
H	6.0081	7.6319	4.8571
S	14.8006	14.5002	9.4464
S	11.8952	15.2770	9.6282
N	14.4306	10.4853	7.3606
C	15.6618	12.4475	7.9419
H	16.5960	12.9906	7.8816
C	13.3585	14.7224	10.4136
C	14.5700	12.9602	8.6461
C	15.5504	11.2149	7.3329
H	16.3963	10.7928	6.8043
C	10.9251	15.2311	11.0900
C	13.3965	12.2105	8.6596
H	12.5073	12.5667	9.1582
C	13.3797	11.0029	7.9985
H	12.4647	10.4197	7.9737
S	5.7034	14.4999	12.7337
S	8.6088	15.2769	12.5518
N	6.0739	10.4849	14.8194
C	4.8425	12.4470	14.2381
H	3.9083	12.9900	14.2984
C	7.1456	14.7222	11.7664
C	5.9342	12.9599	13.5340
C	4.9540	11.2144	14.8471
H	4.1081	10.7922	15.3756
C	9.5789	15.2310	11.0900
C	7.1078	12.2103	13.5205

H	7.9970	12.5666	13.0219
C	7.1248	11.0026	14.1816
H	8.0398	10.4196	14.2063
S	14.8006	14.5002	12.7337
S	11.8952	15.2770	12.5519
N	14.4306	10.4853	14.8195
C	15.6618	12.4476	14.2382
H	16.5959	12.9906	14.2985
C	13.3585	14.7224	11.7664
C	14.5700	12.9602	13.5340
C	15.5504	11.2150	14.8472
H	16.3963	10.7928	15.3758
C	13.3965	12.2105	13.5205
H	12.5073	12.5667	13.0219
C	13.3796	11.0029	14.1817
H	12.4646	10.4198	14.2064
S	5.7034	14.4999	9.4463
S	8.6088	15.2769	9.6282
N	6.0739	10.4849	7.3606
C	4.8425	12.4470	7.9419
H	3.9083	12.9900	7.8816
C	7.1456	14.7222	10.4136
C	5.9342	12.9599	8.6460
C	4.9540	11.2144	7.3330
H	4.1081	10.7922	6.8044
C	7.1078	12.2103	8.6595
H	7.9970	12.5666	9.1581
C	7.1248	11.0026	7.9985
H	8.0398	10.4196	7.9737

Table S26. Basis set effect on the DFT-optimized (mPW1PW functional) N···I and I–I bond distances *d* (Å) and the N–I–I angle α (°), compared to the mean relevant structural values determined for compound **1**.

	d(N···I)	*d*(I–I)	α(N–I–I)	Δ*d*(N···I)	Δ*d*(I–I)	Δα
Struct. (**1**)	2.48(9)	2.78(3)	176.7(3)	–	–	–
Def2tzvp/LANL08(d)	2.629	2.749	179.52	0.149	-0.031	2.82
Def2tzvp	2.654	2.716	179.51	0.174	-0.064	2.81

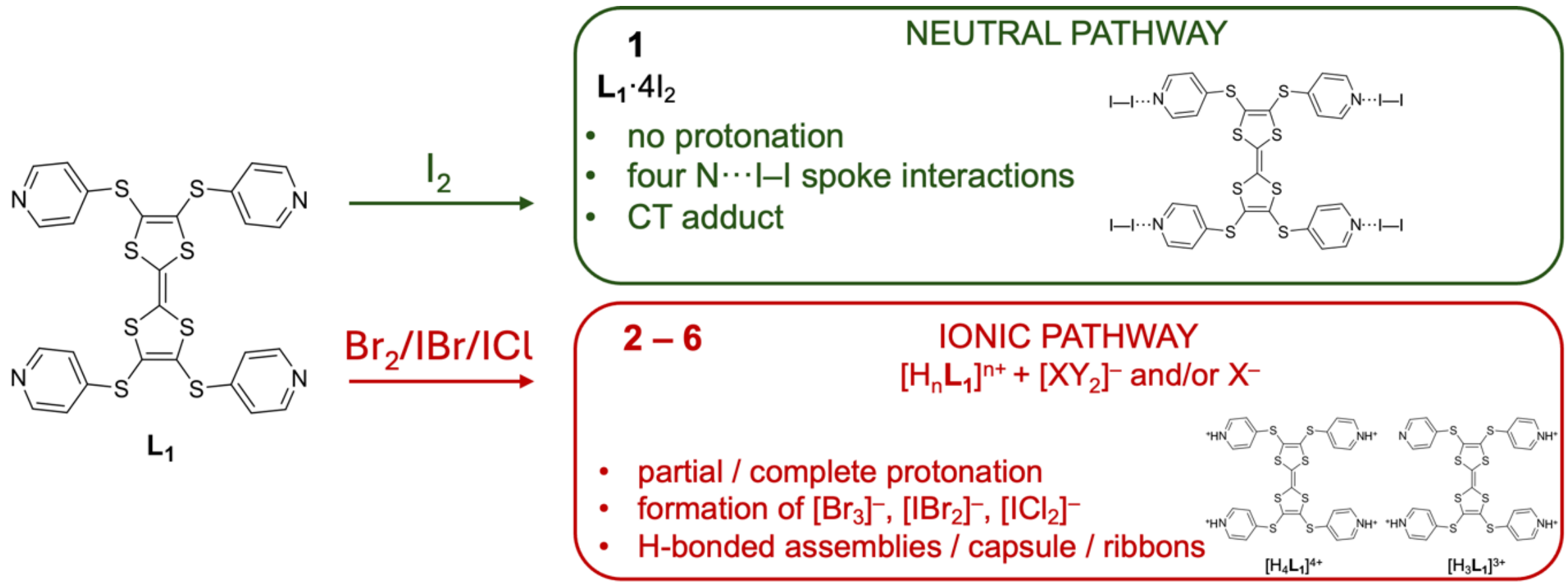

Compound	Halogen reagent	Product nature	Ligand protonation state	Halogen-containing species	Key structural feature	Interaction regime
1	I_2	Neutral	L_1	4 × I_2	Four independent N···I–I "spoke" interactions; 1:4 adduct	CT + electrostatic (halogen bonding)
2	Br_2	Ionic	$[H_4L_1]^{4+}$	$[Br_3]^-$, Br^-	Hydrogen-bonded dimeric boxes encapsulating tribromide anions	Ionic / electrostatic
3	Br_2	Ionic	$[H_3L_1]^{3+}$	$[Br_3]^-$, Br^-, Br_2	Capsule-like assemblies containing bromide, tribromide, and neutral Br_2	Ionic / electrostatic
4	IBr	Ionic	$[H_4L_1]^{4+}$ + L_1	$[IBr_2]^-$	Unprecedented tetracationic capsule $[(H_4L_1)(L_1)]^{4+}$ templated by $[IBr_2]^-$	Ionic (Coulombic inclusion)
5	ICl	Ionic	$[H_4L_1]^{4+}$	$[ICl_2]^-$, Cl^-	One-dimensional H-bonded ribbons involving $[ICl_2]^-$, anions	Ionic / electrostatic
6	ICl	Ionic	$[H_4L_1]^{4+}$ + $[H_3L_1]^{3+}$	$[ICl_2]^-$, Cl^-	One-dimensional H-bonded ribbons with mixed protonation states	Ionic / electrostatic

Scheme S1. Reactivity of tetra(pyridin-4-ylthio)tetrathiafulvalene (**L$_1$**) toward dihalogens and interhalogens (top). The reaction with diiodine selectively afforded the neutral "spoke" charge-transfer adduct **L$_1$**·4I$_2$ (**1**), in which all four pyridyl groups engage in independent N···I–I interactions (neutral pathway). The reactions of **L$_1$** with the more oxidizing reagents Br_2, IBr, and ICl led to ionic products (**2**–**6**) featuring partially or fully protonated ligands and discrete tri(inter)halide anions $[Br_3]^-$, $[IBr_2]^-$, $[ICl_2]^-$ (ionic pathway).

Summary of the products obtained from the reactions of **L$_1$** with dihalogens and interhalogens, highlighting the nature of the products, the protonation state of the ligand, the halogen-containing species, and the main structural features (bottom).

Py-derivatives used for the preparation of 1:1 CT "spoke" adducts

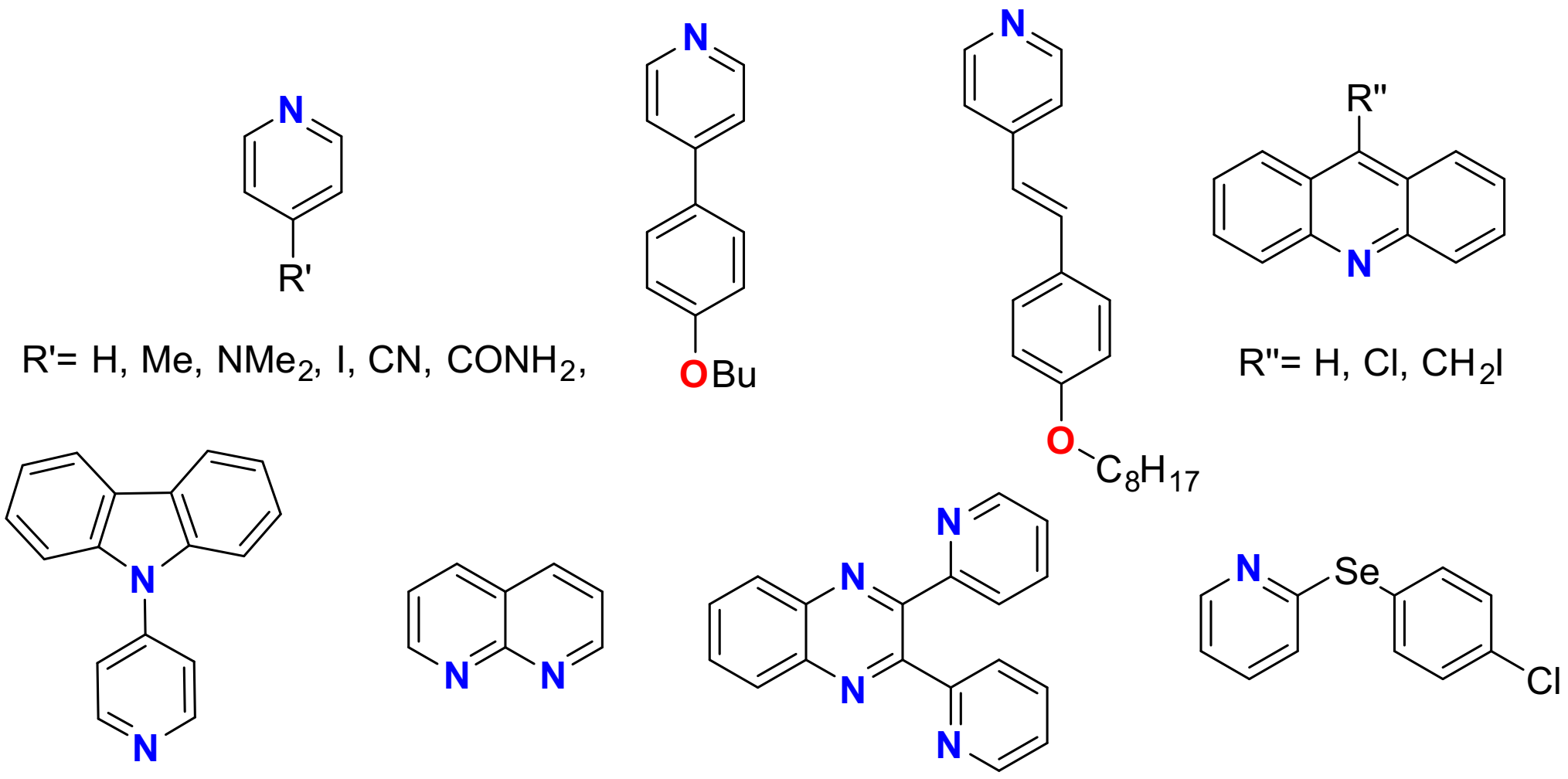

Py-derivatives used for the preparation of 1:2 (L/X_2) CT "spoke" adducts

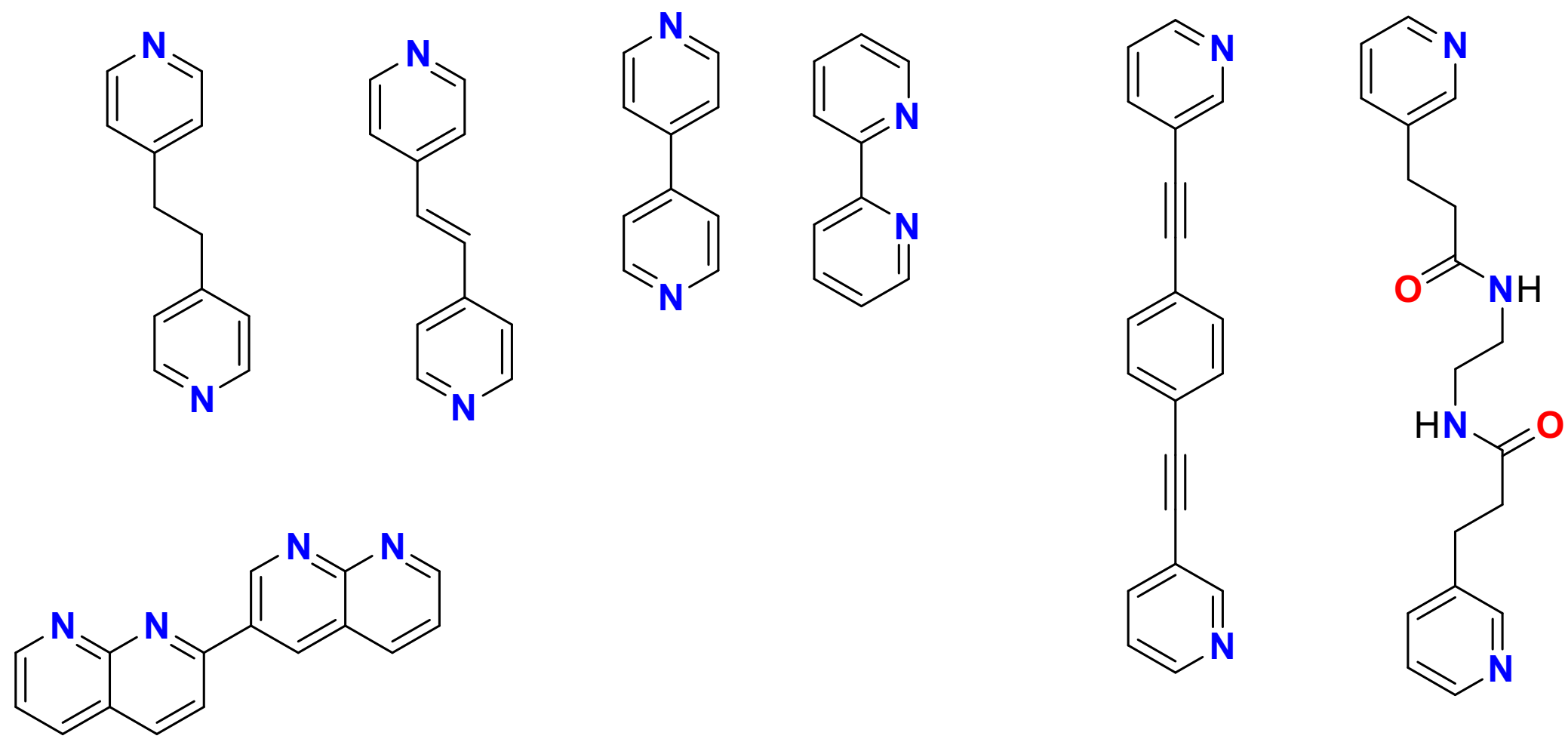

Py-derivatives used for the preparation of 1:4 (L/X_2) CT "spoke" adducts

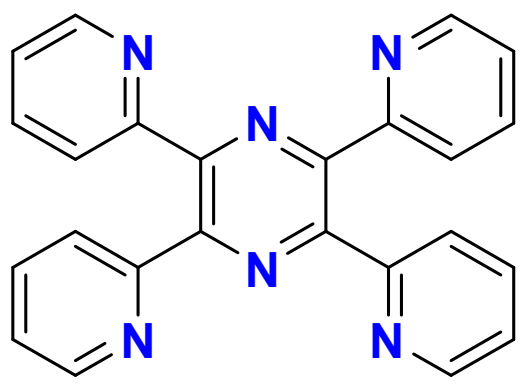

Scheme S2. Pyridine (Py) derivatives leading to CT “spoke” η^1-adducts with I_2 showing a 1:1 (top; CSD entries: BEVMEU, PICOLI, PICOLI01, PUSYOV, PUSZEM, KAGWEY, MIDPEY, NULBEB, NULBIF, NULBIF01, PEWQIT, QARGIZ, QARGOF, RAKPAY, VUHDIN, YIPCEG, ZESQOG), 1:2 (CSD entries: AHONIY, BELJUP, CECZAL, CETJOE, LEWKOP, GUVDAG, NULCEC, NULBOL, QIHBOY, QIHBOY01, QIVPUJ) or 1:4 donor:I_2 stoichiometry (CSD entries: DARZAY).

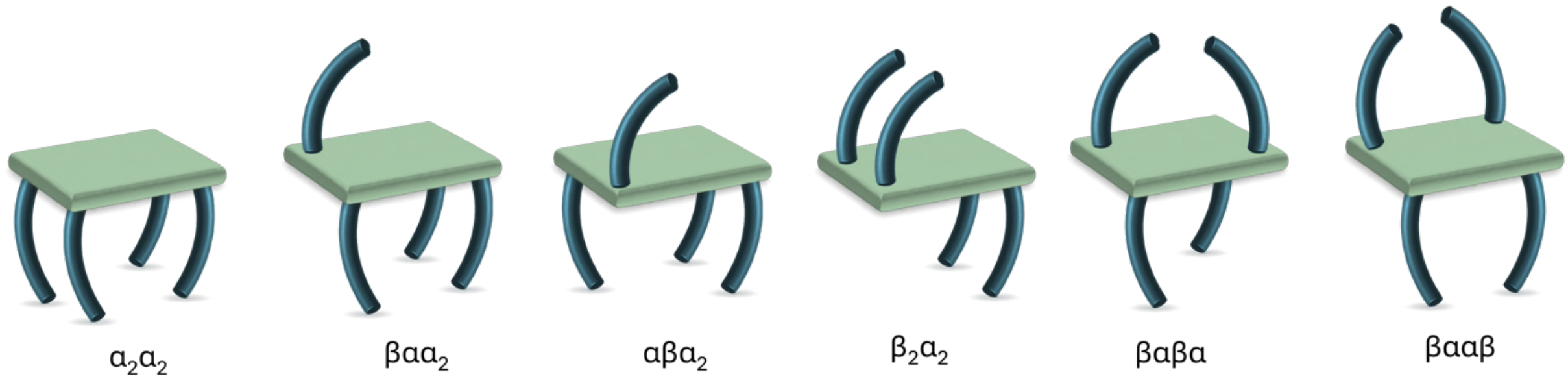

Scheme S3. Representation of the six possible conformers of **L**$_1$. The conformers $\beta\alpha\alpha_2$ and $\alpha\beta\alpha_2$ are isoenergetic.

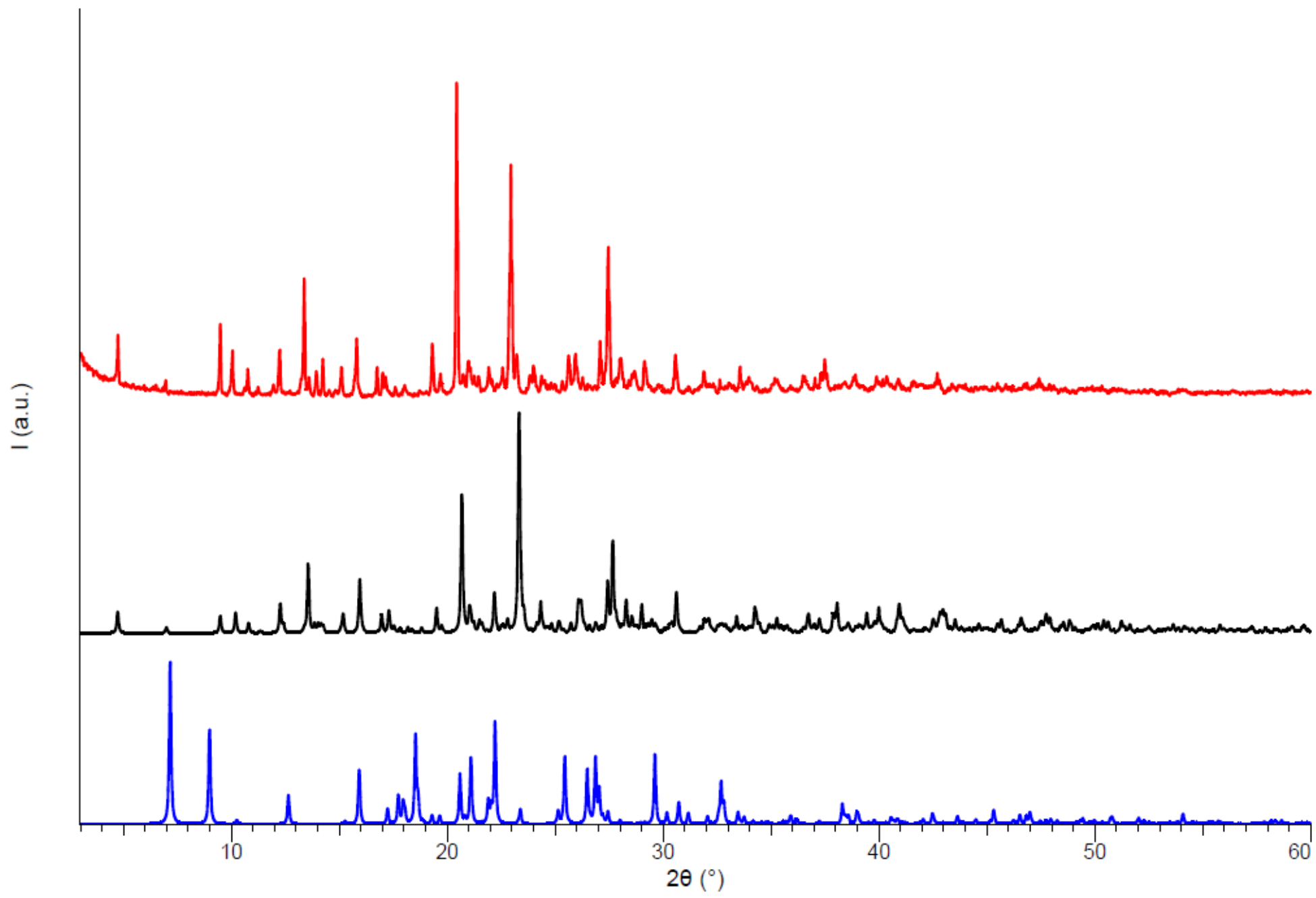

Figure S1. Powder X-ray diffraction analysis of compound **1** showing the comparison between the experimental pattern (red), the simulated pattern from SC-XRD data (black) and the simulated pattern for $\mathbf{L_1}$ (blue; CSD entry BORFEW).

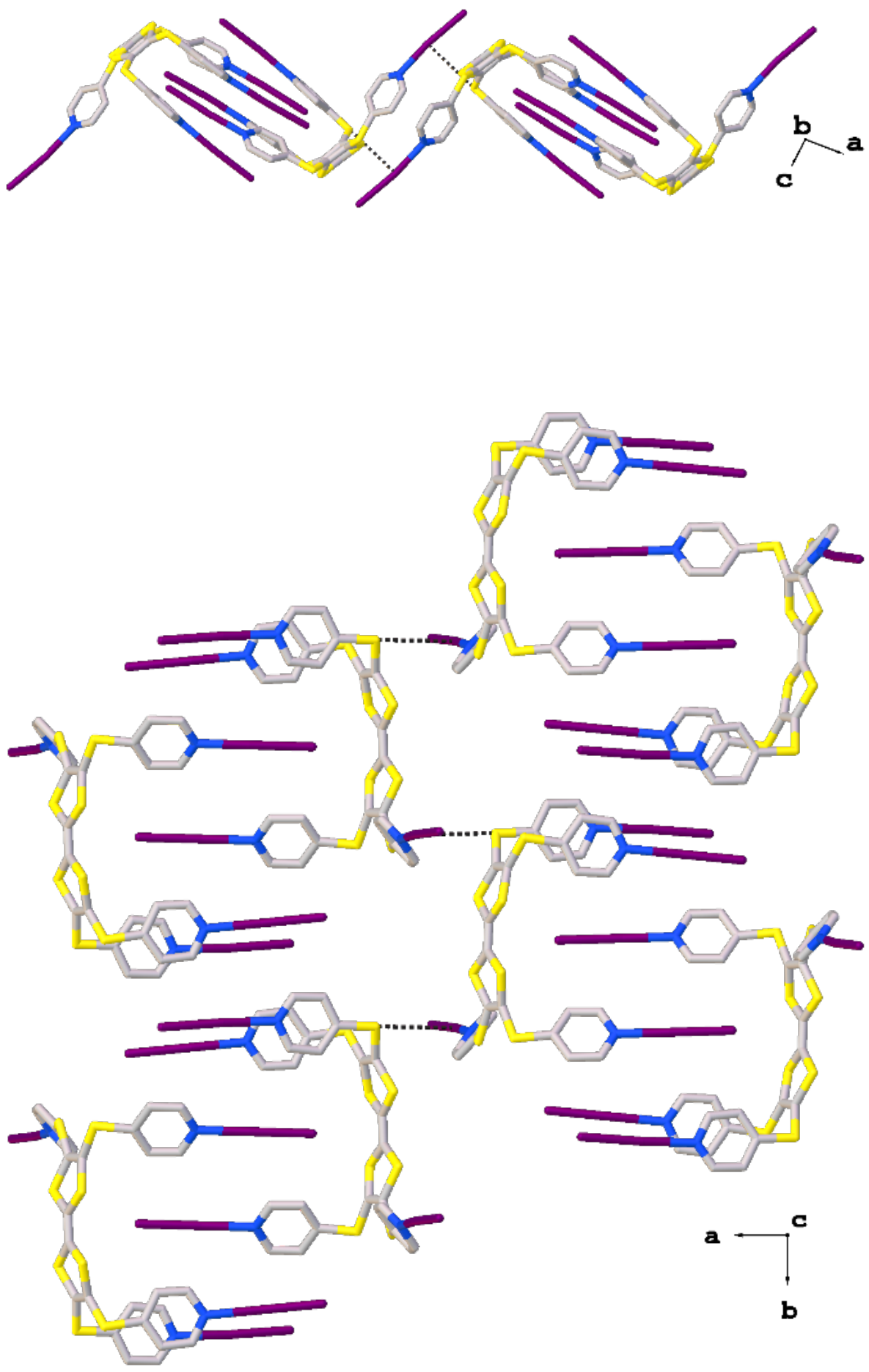

Figure S2. Partial views along the *b*- (top) and *c*-axis (bottom) of the crystal packing in compound **1** showing chalcogen-bonded interdigitated pairs of $\mathbf{L_1}\cdot 4I_2$ units. Hydrogen atoms were omitted for clarity.

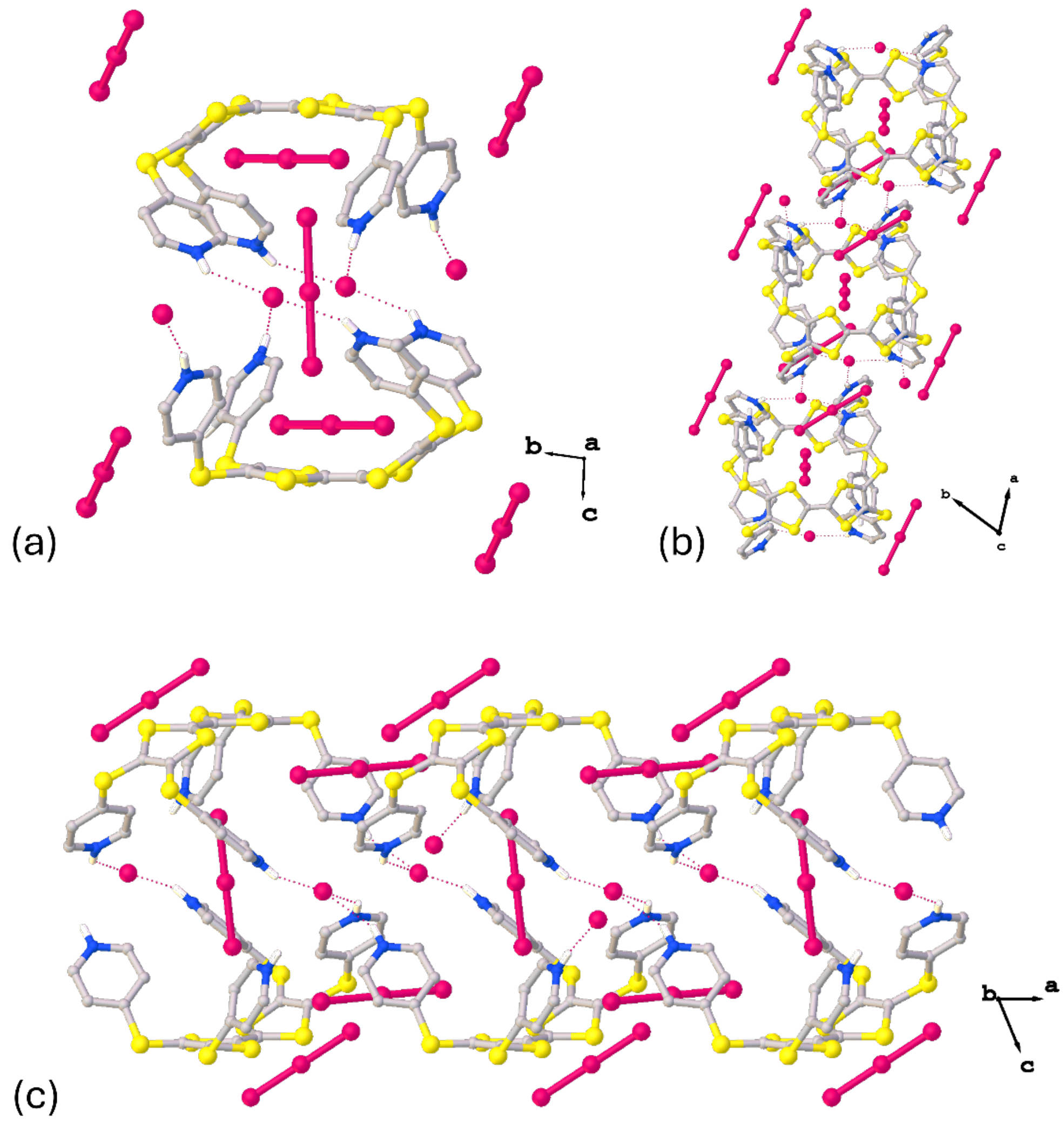

Figure S3. Partial views of the crystal packing of compound **2** seen along the *a*-, *c*-, and *b*-axis (a, b, and c, respectively). Solvent molecules and hydrogen atoms not involved in HBs were omitted for clarity.

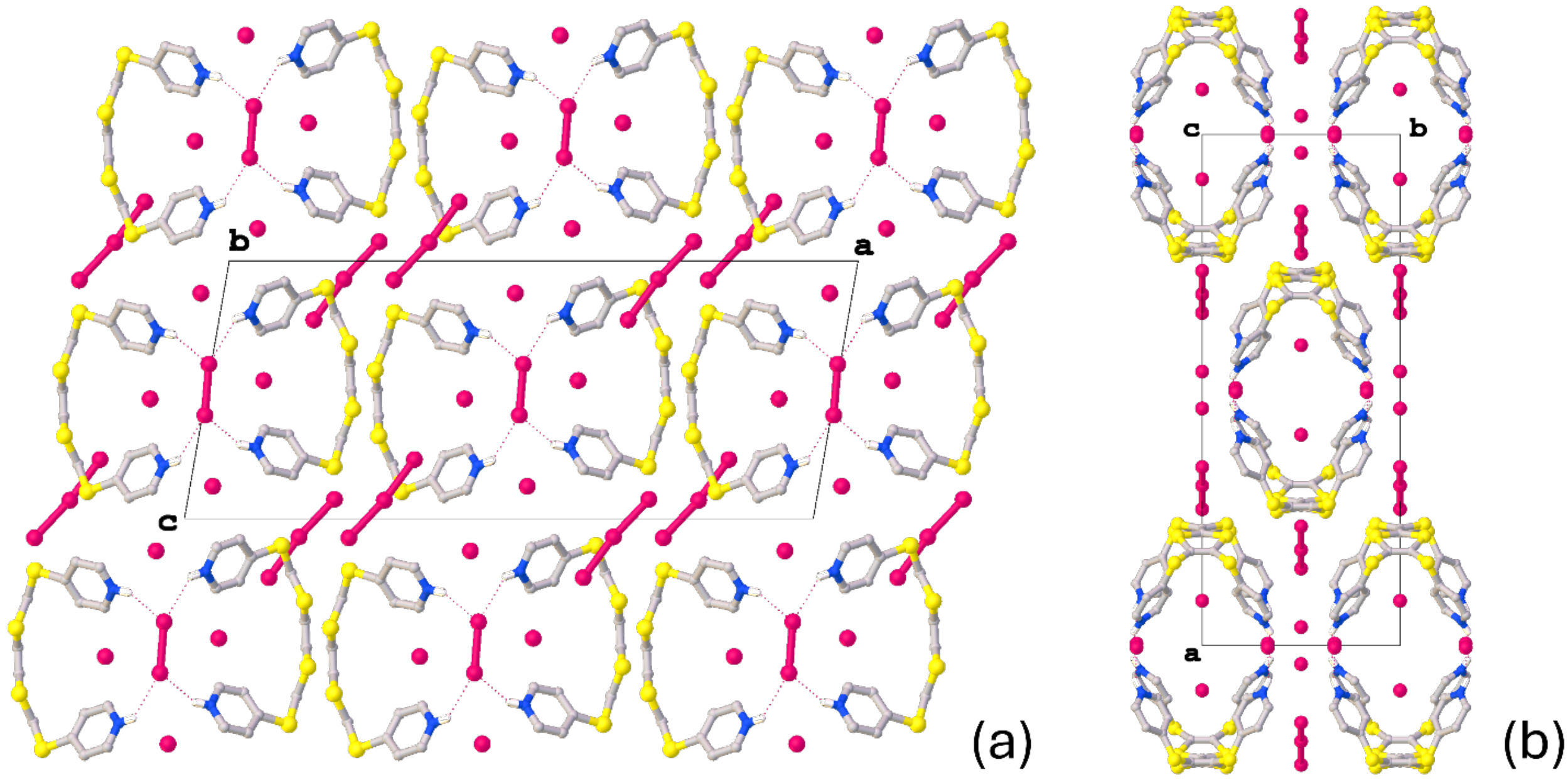

Figure S4. Partial views of the packing diagrams for compound **3** along the *b*- and *c*-axis (a and b, respectively). Solvent molecules, minor disorder components, hydrogen atoms not involved in HBs, and Br6-containing dibromine molecules were omitted for clarity.

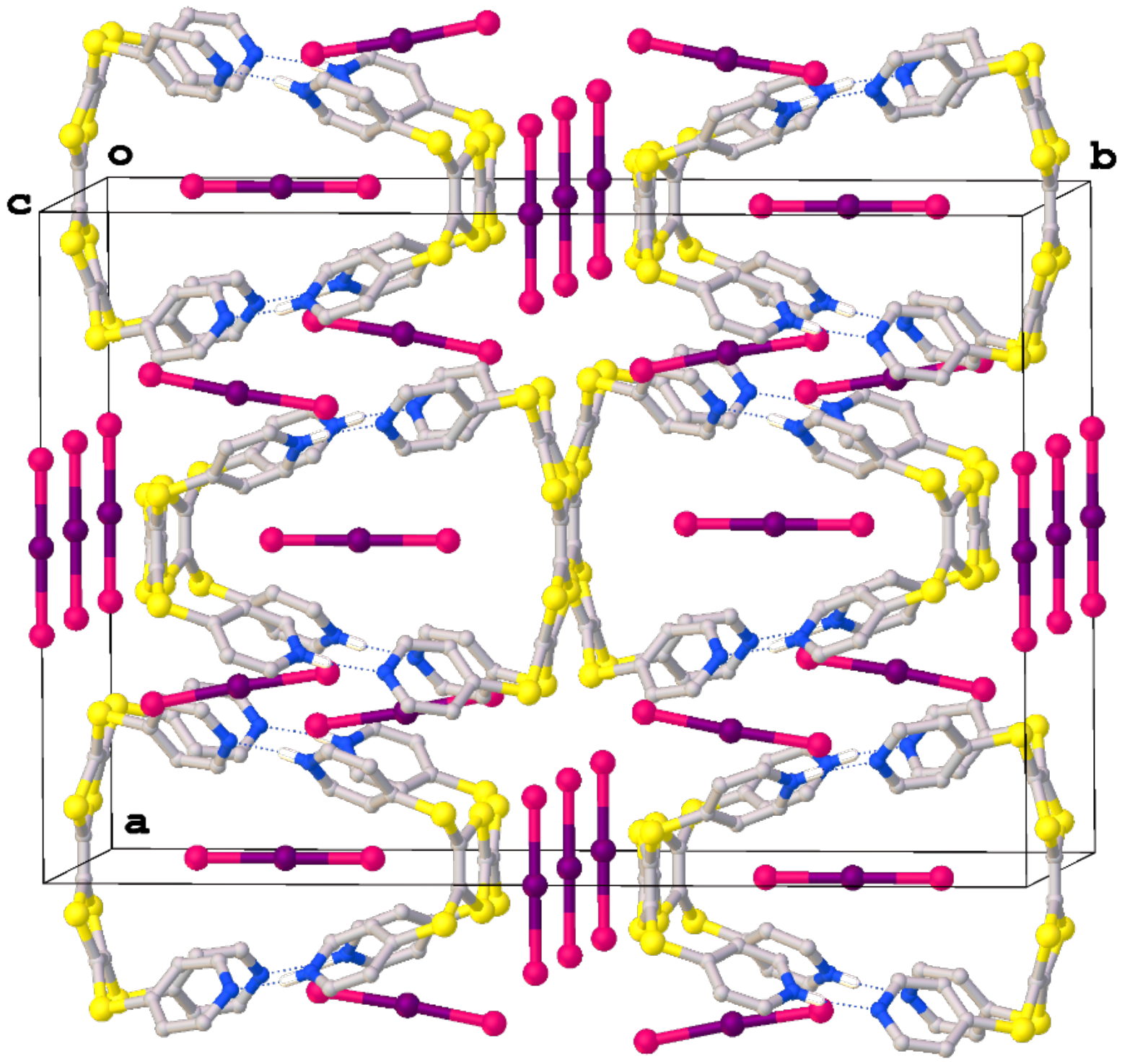

Figure S5. Portion of the crystal packing of compound **4**. Hydrogen atoms not involved in HBs were omitted for clarity.

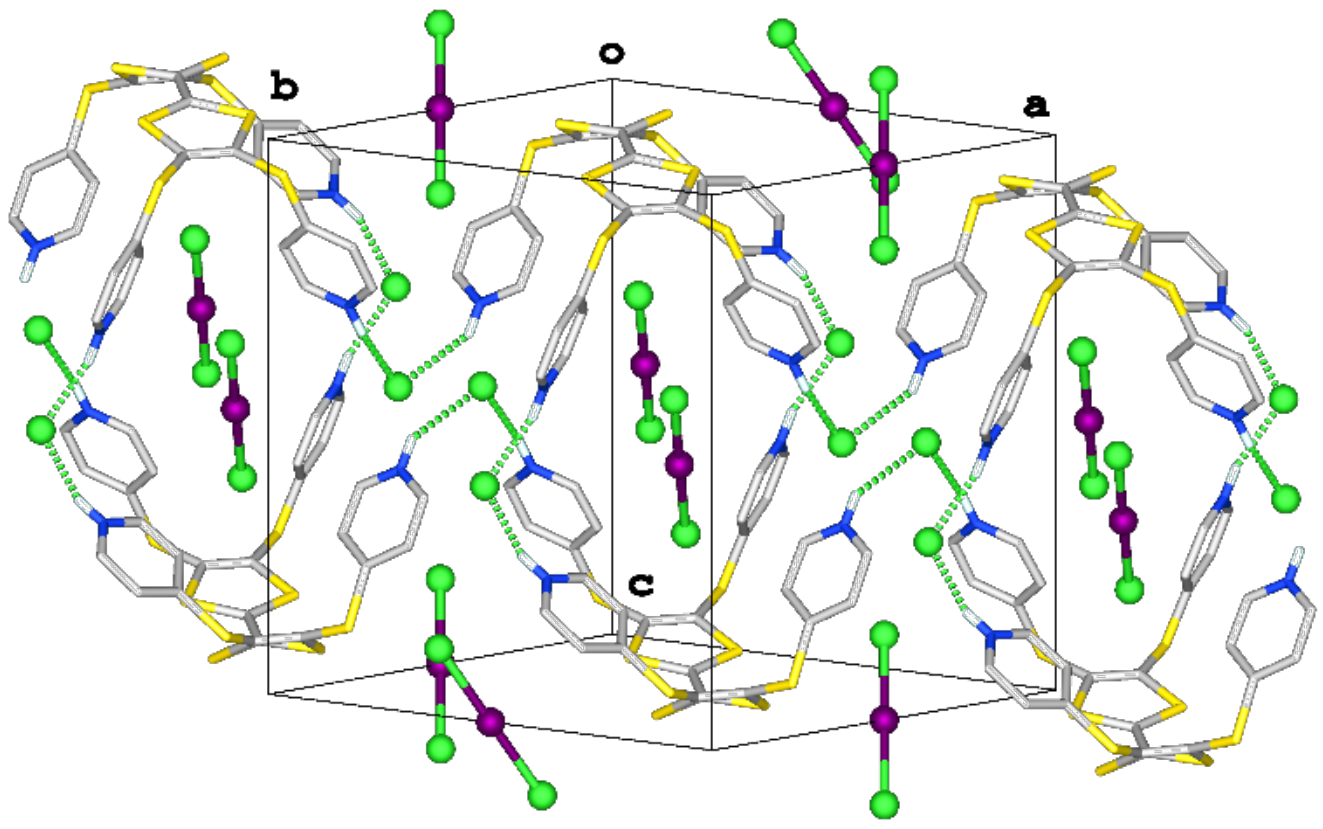

Single ribbon

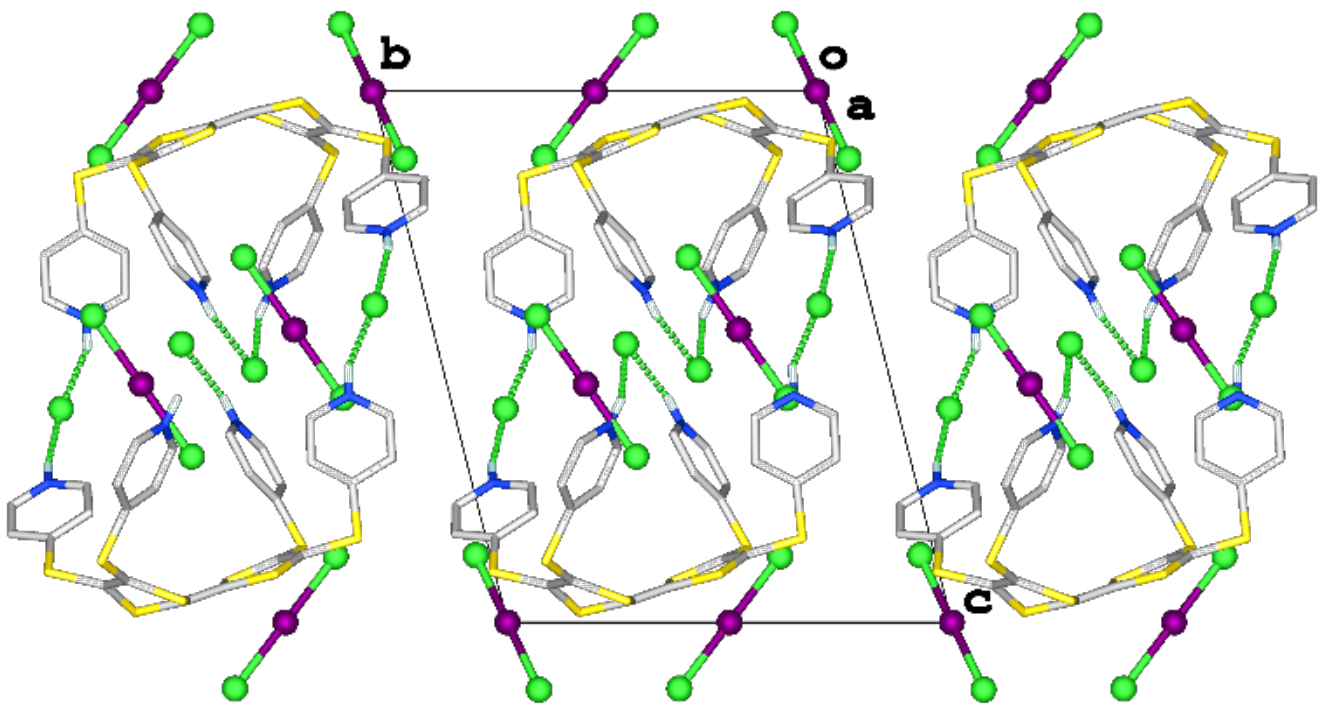

3 ribbons viewed along the b-axis.

Figure S6. Partial views of the packing diagram of compound **5**. Solvent molecules were omitted for clarity. Hydrogen atoms not involved in HBs were omitted for clarity.

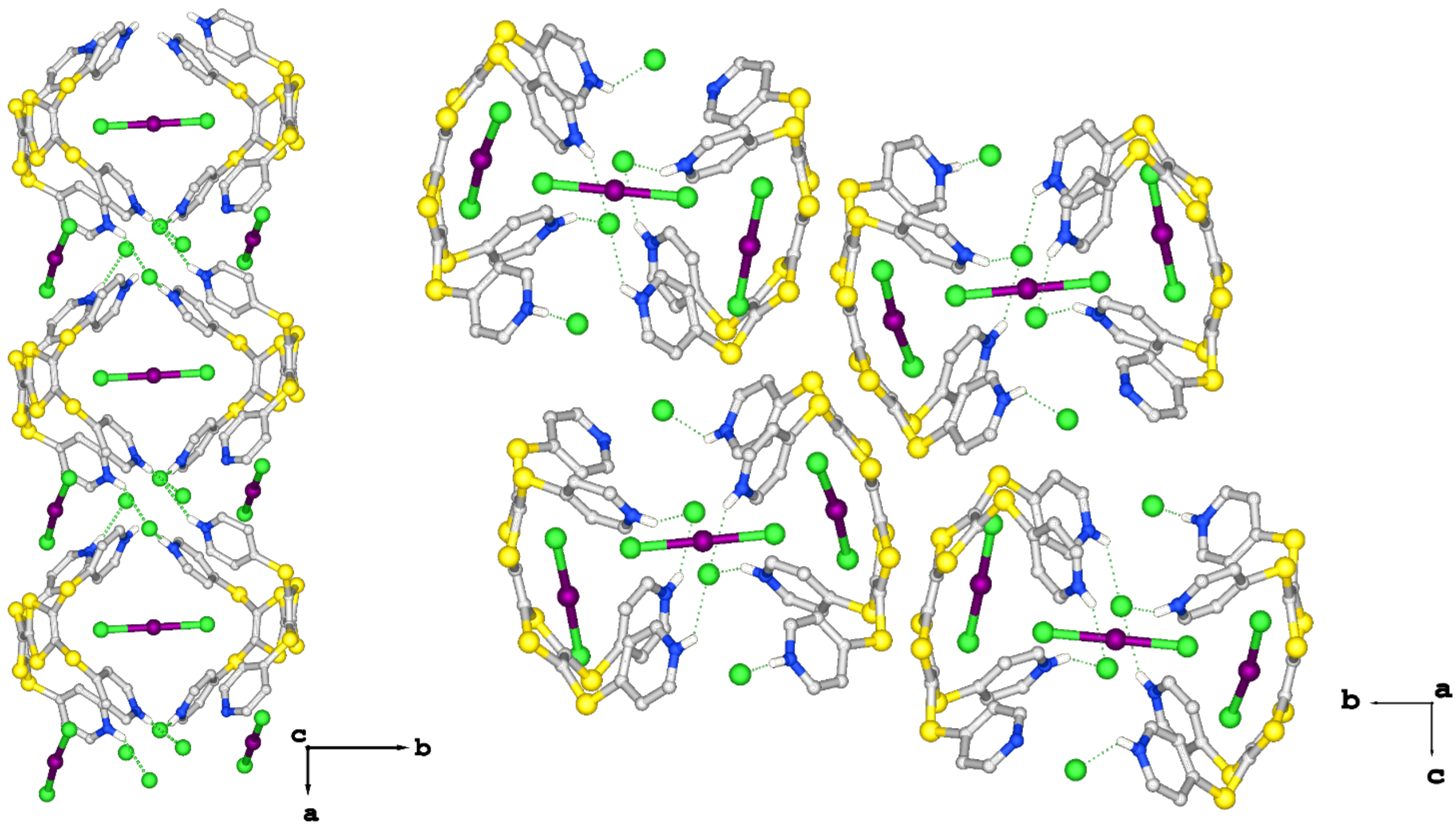

Figure S7. Partial views of the packing diagrams of **6** showing a single ribbon along the *c*-axis (left) and multiple ribbons propagating along the *a*-axis (right). Hydrogen atoms not involved in HBs were omitted for clarity.

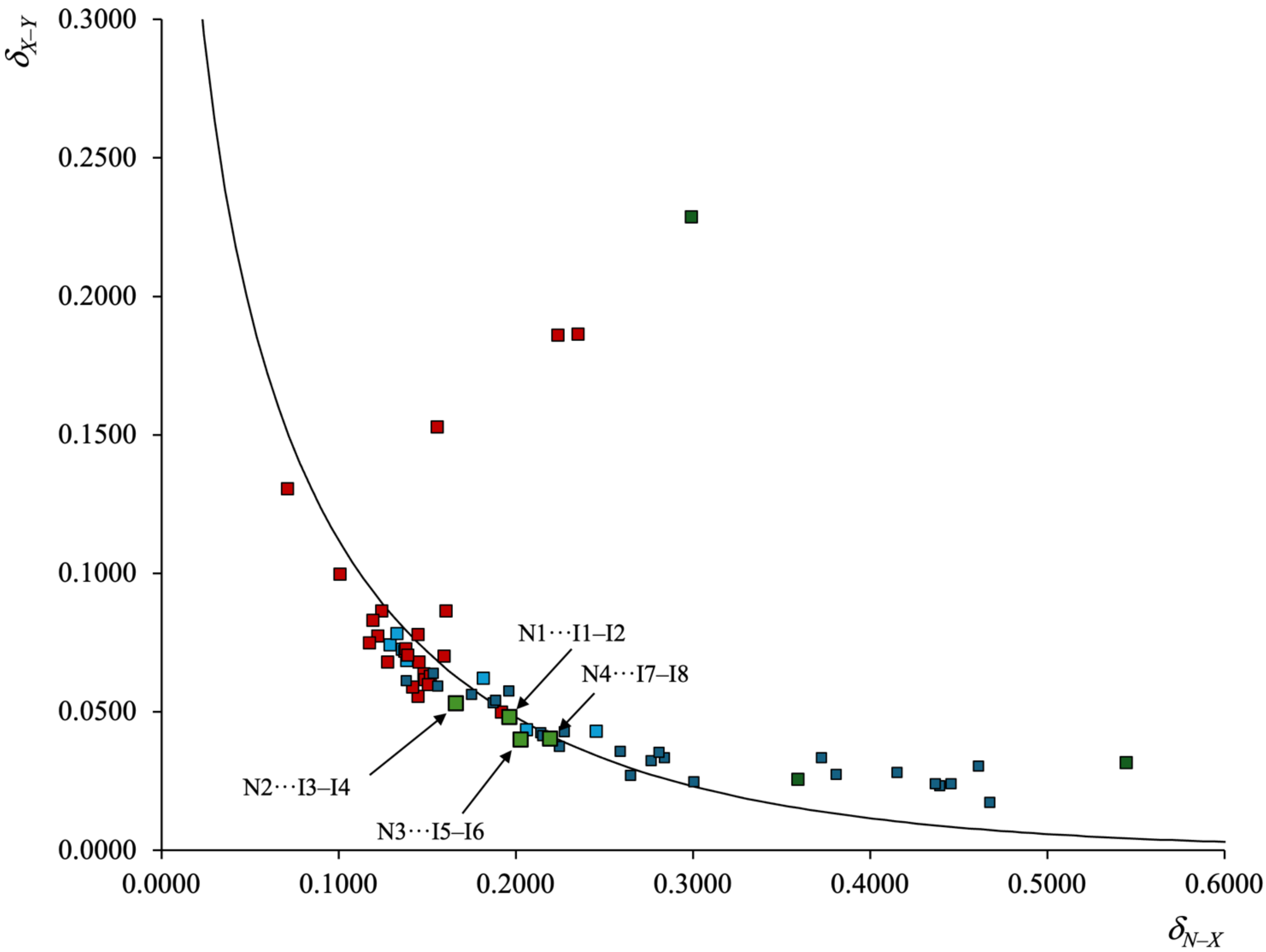

Figure S8. Structural data of the N···X−Y fragments found in the CSD reported as scatter plot of δ_{X-Y} vs δ_{N-X} (eq. 1). The solid curve represents the least-squares fit of the data adopting Eq. 2 as a model. Green squares: compound **1**; red squares: N···I–Cl; dark green squares: N···Br–Br; light blue squares: N···I–Br; dark blue squares: N···I–I. Fitted parameter k = 0.153.

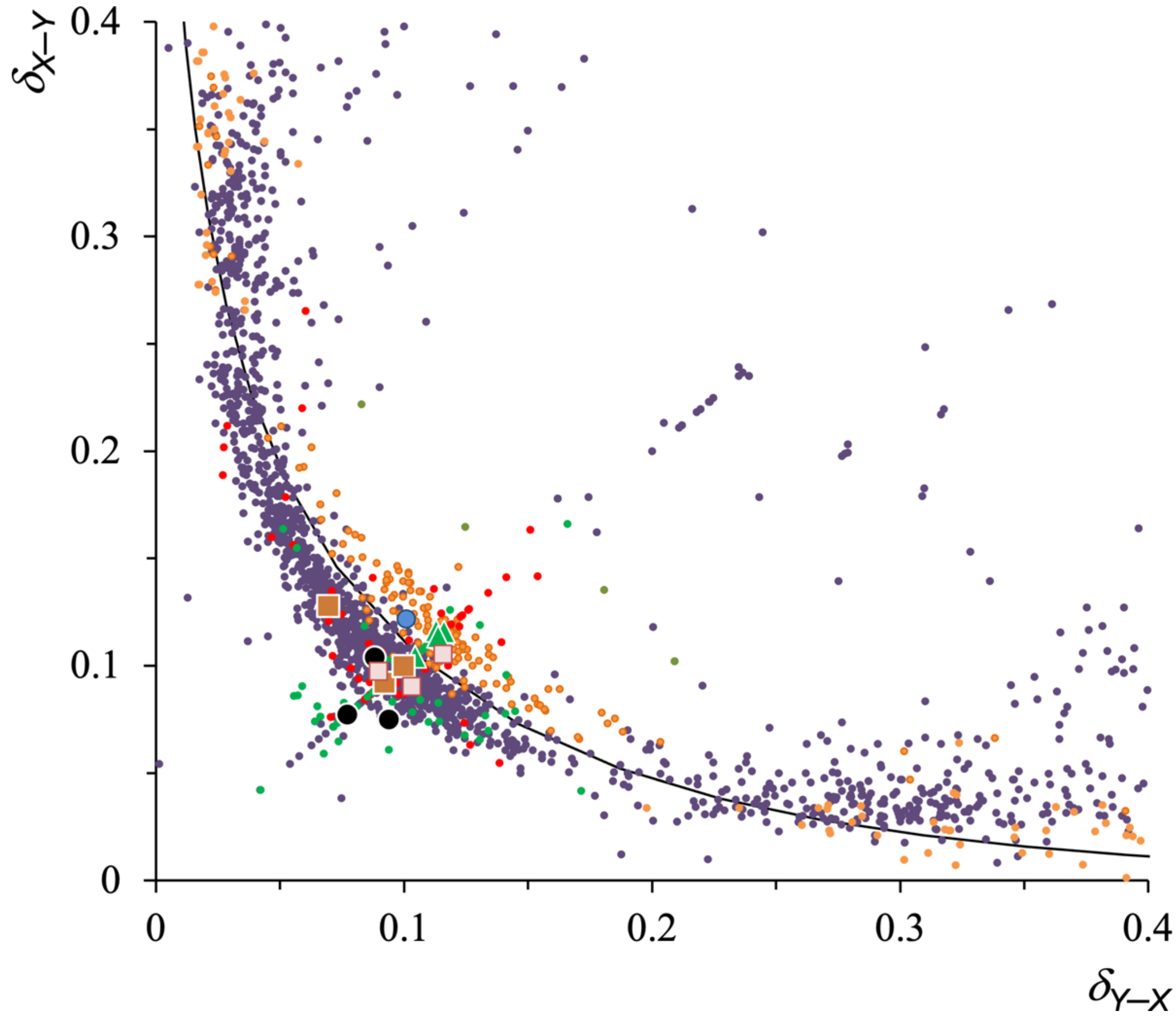

Figure S9. Structural data of the three-halogen sequences X–Y–X found in compounds **2**–**6** and in the crystal structures reported previously (see ref. 43 in the paper) as scatter plot of $\delta_{X\text{–}Y}$ vs $\delta_{Y\text{–}X}$ (eq. 1). The solid curve represents the least-squares fit of the data adopting eq. 2 as a model. Purple dots, I–I–I; orange dots, Br–Br–Br; dark green dots, Cl–Cl–Cl; light green dots, Cl–I–Cl; red dots, Br–I–Br; green triangles, $[Br_3]^-$ in compound **2**; cyan circles, $[Br_3]^-$ in compound **3**; black circles, $[IBr_2]^-$ in compound **4**; brown squares, $[ICl_2]^-$ in compound **5**; pink squares, $[ICl_2]^-$ in compound **6**. Fitted parameter $k = 0.152$.

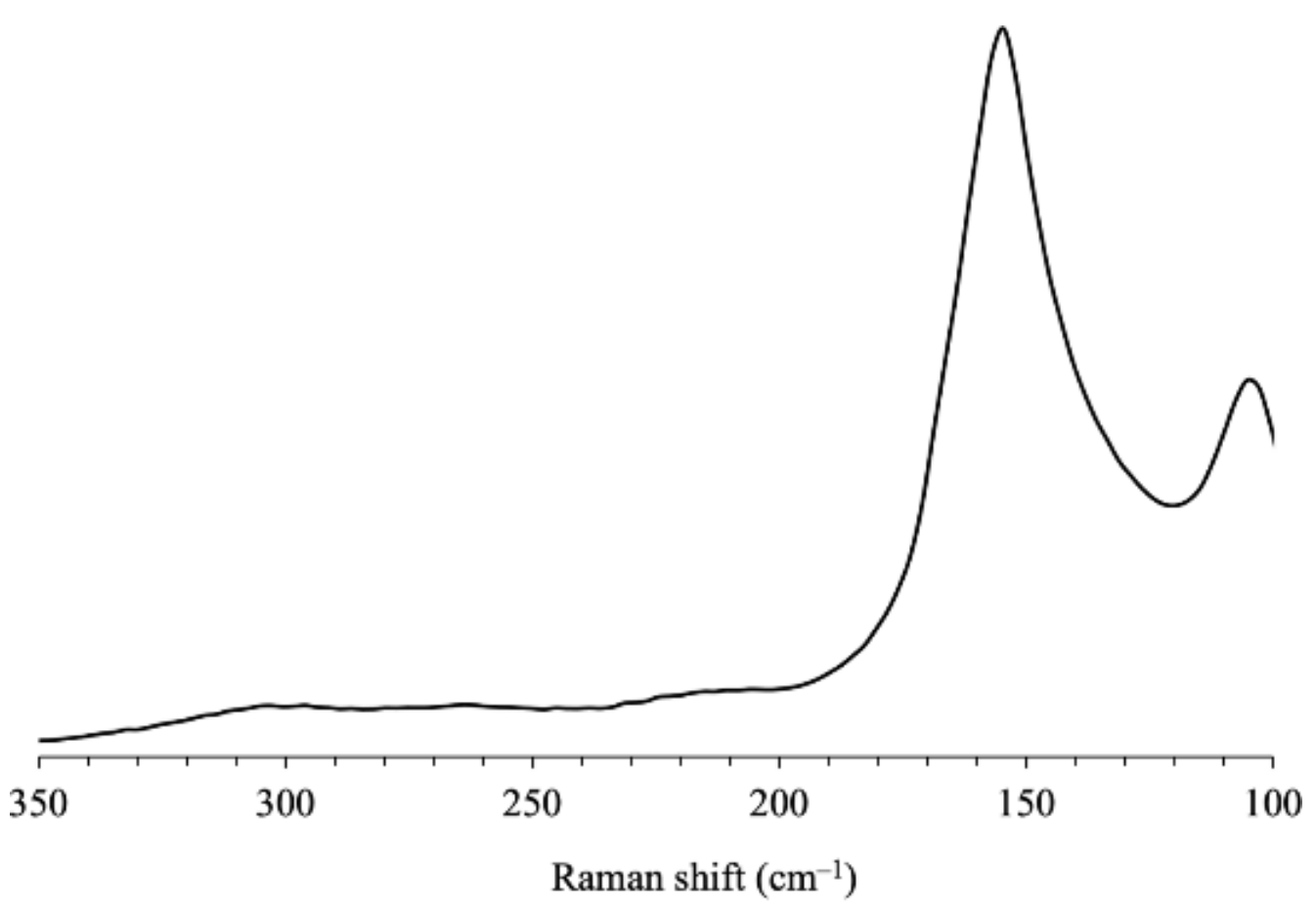

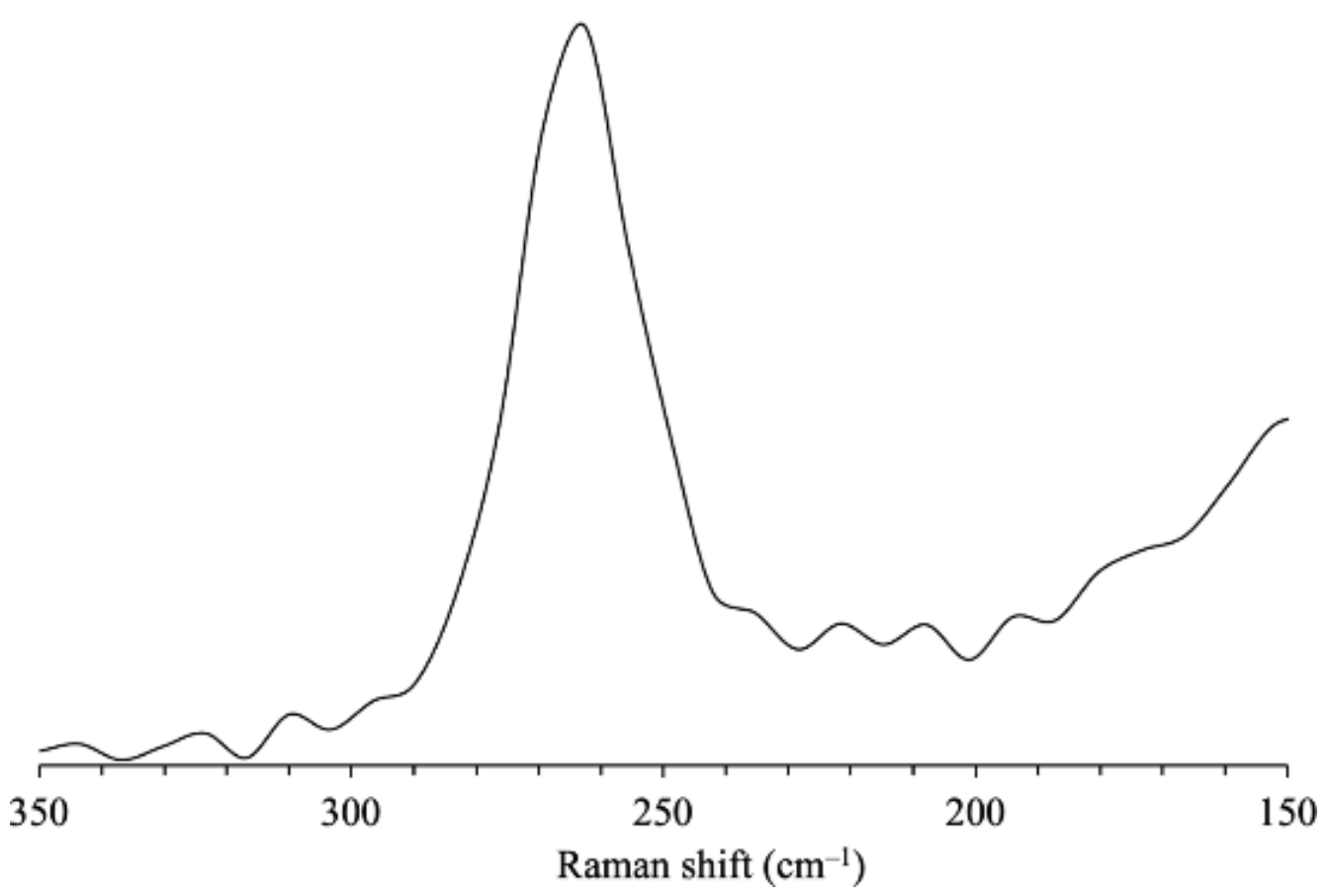

Figure S10. Solid state Raman spectrum recorded at r.t. for compounds **1** (top; 100–350 cm^{-1}) and **5** (bottom; 150–350 cm^{-1}).

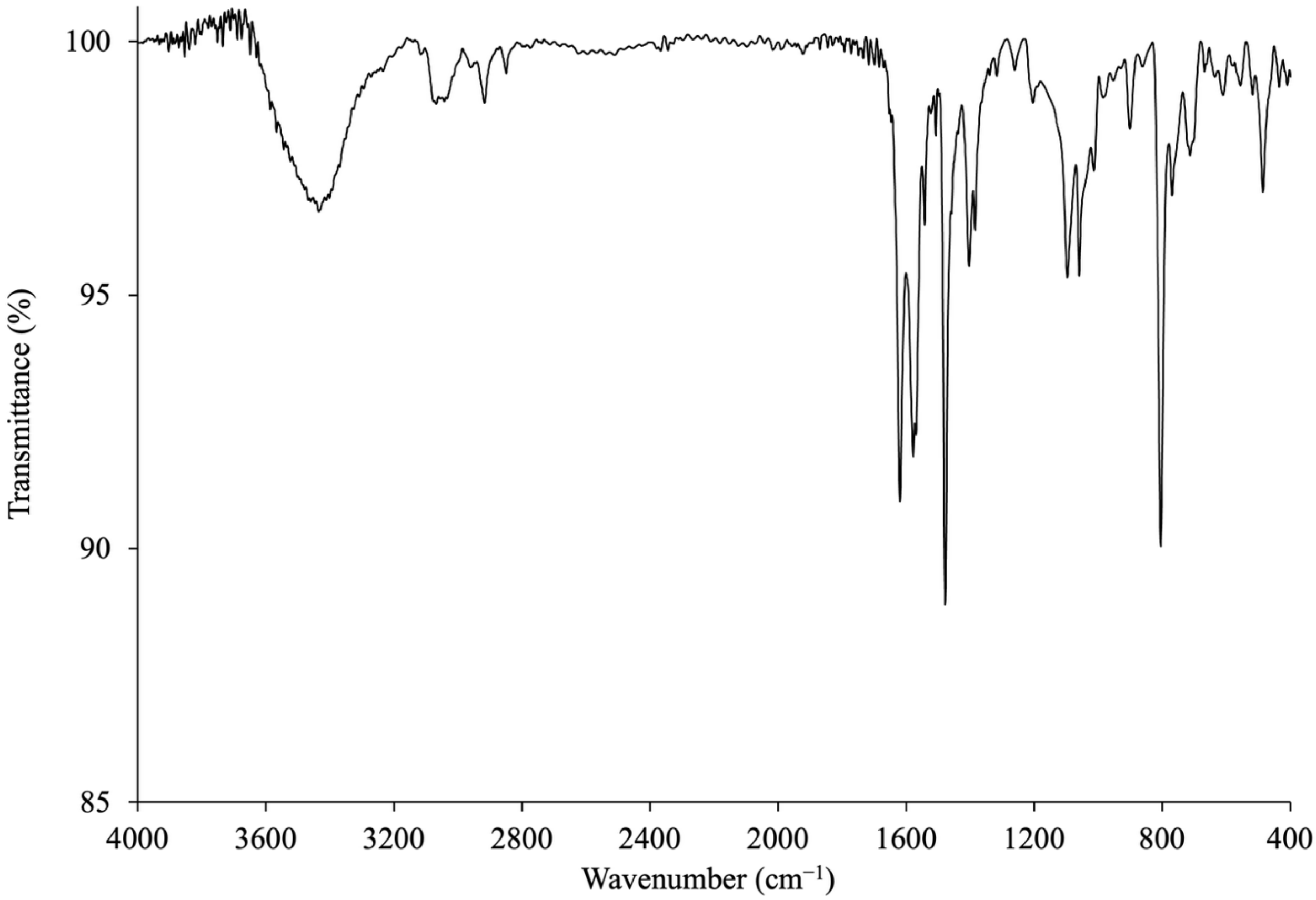

Figure S11. FT–IR spectrum (400–4000 cm^{-1}; KBr pellets) of compound **1**.

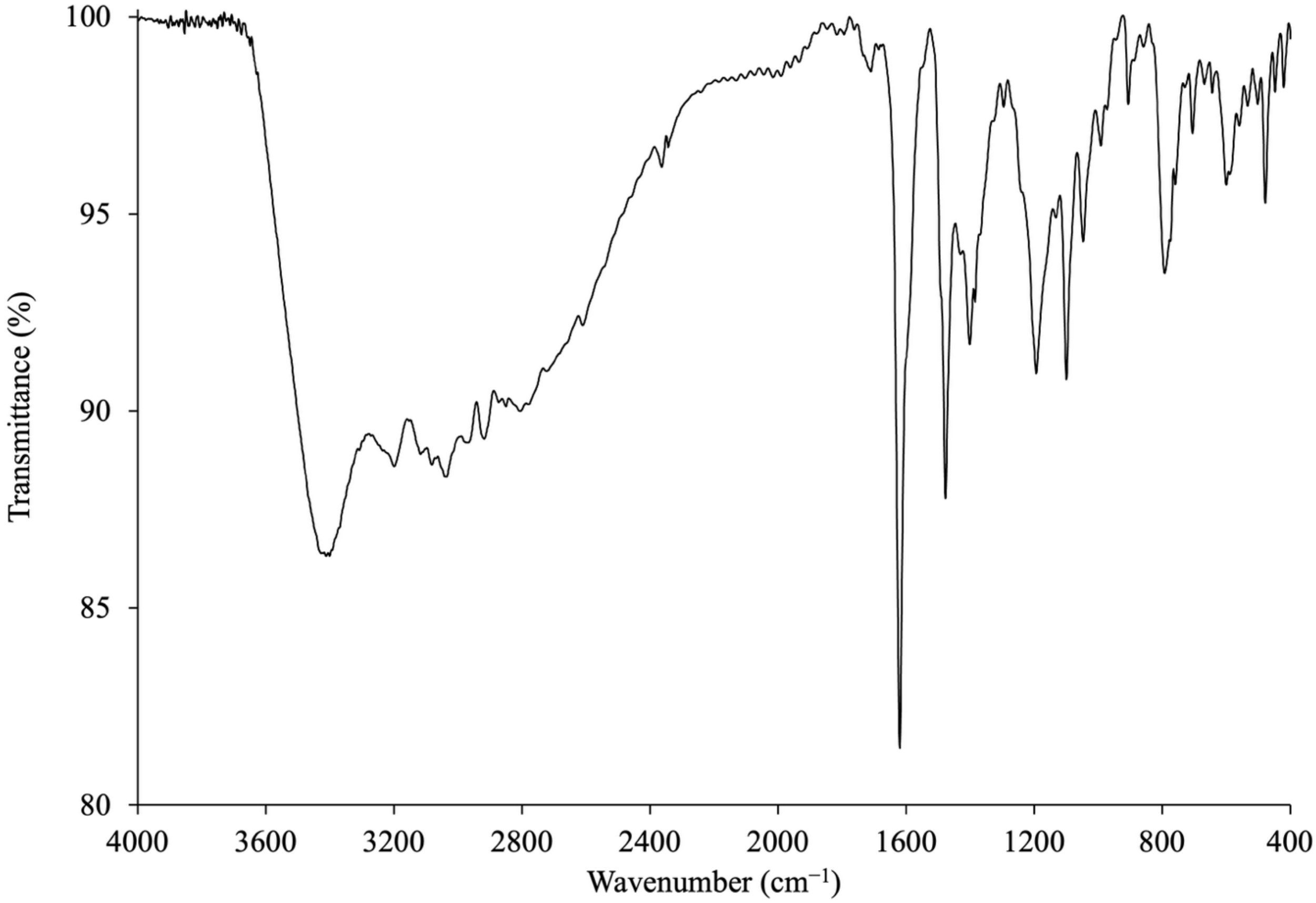

Figure S12. FT–IR spectrum (400–4000 cm^{-1}; KBr pellets) of compound **2**.

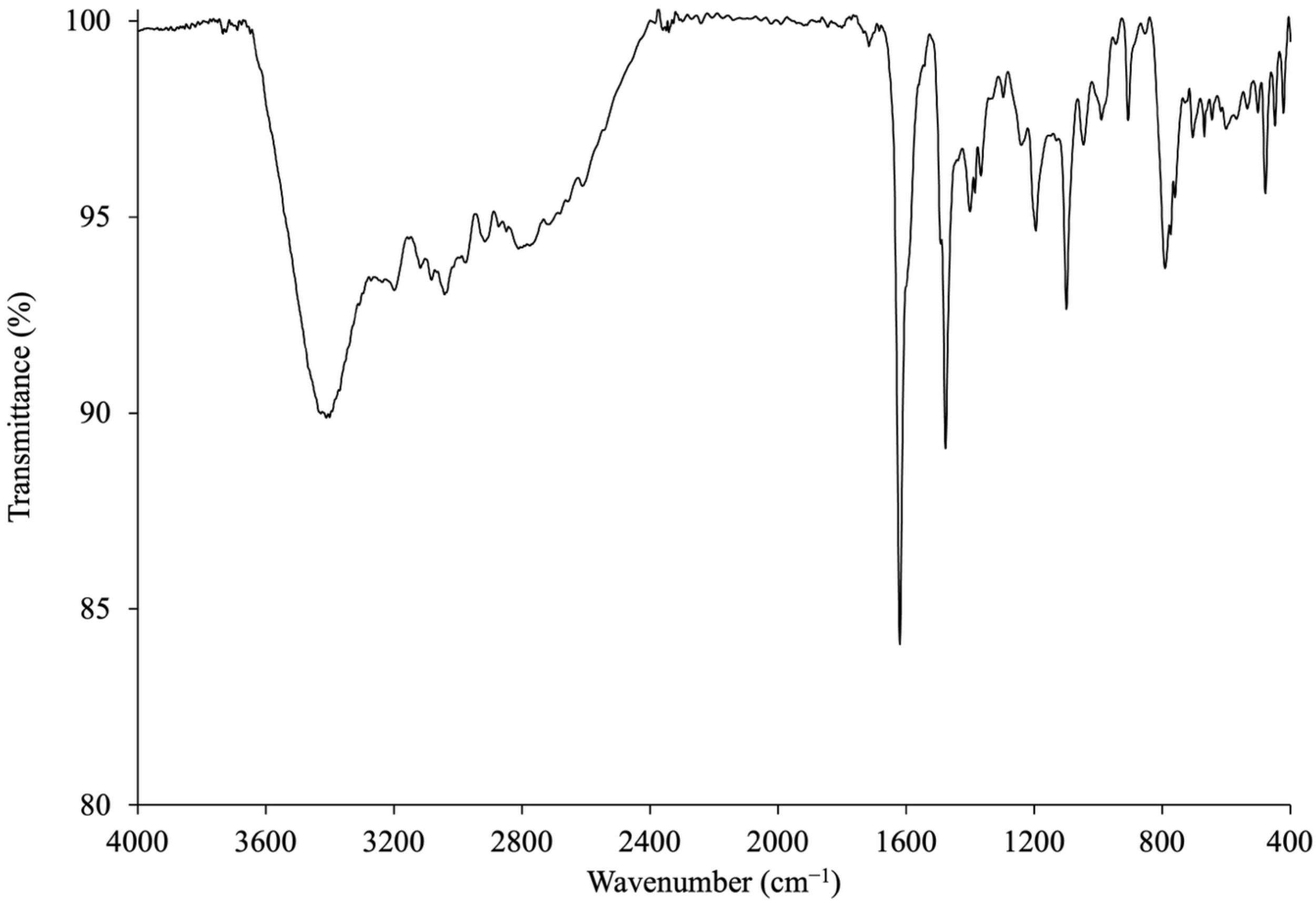

Figure S13. FT–IR spectrum (400–4000 cm^{-1}; KBr pellets) of compound **3**.

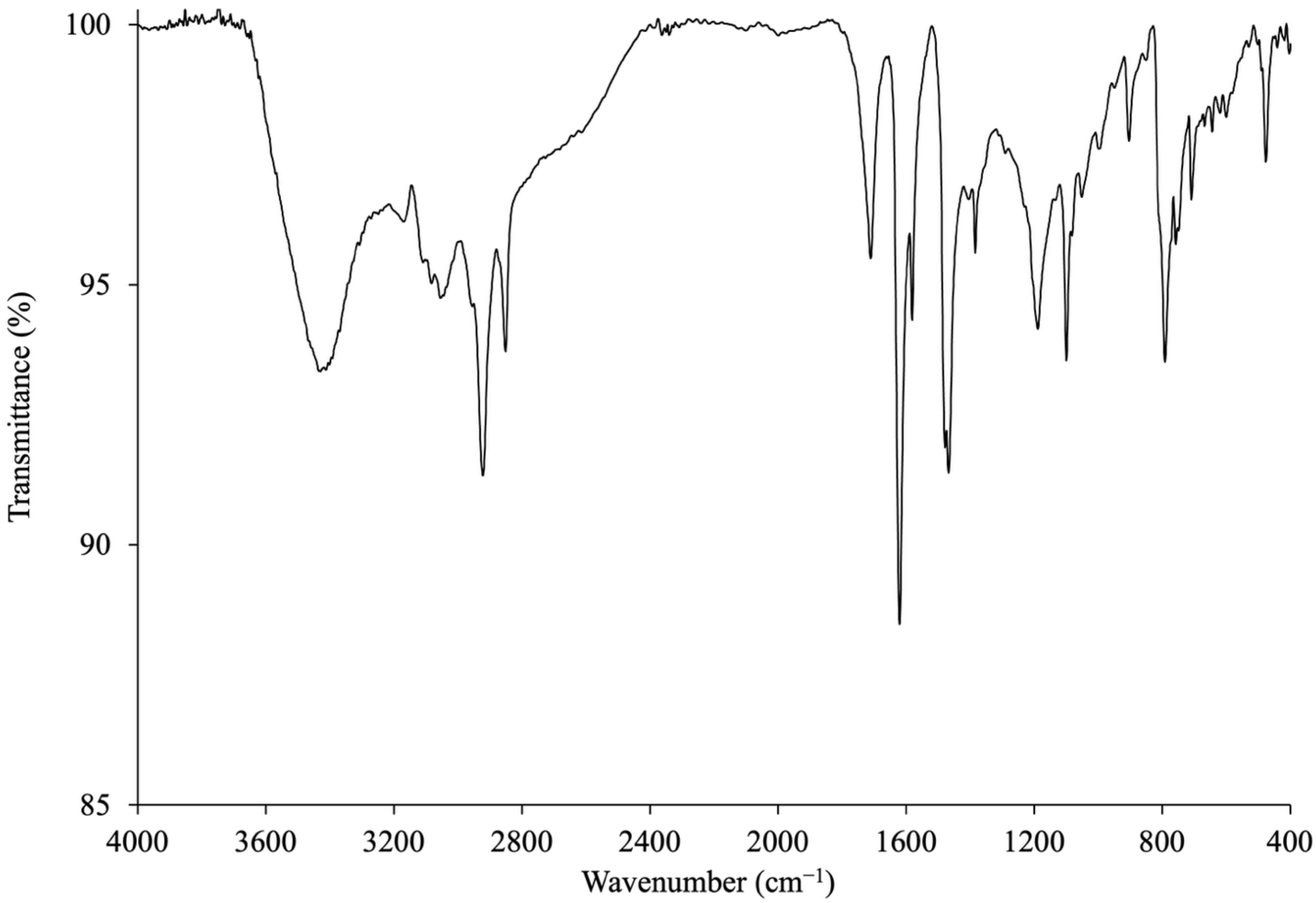

Figure S14. FT–IR spectrum (400–4000 cm^{-1}; KBr pellets) of compound **4**.

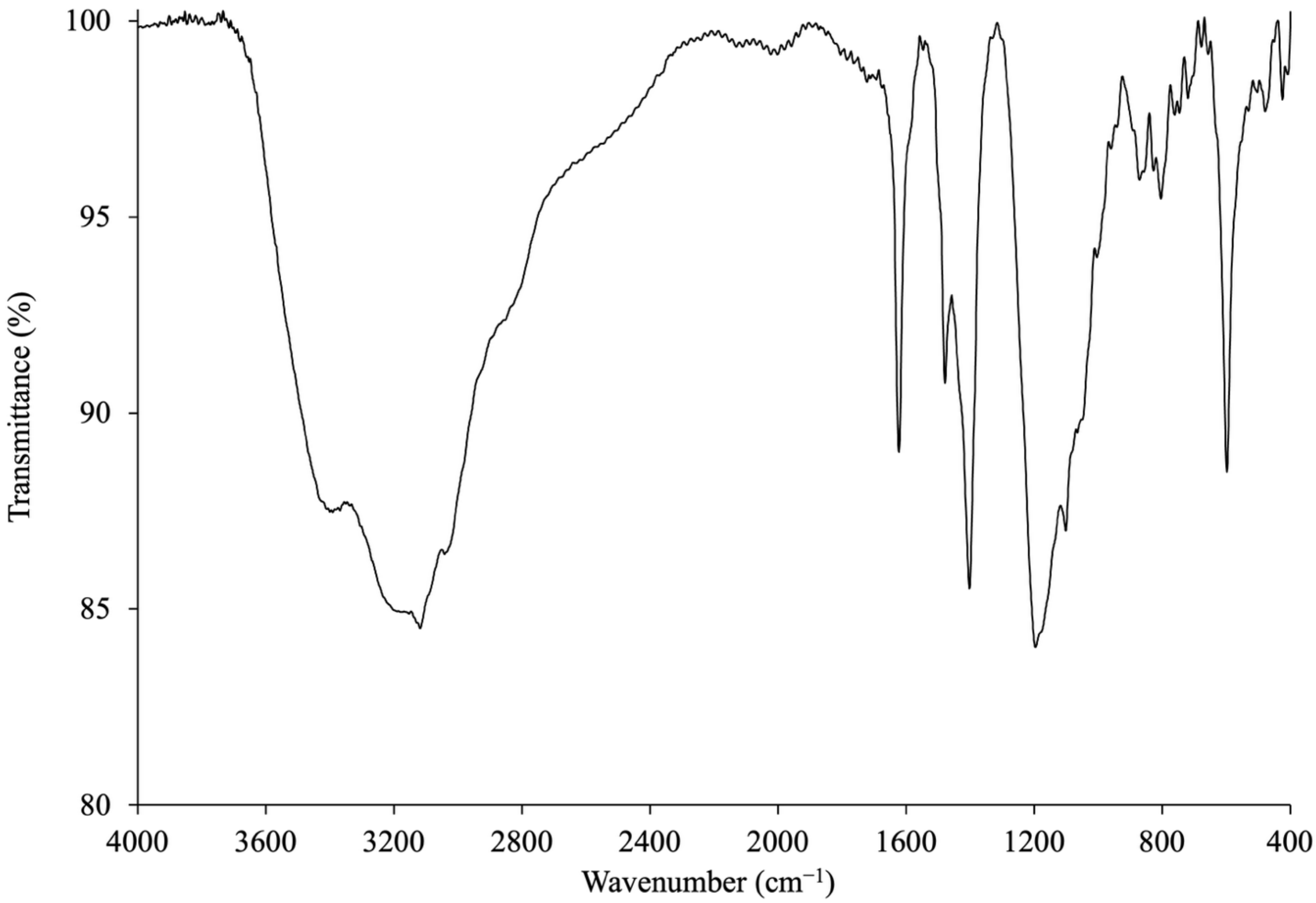

Figure S15. FT–IR spectrum (400–4000 cm^{-1}; KBr pellets) of compound **5**.

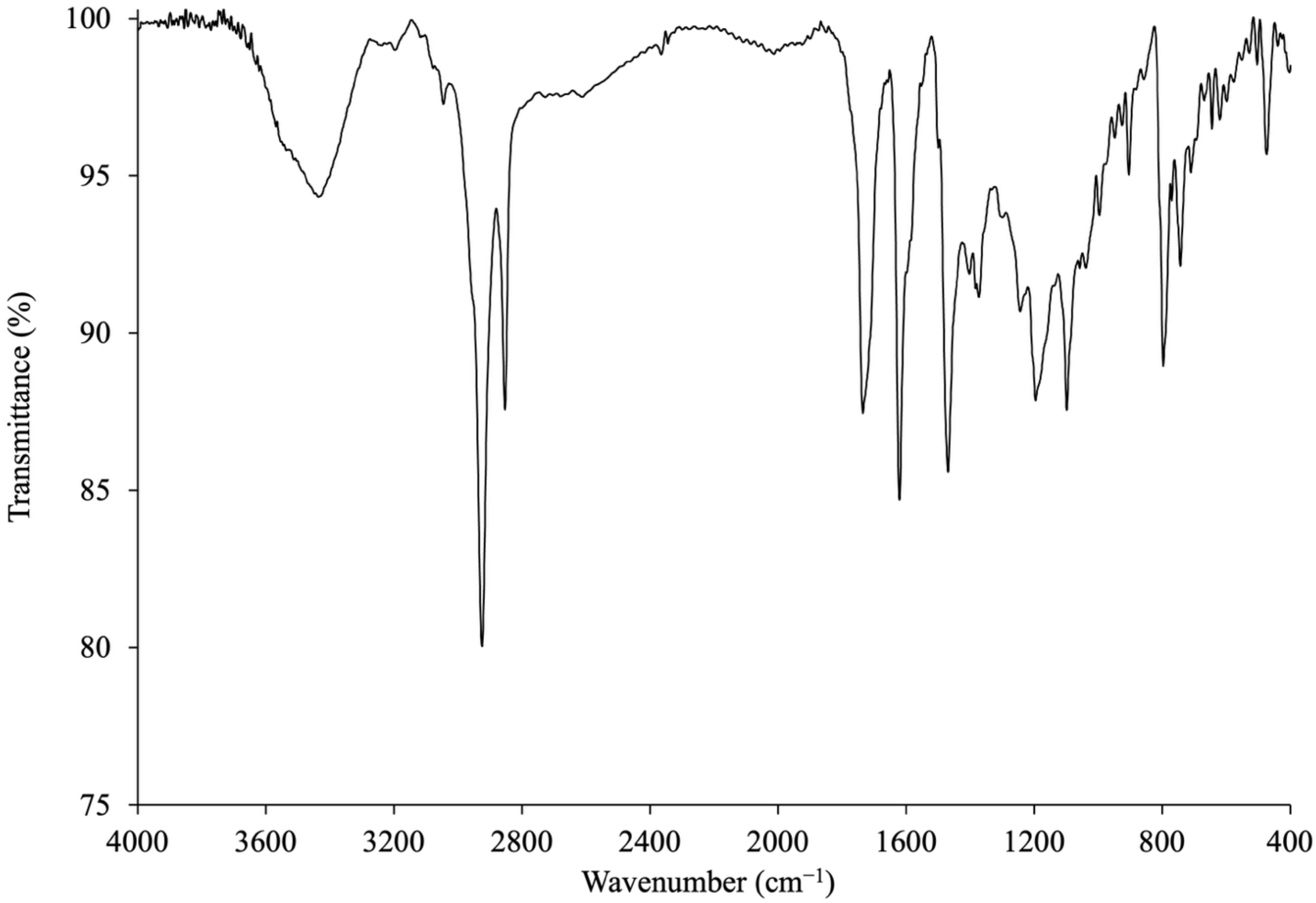

Figure S16. FT–IR spectrum (400–4000 cm^{-1}; KBr pellets) of compound **6**.

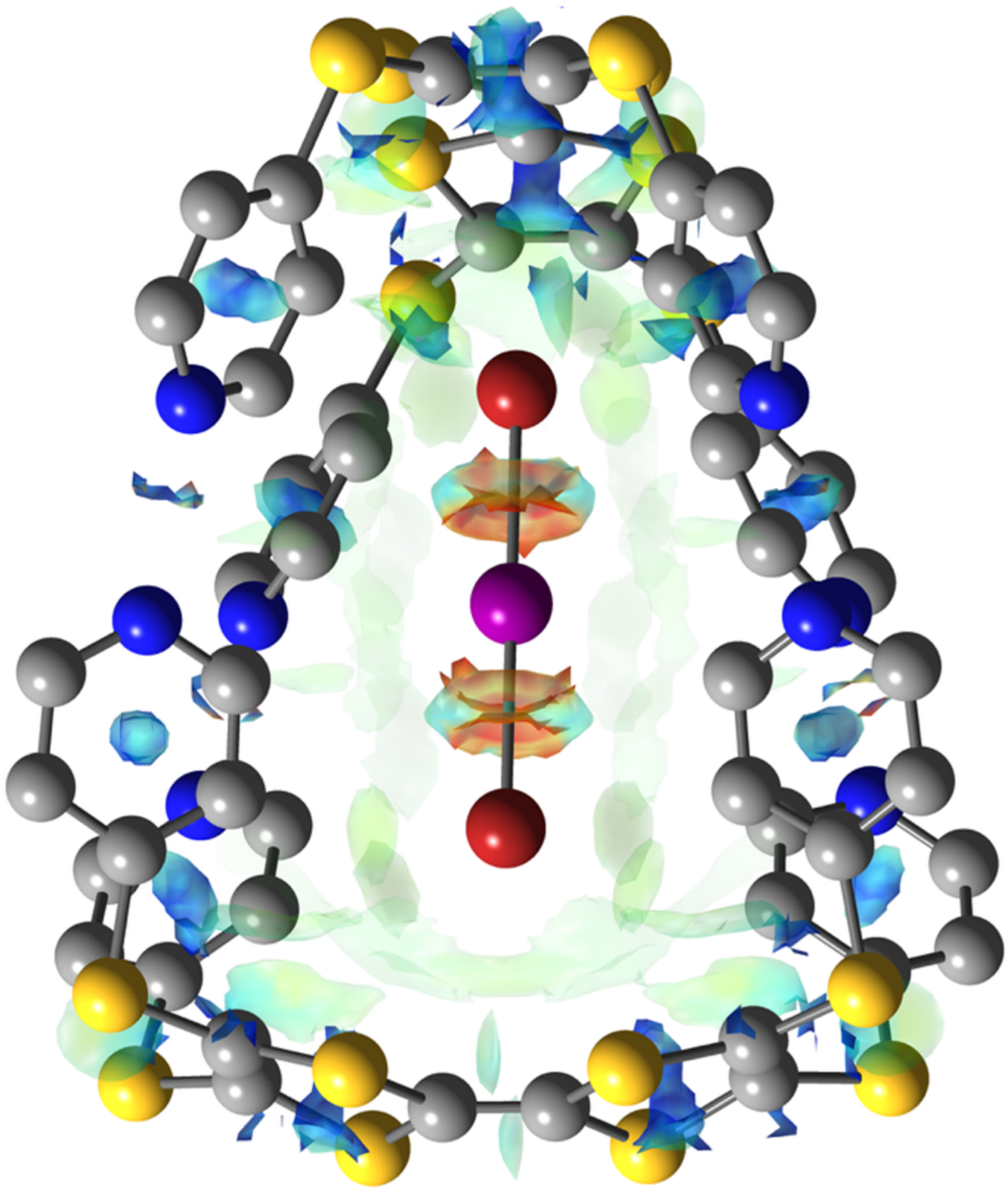

Figure S17. Color-filled RDG isosurface calculated for $[(H_4\mathbf{L_1})(\mathbf{L_1})(IBr_2)]^{3+}$ at the DFT-optimized geometry (functional mPW1PW/Def2TZVP (C, H, N, S)/LANL08(d) (Br, I)). Hydrogen atoms were omitted for clarity.

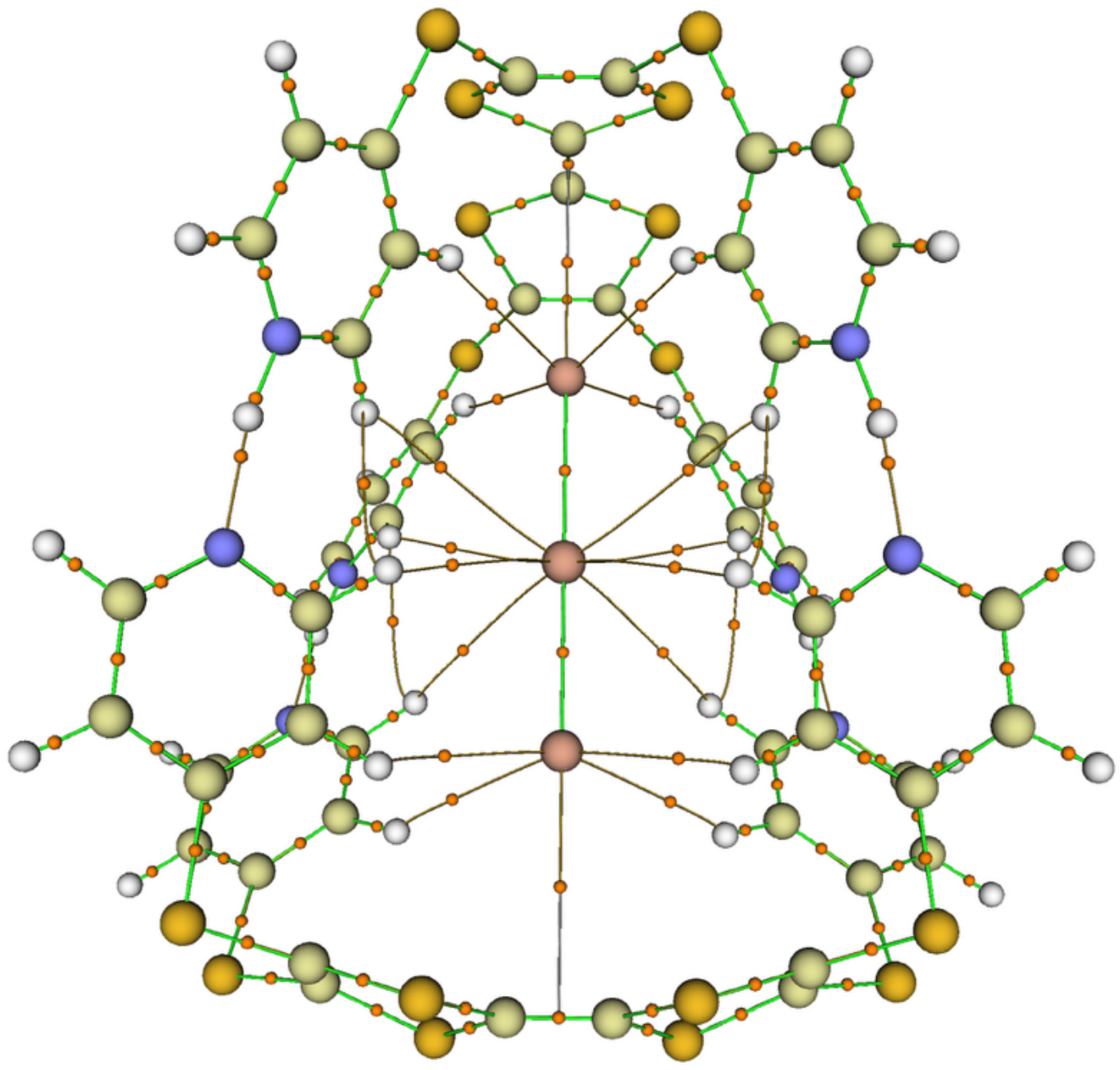

Figure S18. BCPs of type (3,–1) and bond paths calculated QTAIM level for the prismatic fragment $[(H_4\mathbf{L_1})(\mathbf{L_1})(IBr_2)]^{3+}$ at the DFT-optimized geometry (functional mPW1PW/Def2TZVP (C, H, N, S)/LANL08(d) (Br, I)).
